# Straightforward synthesis of complex polymeric architectures with ultra-high chain density†

**DOI:** 10.1039/d4sc01739k

**Published:** 2024-07-12

**Authors:** Sachin Gupta, Miroslav Janata, Eva Čadová, Vladimír Raus

**Affiliations:** a Institute of Macromolecular Chemistry, Czech Academy of Sciences Heyrovského nám. 2 162 06 Prague 6 Czech Republic raus@imc.cas.cz

## Abstract

Synthesis of complex polymeric architectures (CPAs) *via* reversible-deactivation radical polymerization (RDRP) currently relies on the rather inefficient attachment of monofunctional initiation/transfer sites onto CPA precursors. This drawback seriously limits the overall functionality of the resulting (macro)initiators and, consequently, also the total number of installable polymeric chains, which represents a significant bottleneck in the design of new polymeric materials. Here, we show that the (macro)initiator functionality can be substantially amplified by using trichloroacetyl isocyanate as a highly efficient vehicle for the rapid and clean introduction of trichloroacetyl groups (TAGs) into diverse precursors. Through extensive screening of polymerization conditions and comprehensive NMR and triple-detection SEC studies, we demonstrate that TAGs function as universal trifunctional initiators of copper-mediated RDRP of different monomer classes, affording low-dispersity polymers in a wide molecular weight range. We thus unlock access to a whole new group of ultra-high chain density CPAs previously inaccessible *via* simple RDRP protocols. We highlight new opportunities in CPA synthesis through numerous examples, including the *de novo* one-pot synthesis of a novel “star-on-star” CPA, the preparation of β-cyclodextrin-based 45-arm star polymers, and facile grafting from otherwise problematic cellulose substrates both in solution and from surface, obtaining effortlessly ultra-dense, ultra-high-molecular weight bottle-brush copolymers and thick spatially-controlled polymeric coatings, respectively.

## Abstract 2

The application of trichloroacetyl isocyanate (TAI) adducts as initiators of copper-mediated reversible-deactivation radical polymerization (Cu-RDRP) unlocks straightforward access to complex polymeric architectures with ultra-high chain density.

## Graphical abstract

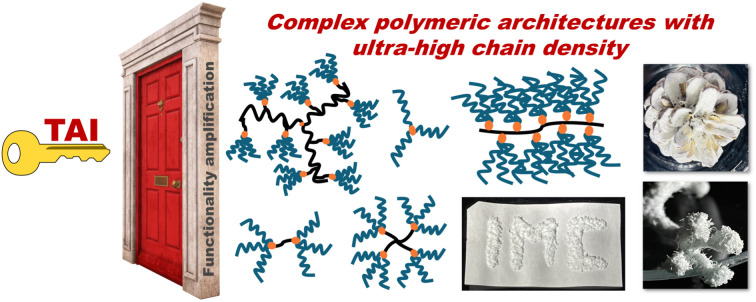

## Introduction

Complex polymeric architectures (CPAs), such as star,^1^ dendrimer,^2^ graft,^3^ bottle-brush,^4^ or hyperbranched^5^ (co)polymers, are characterized by an additional layer of intricacy endowing these polymeric objects with unique physical properties and an ability to self-assemble into higher-order structures. Owing to their intriguing features, CPAs have found multiple applications in diverse fields, including drug delivery,^6–9^ bioimaging,^10^ catalysis,^11^ nanotemplating,^12–14^ photonics,^15^ or super-elastomers.^16–18^

Reversible-deactivation radical polymerization (RDRP) methods, and particularly copper-mediated RDRP (Cu-RDRP) and reversible addition–fragmentation chain transfer (RAFT), represent powerful tools for precisely controlling composition, functionality, and topology of polymeric chains, enabling thus a straightforward access to unique CPAs otherwise unattainable with conventional polymerization techniques.^19,20^ In the key step of CPA synthesis *via* RDRP, a CPA precursor is decorated with specific functionalities, such as initiators in Cu-RDRP or transfer agents in RAFT, that predetermine the sites of the future polymer chain attachment or growth. The concentration and distribution of these sites within the precursor is essential for determining key CPA characteristics, such as grafting density in graft copolymers or the number of arms in star polymers, and thus the (co)polymer's macroscopic properties and application prospects.^3^ Importantly, the current implementation of the RDRP strategy operates almost exclusively with monofunctional initiation/transfer sites, allowing for a maximum of one polymeric chain per site. Unfortunately, this inherent limitation is often further exacerbated by the inefficiency of the reactions used for the initiation/transfer site attachment and by the decreased initiation efficiency (IE) observed in some Cu-RDRP systems.^21^ Collectively, these shortcomings impose significant limitations on the total number of polymeric chains that could be installed onto a given CPA precursor, which is detrimental in applications relying on high grafting density^14,22^ and generally represents a clear bottle-neck in macromolecular design.

Cu-RDRP can potentially provide an elegant solution to some of these drawbacks in the form of multifunctional initiation sites. In multifunctional Cu-RDRP initiators (*e.g.*, CCl_4_ or α-di/trichloro esters), more than one of the present carbon-halogen bonds can theoretically undergo activation by a copper catalyst, initiating the growth of multiple polymeric chains from a single carbon atom. In the case of CPA synthesis, bi- or trifunctional initiation sites could possibly be employed, providing instantaneous amplification of the functionality of the precursor-derived (macro)initiator. Interestingly, multifunctional initiators have been considered since the early days of Cu-RDRP but never achieved a widespread use.^23–25^ This can be ascribed to the uncertainty about the actual functionality of these compounds and also to the field's rapid adoption of active (monofunctional) brominated initiators, such as those containing the 2-bromoisobutyryl (BriB) group, that became the preferred choice in many Cu-RDRP scenarios, including CPA synthesis.^14,26–30^ A rare example of multifunctional Cu-RDRP initiator usage in CPA synthesis was the grafting from cellulose esters decorated with bifunctional dichloroacetate initiation sites reported by Vlček *et al.*^31,32^ However, while these pioneering efforts resulted in a comparatively higher grafting density, they suffered from two serious limitations. Firstly, the dichloroacetate initiator lacked universality, being successfully used for methacrylates only. More importantly, the grafting density was still seriously diminished by the typical inefficiency of the initiation site attachment that traditionally relies on the acylation of precursor's hydroxyl or amino groups with an α-haloacyl halide, whereby only relatively little initiator is often introduced despite using a large excess of the acylation reagent.^14,31,33,34^ While this issue is also relevant to small-molecule CPA precursors,^34^ it is particularly pronounced for macromolecular substrates such as cellulose where the supramolecular structure significantly diminishes the acylation efficiency,^14,30,33^ necessitating the development of elaborate, multi-step strategies to prepare densely-grafted products.^14^ Furthermore, the standard acylation protocols generate byproducts that need to be removed in a separate step *via* recrystallization/chromatography (for small molecules)^34–36^ or precipitation/extraction (for macromolecular precursors).^14,32,33,37^ In addition, long reaction times ranging from several hours up to a week are typically used in these transformations.^13,14,35,38^ Collectively, the described limitations have so far prevented the macromolecular community from exploiting the full potential of multifunctional Cu-RDRP initiating sites in CPA synthesis.

Clearly, several criteria have to be met in order to successfully amplify the (macro)initiator functionality through multifunctional Cu-RDRP initiation sites and enable thus the synthesis of CPAs with a severalfold higher number of polymeric chains as compared to current protocols. Firstly, the multifunctional initiation sites must be sufficiently universal, that is, applicable to different monomer classes, ideally under diverse polymerization conditions. Further, the IE should be sufficiently high with respect to both the entire (macro)initiator and the individual multifunctional initiation sites where activation of all the available carbon-halogen bonds should be feasible. Finally, the introduction of multifunctional initiation sites into CPA precursors must be considerably more efficient than with the contemporary acylation protocols.

**Scheme 1:**
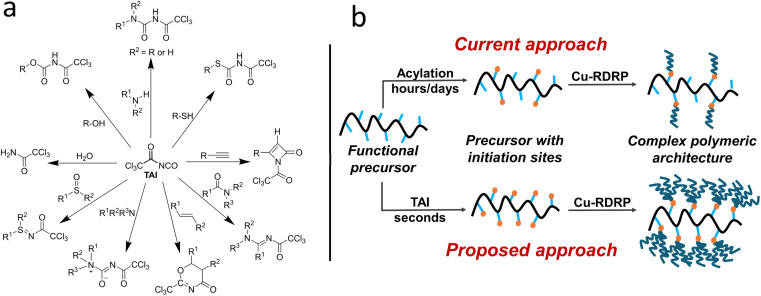
(a) Selected reactions of TAI deemed as relevant to polymer chemistry; (b) a scheme contrasting the number of chains installed onto a CPA precursor using the current and the newly proposed (TAI-based) approach.

Herein, we hypothesize that adducts of trichloroacetyl isocyanate (TAI) can potentially meet all these criteria. TAI is a commercially available *in situ* derivatizing reagent used in NMR spectroscopy to facilitate structural assignment of compounds bearing hydroxy,^39–42^ thio,^43^ and amino^40^ groups. In most cases, these moieties undergo rapid, quantitative, and uncomplicated 1,2-addition reactions with TAI, affording carbamate, thiocarbamate, and urea derivatives, respectively. The TAI method is also routinely applied for end-groups analysis in polymer chemistry.^44–48^ Besides 1,2-additions, TAI can take part in other reactions, which increases the diversity of CPA precursors it can modify (Scheme 1a).^39,49^ We propose here that TAI can be repurposed as a highly efficient vehicle for installing trichloroacetyl groups (TAGs) onto a variety of small-molecule- and macromolecular CPA precursors, avoiding the limitations of the traditional acylation approach. Importantly, several studies used TAG-bearing compounds as initiators for transition metal-catalyzed RDRP of different monomers,^23,24,50–54^ and there is limited evidence that TAGs can act as bi- or trifunctional initiators.^23,53^ Additionally, the utility of halo-isocyanates as initiating site precursors in the block and graft copolymer synthesis *via* free-radical polymerization has been recognized already in 1980s by Bamford and coworkers.^55–59^ As visualized in Scheme 1b, the unique combination of TAI reactivity and TAG multifunctionality could effectively amplify the CPA (macro)initiator functionality and provide thus an access to a whole new group of CPAs characterized by dramatically increased chain density and, potentially, new properties and applications.

In this study, we strived to firmly establish the TAI-based Cu-RDRP strategy as a powerful yet simple tool in CPA synthesis. To this end, we investigated and confirmed the considerable universality of TAI-derived initiators by identifying the polymerization conditions under which well-controlled Cu-RDRP of different monomer classes can be achieved. Subsequently, we used ^1^H NMR spectroscopy and triple-detection size-exclusion chromatography (TD-SEC) to prove conclusively that the TAI-derived TAGs act as inherently trifunctional initiators, which has a profound impact on the topology of the attained polymeric architectures and distinguishes the TAI-based strategy from earlier RDRP approaches. Finally, we provide examples documenting the strong points of the new strategy in various relevant scenarios such as the (one-pot) synthesis of star-shaped and branched CPAs, including a novel “star-on-star” graft copolymer topology, and the modification of otherwise problematic cellulose substrates yielding ultra-high-MW ultra-dense bottle-brush copolymers and diverse surface-grafted “2D” and 3D objects with unprecedented ease.

## Results and discussion

### Developing conditions for Cu-RDRP initiated by TAI adducts

**Scheme 2:**
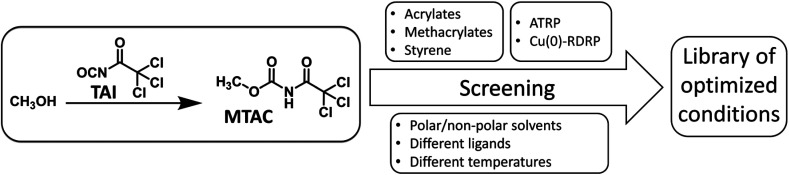
The workflow of the polymerization conditions screening.

In order to probe the universality of TAI-derived initiators, we conducted an extensive screening of multiple polymerization parameters, seeking conditions under which well-controlled Cu-RDRP, characterized by low dispersity and pre-determined MWs of products, can be achieved for monomers from different classes: styrene, acrylates, and methacrylates (Scheme 2).

In the optimization study, we used methyl acrylate (MA), methyl methacrylate (MMA), and styrene as model monomers together with a model initiator, methyl *N*-trichloroacetyl carbamate (MTAC), that was readily obtained by the addition of TAI into dry methanol, followed by the evaporation of the methanol excess (Scheme 2 and S1†). We investigated two Cu-RDRP approaches, namely (conventional) atom transfer radical polymerization (ATRP)^26,60^ and Cu(0)-mediated RDRP [Cu(0)-RDRP],^61^ employing Cu(i) salts (CuBr or CuCl) and Cu(0) (activated copper wire) as catalysts, respectively. Note that Cu(0)-RDRP is sometimes denoted as single-electron transfer living radical polymerization (SET-LRP)^62,63^ or supplemental activation reducing agent (SARA) ATRP^64,65^ with reference to the expected polymerization mechanism; since we do not address the mechanism in this study, we opted for the generic term Cu(0)-RDRP. Me_6_TREN and PMDETA were used as ligands at different ligand/initiator ratios. Solvents of different polarity were tested to enable future application of the developed strategy to CPA precursors of different solubility. Temperatures ranging from r.t. to 110 °C were utilized depending on the targeted monomer. The monomer/initiator (*M*/*I*) ratio of 200 : 1 was used in optimization runs, with other *M*/*I* ratios subsequently employed under selected conditions. MW and dispersity values were obtained through size-exclusion chromatography (SEC) calibrated with appropriate standards.

In Table 1, we summarize the selected optimized polymerization conditions for performing MTAC-initiated Cu-RDRP of the model monomers, selected mainly on the basis of achieving high monomer conversion, low dispersity, and a reasonably good match between theoretical and experimental MWs. Numerous additional experimental conditions tested during the extensive screening process are then collected in ESI (Tables S1–S3†) and might be of use in specific cases, *e.g.*, when a particular ligand/solvent combination is desired.

**Table 1:** Selected optimized conditions for MTAC-initiated Cu-RDRP of model monomers^a^

Entry	Mon.	Cat.	Solvent	Ligand (eq.)	*M*/*I*	*T* (°C)	Time (h)	Conv.^b^ (%)	*M*_n_ (theor.)^c^	*M*_n_ (SEC)^d^	*Đ* d
1	MA	Cu(0)	DMSO	PMDETA (0.2)	200	60	4	96	16 500	22 200	1.19
2	MA	Cu(0)	DMAc	PMDETA (0.2)	200	60	24	98	17 000	21 100	1.27
3	MA	Cu(0)	DMSO	Me_6_TREN (0.5)	200	60	5	91	15 700	21 800	1.17
4	MA	Cu(0)	DMSO	Me_6_TREN (0.5)	200	r.t.	24	95	16 400	20 400	1.12
5	MA	Cu(0)	Dioxane	Me_6_TREN (0.5)	200	60	24	99	17 000	22 000	1.19
6	MA	Cu(0)	Toluene	Me_6_TREN (0.5)	200	60	24	89	15 300	18 600	1.19
7	MA	Cu(0)	DMSO	Me_6_TREN (0.5)	50	60	5	80	3700	4900	1.22
8	MA	Cu(0)	DMSO	Me_6_TREN (0.5)	100	60	5	89	7800	10 500	1.18
9	MA	Cu(0)	DMSO	Me_6_TREN (0.5)	400	60	7	97	33 500	48 200	1.20
10	MMA	Cu(0)	DMSO	PMDETA (0.2)	200	85	3	87	17 600	24 700	1.27
11	MMA	Cu(0)	DMSO	Me_6_TREN (0.2)	200	85	4	85	17 200	23 000	1.20
12	MMA	Cu(0)	DMSO	Me_6_TREN (0.2)	200	r.t.	24	91	18 400	25 900	1.19
13	MMA	Cu(0)	Dioxane	PMDETA (1.0)	200	85	5	90	18 200	26 600	1.16
14	MMA	Cu(0)	Toluene	PMDETA (1.0)	200	85	24	95	19 200	19 600	1.12
15	MMA	Cu(0)	DMSO	Me_6_TREN (0.2)	50	85	4	>99	5200	5900	1.27
16	MMA	Cu(0)	DMSO	Me_6_TREN (0.2)	100	85	4	90	9200	10 900	1.25
17	MMA	Cu(0)	DMSO	Me_6_TREN (0.2)	400	85	7	93	37 400	43 700	1.20
18	MMA	Cu(0)	Toluene	PMDETA (1.0)	50	85	18	>99	5200	5100	1.26
19	MMA	Cu(0)	Toluene	PMDETA (1.0)	100	85	18	96	9800	10 800	1.22
20	MMA	Cu(0)	Toluene	PMDETA (1.0)	400	85	45	92	37 000	33 000	1.12
21	MMA	CuBr	Toluene	PMDETA (1.0)	200	85	22	90	18 200	15 500	1.12
22	MMA	CuBr	Dioxane	PMDETA (1.0)	200	85	24	88	17 800	18 200	1.09
23	MMA	CuBr	Dioxane	PMDETA (1.0)	50	85	18	>99	5200	5000	1.21
24	MMA	CuBr	Dioxane	PMDETA (1.0)	100	85	18	85	8700	9500	1.13
25	MMA	CuBr	Dioxane	PMDETA (1.0)	400	85	45	70	28 200	29 700	1.11
26	Styrene	Cu(0)	Toluene	Me_6_TREN (0.2)	200	90	24	45	9700	10 400	1.19
27	Styrene	CuBr	—	Me_6_TREN (1.0)	400	110	21	95	40 000	45 300	1.25
28^e^	Styrene	CuBr	—	Me_6_TREN (1.2)	50	110	2	89	4900	6000	1.30
29	Styrene	CuBr	—	Me_6_TREN (1.0)	100	110	6	84	9000	10 500	1.26
30	Styrene	CuBr	—	Me_6_TREN (1.0)	200	110	6	86	18 100	21 100	1.21
31	Styrene	CuBr	—	Me_6_TREN (1.0)	800	110	24	92	77 000	75 200	1.34

a Standard polymerization conditions: MTAC initiator; catalyst (cat.): 10 cm of activated copper wire in Cu(0)-RDRP, CuBr (1 eq.) in ATRP; ligands: tris[2-(dimethylamino)ethyl]amine (Me_6_TREN) and *N*,*N*,*N*′,*N*′′,*N*′′-pentamethyldiethylenetriamine (PMDETA); solvent/monomer (Mon.) = 1 : 1 (v/v).

b Monomer conversion determined by ^1^H NMR (for MA, Fig. S2, ESI) or gravimetrically (for MMA and styrene).

c Theoretical *M*_n_ calculated from the *M*/*I* ratio and conversion, assuming 100% IE.

d Determined by SEC with poly(MMA) calibration (for MA and MMA) or polystyrene calibration (for styrene).

e CuBr_2_ (0.2 eq.) was added as a deactivator, and the concentration of ligand was increased to account for this addition.

**Fig. 1:**
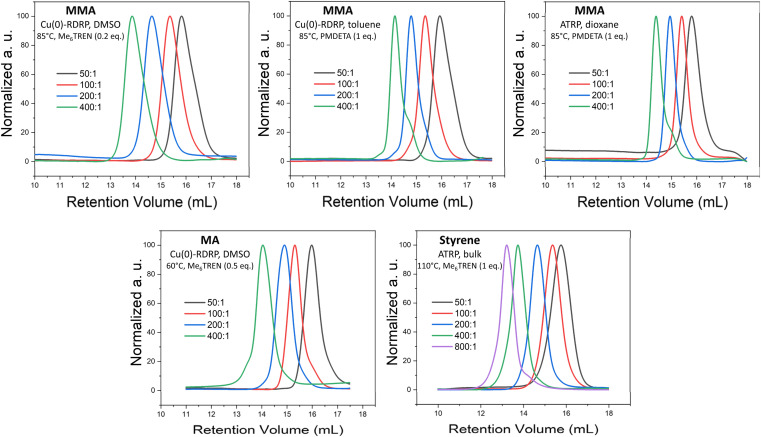
SEC elugrams of selected polymers prepared by MTAC-initiated Cu-RDRP at different *M*/*I* ratios. Product characteristics are provided in Table 1. The noticeable low-MW shoulder in the SEC elugrams of the polymers synthesized at the highest *M*/*I* ratios (400 : 1 or 800 : 1) in non-polar solvents/bulk are ascribed to the products of early termination or competing transfer reactions that tend to be more pronounced when aiming for high-MW products.^66^

Our screening showed that MTAC-initiated ATRP (CuBr or CuCl as a catalyst) of MA was largely unsuccessful. Under host of different polymerization conditions, including different solvents, ligands, and temperatures, no polymerization was observed, or the achieved conversions were very low (entries 1–15, Table S1†). On the other hand, Cu(0)-RDRP catalyzed by Cu wire yielded low-dispersity polymers at high conversion under a range of polymerization conditions, including both polar and non-polar solvents (entries 1–9, Table 1; additional experiments in Table S1†). SEC elugrams of obtained polymers are provided in Fig. 1 and S3;† a kinetic experiment documenting the good polymerization control is shown in Fig. S4.†

Further, we demonstrated that the MTAC initiator works remarkably well for MMA, affording high conversions and low-dispersity products under a range of conditions, including both ATRP and Cu(0)-RDRP methods, different temperatures and solvents of different polarity (entries 10–25, Tables 1 and S2;† for SEC elugrams see Fig. 1 and S5†). A well-controlled character of the polymerization under the developed conditions was confirmed by kinetic experiments (Fig. S6–S8†). The high chain-end fidelity of poly(MMA) prepared *via* MTAC-initiated ATRP in dioxane was demonstrated by chain-extension experiments. To this end, poly(MMA) prepared at high conversion (*M*_n_ = 9 500, *Đ* = 1.13; entry 24, Table 1) was successfully used as a macroinitiator to initiate chain-extension with MMA and block-copolymerization with styrene, which is visualized by clear shifts of the corresponding SEC elugrams and the significant increases in MWs (Fig. S9†).

Finally, styrene was polymerized at 90 °C through a well-controlled Cu(0)-RDRP (in DMSO and toluene) and ATRP (in toluene); however, the process was rather slow (*ca.* 50% conversions reached). High conversions were achieved *via* ATRP in bulk at 110 °C (entries 26–31, Tables 1 and S3† for SEC traces see Fig. 1 and S10;† for kinetics see Fig. S11†). It is of note that Cu(0)-RDRP of styrene in DMSO and DMAc was plagued by gel formation on the copper wire. Such gel formation has been described previously for other Cu(0)-RDRP systems.^62,67,68^ Collectively, it is rather remarkable that MTAC is able to initiate well-controlled Cu(0)-RDRP of all three studied model monomers in toluene or dioxane because reports on successful Cu(0)-RDRP in non-polar solvents are extremely rare in literature.^66,69,70^

Next, to verify that the Cu-RDRP conditions established using MTAC are useable also for TAI adducts with other functional groups, we synthesized *N*,*N*-diisopropylamine/TAI adduct, 1,1-diisopropyl-3-(2,2,2-trichloroacetyl)-urea (DTAU) (Fig. S12†). DTAU-initiated Cu-RDRP of styrene, MMA, and MA was then performed under the optimized conditions from our library. The experimental results together with the corresponding SEC traces, collected in Fig. S13,† prove that very similar polymers are obtained irrespective of the linker connecting the initiating TAG fragment to the CPA precursor. This finding suggests that the developed library of Cu-RDRP conditions will be applicable to a variety of CPA precursors bearing TAI-reactive functions.

Having successfully identified polymerization protocols for model monomers, we next sought to investigate the universality of the developed conditions with respect to other monomers from the same class, including some important functional variants.^71–73^ To this end, we applied selected conditions to other (meth)acrylates, including functional ones (Table 2 and Fig. S14†). Although some of the (meth)acrylate analogues (expectedly) did not behave identically as the model monomers, we could easily identify conditions in our library providing well-defined products, which highlights the utility of the extensive screening approach we employed in this study. For example, butyl acrylate (BA) polymerized poorly in toluene (entry 1, Table 2) while quickly affording a well-defined product at quantitative conversion in a bi-phasic system^35^ in DMSO (entry 2, Table 2). Similarly, Cu(0)-RDRP of 2-hydroxyethyl methacrylate (HEMA) was uncontrolled in DMSO while affording a well-defined product in dioxane^75^ (*cf.* entries 7 and 8, Table 2). Further, for 2-hydroxyethyl acrylate (HEA), we obtained a well-defined polymer by using a lower ligand loading (*cf.* entries 3 and 4, Table 2). On the other hand, conditions originally developed for MMA could be directly applied to butyl methacrylate (BMA) and glycidyl methacrylate (GMA) without any changes (entries 5 and 6, Table 2). Taken together, the results confirm the considerable universality of TAI-derived initiators and manifest that our library of optimized conditions (Table 1) can serve as an excellent starting point when polymerizing other (meth)acrylates.

**Table 2:** MTAC-initiated Cu-RDRP of other (meth)acrylates^a^

Entry	Monomer	Solvent	Ligand (eq.)	*T* (°C)	Time (h)	Conv.^b^ (%)	*M*_n_ (theor.)^c^	*M*_n_ (SEC)^d^	*Đ* d
1	BA	Toluene	Me_6_TREN (0.5)	60	24	16	4200	4600	1.45
2^e^	BA	DMSO	Me_6_TREN (0.5)	60	7	97	25 100	35 100	1.25
3	HEA	DMSO	Me_6_TREN (0.5)	60	8	50	11 800	46 500	1.37
4	HEA	DMSO	Me_6_TREN (0.2)	60	24	42	10 000	15 000	1.18
5	BMA	Toluene	PMDETA (1.0)	85	24	84	24 000	20 400	1.15
6	GMA	DMSO	Me_6_TREN (0.2)	85	2	80	23 000	21 400	1.23
7^f^	HEMA	DMSO	Me_6_TREN (0.2)	85	24	99	13 100	20 000	1.85
8^f^	HEMA	Dioxane	PMDETA (1.0)	85	1	99	13 100	17 200	1.28

a Standard polymerization conditions: MTAC initiator, *M*/*I* = 200 : 1, 10 cm of activated copper wire, monomer/solvent = 1 : 1 (v/v).

b Monomer conversion determined by ^1^H NMR.

c Theoretical *M*_n_ calculated from the *M*/*I* ratio and conversion, assuming 100% IE.

d Determined by SEC with poly(MMA) calibration [directly (BA, BMA, GMA) or after acetylation^74^ (HEMA)] or by TD-SEC (HEA).

e Biphasic polymerization mixture.

f *M*/*I* = 100 : 1 and 5 cm of activated copper wire were used.

To complement the results on TAG-initiated Cu-RDRP, we also performed a preliminary investigation into the hydrolytic stability of the TAI-derived carbamate linker present in most (macro)initiators used in this study. It can be expected that different TAI-derived linkers, connecting the initiating TAGs with the derivatized precursor, may show different hydrolytic stability/pH sensitivity. We envisage that the properties of these linkers could be potentially exploited in fields such as drug delivery where, for example, the use of CPAs featuring a pH-sensitive carbamate linker has already been established.^76–78^ Nevertheless, the situation can be rather complex as organic carbamates show very varied hydrolytic stability depending on their structure and experimental conditions.^79–82^ To get a preliminary insight, we studied hydrolytic stability of in-chain carbamate linkers in a poly(HEA) star polymer. As shown in Fig. S15† and in the accompanying discussion, the carbamate linker showed to be considerably resistant to hydrolysis in a wide pH range.

### Functionality of TAI-based initiation groups

The functionality of TAG(s) introduced into CPA precursors by the reaction with TAI represents a key parameter defining the final polymeric architecture and distinguishes the TAI-based strategy from previous approaches based on monofunctional initiation sites such as BriB. Surprisingly, the functionality of TAG-containing Cu-RDRP initiators has been addressed only rarely in literature. In their seminal paper, Destarac *et al.* concluded based on NMR data that the studied methyl trichloroacetate acts as – at least – a bifunctional initiator in ATRP of styrene.^23^ Additionally, Lorandi *et al.* have recently reported that trichloroacetic acid behaves as a trifunctional initiator in ATRP of acrylic acid,^53^ maintaining that, upon initiation, the remaining chlorine(s) of the original TAG are increasingly prone to activation (and, subsequently, initiation) due to the penultimate effect.^83^ Considering these limited previous results, we decided to perform an in-depth investigation into the functionality of the TAI-derived TAGs under our developed polymerization conditions.

First, we used ^1^H NMR spectroscopy to evaluate the initiator functionality for model low-MW poly(MA), poly(MMA), and polystyrene prepared by MTAC-initiated Cu-RDRP. In the respective spectra, we identified the characteristic signals of the initiator fragment (the –OCH_3_ group) and the terminal (chlorine-bearing) and in-chain monomeric units. We then used the relative intensities of these signals, together with the polymer *M*_n_ value determined by SEC, to calculate initiator functionality, obtaining values close to 3 in all cases (for details see Fig. S17–S19† and the accompanying discussion). It is of note that the used poly(MMA) sample was obtained at quantitative conversion (entry 23, Table 1), confirming the high end-chain fidelity attained under the used conditions. Overall, our findings suggest that the MTAC-initiated polymers have the topology of three-arm stars. Consequently, our reported MW values obtained by SEC with relative calibration are slightly underestimated due to the smaller hydrodynamic volume of branched polymers. Additionally, the poly(MMA)-*b*-polystyrene synthesized above in the chain-extension experiment (Fig. S9†) should be considered as a 3-arm star with diblock arms.

Next, we wanted to verify that the trifunctionality of TAI-derived TAGs is retained also for high-MW CPAs (*i.e.* a real-world scenario). Since high-MW polymers are not amenable to the simple end-group analysis applied above, we selected a different approach based on the viscometric analysis of the initiation site-related branching using TD-SEC. We reasoned that a standalone TAG provides branching only if the initiator acts as trifunctional while its mono- and bifunctionality leads to a linear polymer.

**Fig. 2:**
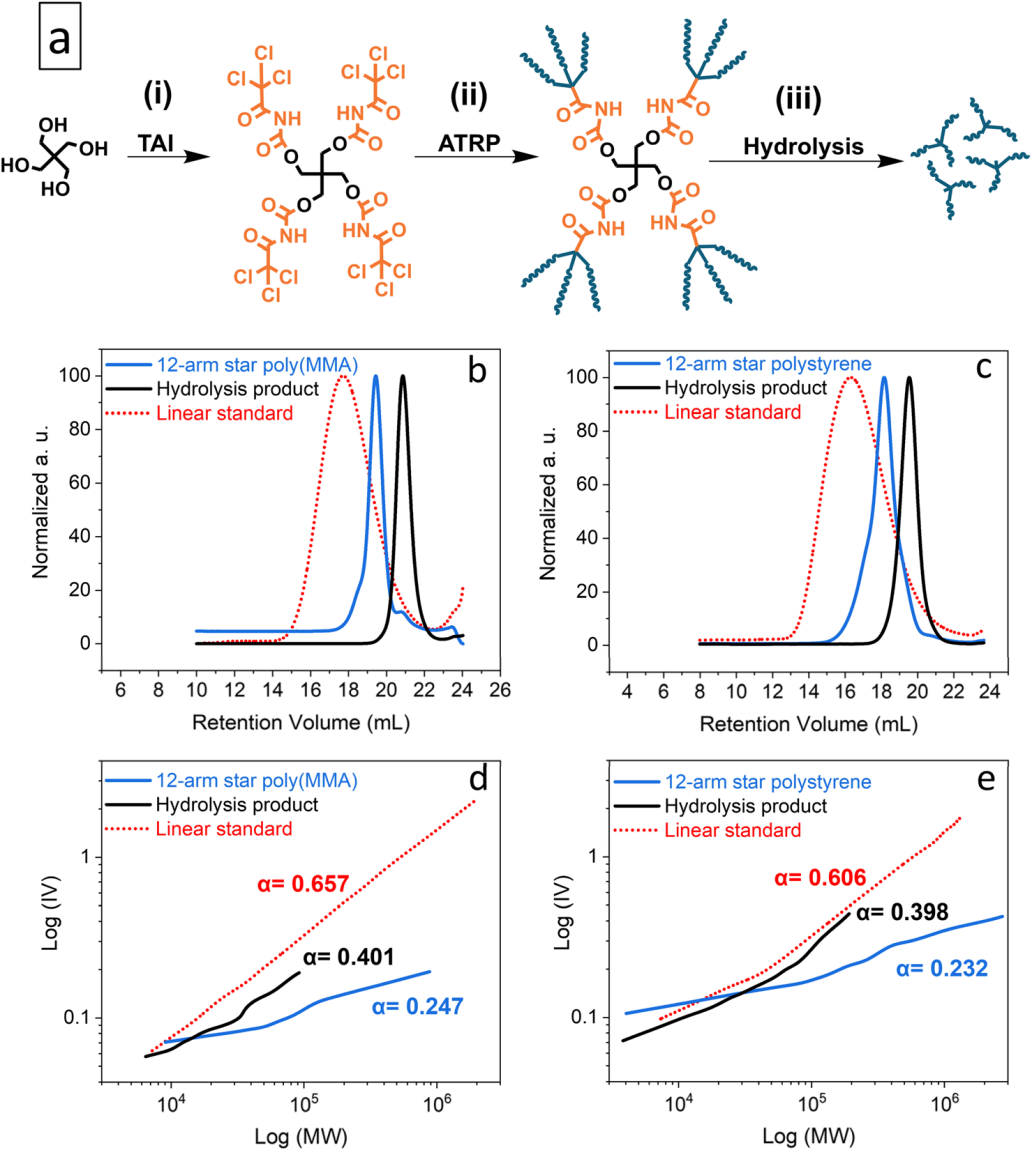
TAG functionality study: general scheme of the synthesis of model multi-arm stars based on a pentaerythritol core (a); elugrams – RI traces (b and c) and M–H plots (d and e) from the TD-SEC analysis of the synthesized poly(MMA) (b and d) and polystyrene (c and e) multi-arm star polymers and of products of their alkaline hydrolysis. Data for broad linear poly(MMA) and polystyrene standards are shown for comparison. See Table S4† for experimental conditions and results.

As model CPAs, we prepared star-shaped poly(MMA) and polystyrene *via* Cu-RDRP initiated by the pentaerythritol/TAI adduct, pentaerythritol tetrakis((2,2,2-trichloroacetyl) carbamate) (PTAC) (Fig. 2 and S20†). Using TD-SEC, we then analyzed the parent star polymers as well as the individual TAG-initiated polymeric segments released from the pentaerythritol core *via* alkaline hydrolysis^33^ of carbamate linkers (Fig. 2). As seen from the data summarized in Table S4,† the poly(MMA) star showed low dispersity of 1.21, with the SEC elugram (Fig. 2b) featuring only a small high-MW shoulder, indicating negligible extent of star–star coupling despite the high monomer conversion of 92%. On the other hand, the polystyrene variant was comparatively less well-defined (*Đ* = 1.69), probably due to the presence of both the coupling products and free segments as suggested by the SEC elugram shape (Fig. 2c). Nevertheless, the low dispersity of the hydrolytically released star segments/arms indicated that well-controlled polymerization was achieved for both monomers.

Fig. 2d and e shows Mark–Houwink (M–H) plots for both the parent multi-arm star polymers and the hydrolytically released segments, alongside the data for broad linear poly(MMA) and polystyrene standards. In addition, the determined M–H *α* constants, which provide a good measure of polymer branching, are also displayed. While the broad linear standards provided the expected *α* ≈ 0.6, the considerably lower *α* value of approximately 0.4 obtained for the released segments confirmed branched character of these polymers and, thus, the TAG trifunctionality.^84^ Finally, the *α* values for the parent polymers, presumably 12-arm stars, are even lower (≈0.2), as expected for the comparatively denser polymeric architecture.^84^

Note that we performed also PTAC-initiated polymerization of MA (Table S4†); however, we were unable to cleanly release the individual segments using our alkaline hydrolysis method in this case. Therefore, in Fig. S21,† we provide only the TD-SEC analysis of the parent star polymer together with the comparison data for a broad linear poly(MA)standard. The same *α* constant as for the poly(MMA) star above (≈0.25) was obtained from the M–H analysis indicating a similar number of star arms and hence TAG trifunctionality also in this case.

### Applications of the TAI-based strategy

Having successfully established that TAI functions as an efficient vehicle for introducing universal multifunctional initiation sites into different precursors, we highlight in this section some of the advantages that this new strategy brings to CPA synthesis.

**Fig. 3:**
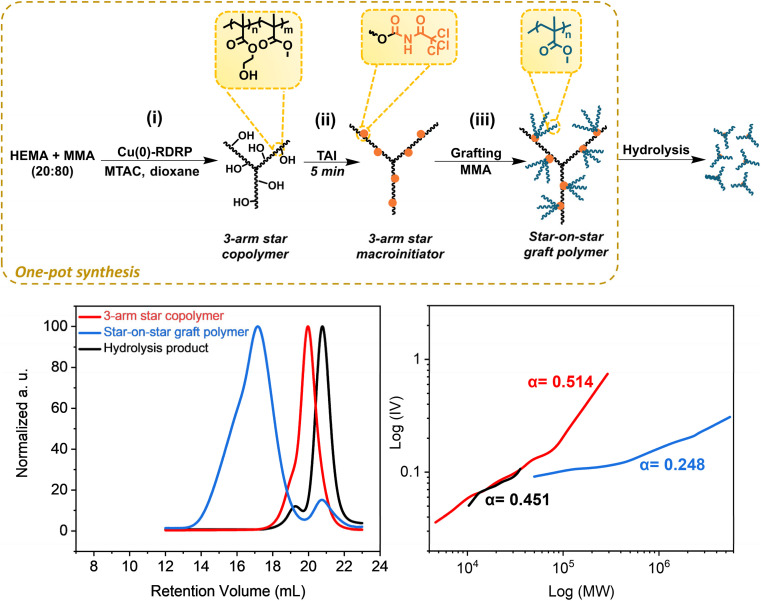
*De novo* one-pot synthesis of the poly(HEMA-*co*-MMA)-*graft*-poly(MMA) hybrid star/graft copolymer of “star-on-star” topology. (Top) General reaction scheme; for experimental conditions see Table S5.† (Bottom) TD-SEC analysis of products at individual stages – elugrams (left) and M–H plots (right).

First, we show that the strategy allows for the clean *in situ* introduction of initiation sites in multi-step protocols without intermediate isolation, which enables the one-pot *de novo* synthesis of graft copolymers that avoids the isolation/purification steps typical for standard approaches.^14,32,33^ To this end, we conducted a three-step protocol depicted in Fig. 3; for experimental details and results see Table S5.† First, we performed a MTAC-initiated copolymerization of HEMA and MMA (20/80 mol%) by Cu(0)-RDRP in dioxane, yielding a well-defined poly(HEMA-*co*-MMA) copolymer (*M*_n_ = 23 400, *Đ* = 1.23) at quantitative conversion (Fig. S22,† top). Subsequently, we *in situ* modified part of the pendent hydroxyl groups in HEMA units by adding TAI (Fig. S22,† bottom). Finally, upon the addition of another batch of MMA and solvent, we continued the polymerization to yield the final graft copolymer (Fig. S23†). Owing to the TAG trifunctionality, the copolymer involves three-arm stars grafted from a three-arm star backbone, *i.e.*, “star-on-star” architecture – apparently a novel type of CPA that structurally represents a hybrid between a star and a graft copolymer. The inflated dispersity of the final product (1.95) is mainly ascribed to the recombination reactions at the macroinitiator preparation stage where quantitative conversion was targeted (a high-MW shoulder in the SEC elugram of the macroinitiator supports this assumption). Nevertheless, TD-SEC analysis showed that the poly(MMA) grafts, removed by alkaline hydrolysis,^33^ were extremely well defined (*Đ* = 1.05), indicating a high degree of polymerization control in the grafting step. Note that there is a small lower-MW signal in the SEC chromatogram of the star-on-star copolymer. This signal is ascribed to the polymer initiated by the products of TAI reaction with present impurities (*e.g.*, water). The M–H plots provided in Fig. 3 showed *α* values consistent with the expected topology of three-arm stars (for the macroinitiator precursor and cleaved grafts) and with the highly branched final star-on-star copolymer. Collectively, these results illustrate well that the TAI strategy opens avenues for unconventional approaches to the synthesis of graft and hyper-branched (co)polymers and enables designing of new CPA topologies.

**Fig. 4:**
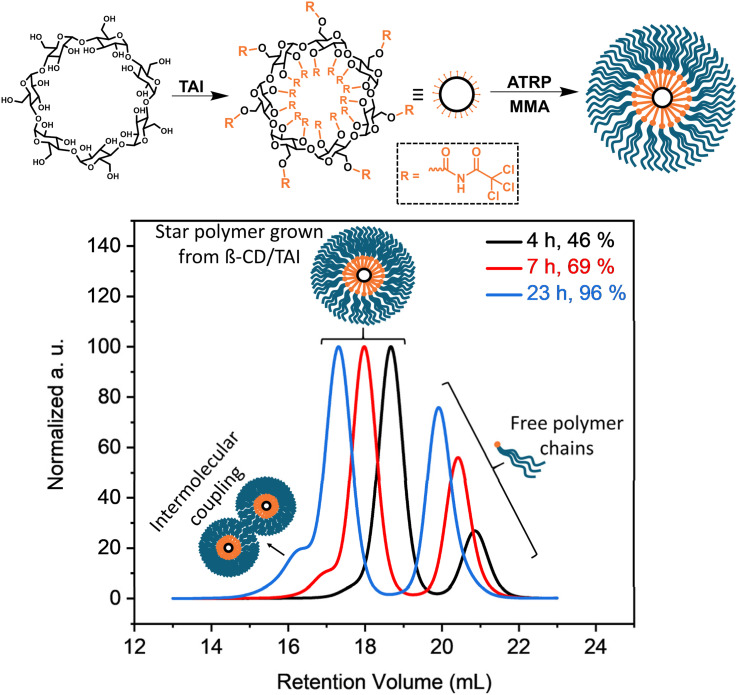
Synthesis of multi-arm poly(MMA) stars through ATRP initiated by the β-CD/TAI adduct. (Top) General reaction scheme; (bottom) TD-SEC analysis (elugrams – RI traces) of samples taken at different polymerization stages. Experimental details are provided in Table S6.†

In order to highlight the utility of the TAI-based initiator functionality amplification strategy in the synthesis of previously inaccessible multi-arm star-shaped polymers, we conducted polymerization of MMA initiated by a β-cyclodextrin (β-CD)/TAI adduct (Fig. 4). The adduct was prepared by the reaction of pre-dried β-CD with an excess of TAI whereby the unreacted TAI was quenched with DMSO. The ^1^H NMR spectrum of the reaction mixture (Fig. S24†) confirms the full modification of the β-CD hydroxyl groups as well as the presence of the DMSO/TAI adduct and trichloroacetamide originating from TAI reaction with residual water. The latter two compounds served as low-MW sacrificial initiators.^26,33,85^ The SEC elugrams of the starting β-CD and the β-CD/TAI adduct displayed in Fig. S25a† show a clear shift of the sharp β-CD peak to higher MWs upon TAI modification. Afterward, the (macro)initiator solution was used to initiate ATRP of MMA in dioxane. Finally, the arms of the isolated star polymer were removed *via* alkaline hydrolysis for further analysis.

The data shown in Fig. 4, S25b and Table S6† confirmed that the use of a sacrificial initiator represents an efficient strategy^33^ for suppressing the formation of intermolecular coupling products (visible as high-MW shoulders in SEC elugrams) even at the almost quantitative monomer conversion reached here. Both the star polymers and cleaved arms/free-growing chains were exceptionally well-defined throughout the polymerization course (*Đ* = 1.15 and 1.05, respectively, at 96% conversion). Additionally, there was an excellent match in *M*_n_ and *Đ* values evaluated for the free-growing chains (the low-MW signal in the SEC of the isolated products) and the mixture of the free-growing chains and the star arms obtained after hydrolysis (Table S6 and Fig. S25b†), proving that both the star arms and free chains grew at a similar rate. At the same time, the determined *M*_n_ values were considerably higher than the theoretical ones calculated from conversion and the MMA/TAI ratio. Collectively, these observations suggest that a part of the TAGs on the β-CD/TAI adduct did not initiate polymerization due to the extreme steric crowding at the TAI-modified β-CD while the remaining TAGs acted as trifunctional initiators, owing to the increased reactivity of the chlorine atoms remaining at TAGs that underwent initiation.^53^ Nevertheless, a simple comparison of the *M*_n_ values obtained for the final multi-arm star polymer and for the arms released therefrom suggests that one β-CD core bears approximately 15 poly(MMA) segments that actually are 3-arm stars on their own. Therefore, the product can be considered as a 45-arm star polymer, highlighting the clear advantage of the new strategy over the previous approaches based on monofunctional initiators that yield, at best, 21 arms from the same precursor in a much more laborious process.^34,37^

Next, we presumed that the high TAI reactivity will make the new strategy particularly useful in the synthesis of CPAs based on difficult-to-modify substrates. Herein, this is exemplified by the modification of cellulose that has been previously shown to be resistant to the introduction of high concentrations of Cu-RDRP initiation sites using standard acylation protocols.^14,33^ First, we studied the reactivity of cellulose (microcrystalline AVICEL PH-101) toward TAI in different solvents. We found that cellulose, dissolved in the traditional cellulose solvent DMAc/LiCl,^86^ could be easily fully modified with a slight excess of TAI (4 eq. toward the anhydroglucose units of cellulose) as documented by the ^1^H and ^13^C NMR spectra of the isolated adduct (Fig. S26†). Furthermore, overnight stirring of dioxane-activated cellulose^86^ in dioxane containing 4 eq. of TAI led to complete cellulose modification and dissolution. Similarly, the dioxane-activated cellulose afforded a clear solution of the cellulose/TAI adduct after 2 h of reaction with 6 eq. of TAI in THF. Moreover, we found that pre-dried, non-activated cellulose could be fully modified and dissolved when reacted with TAI (6 eq.) in acetonitrile for 4 days. Most importantly, we also revealed that cellulose becomes highly reactive toward TAI when the modification is conducted in DMSO, *i.e.*, a solvent that strongly swells cellulose and increases its accessibility and reactivity.^87^ When 5 eq. of TAI were added to a suspension of non-dried (or pre-dried and soaked in DMSO overnight) cellulose in DMSO, a clear solution was obtained within 1 min. This finding is remarkable considering the reactivity of TAI toward DMSO and confirms that the modification of substrates in TAI-reactive solvents (*e.g.*, DMSO or DMAc), as proposed by Samek *et al.*,^39^ is possible also for heterogeneous reactions with polymeric substrates. While we did not focus here on testing the universality of this modification protocol with respect to different cellulose types, we can confirm that the same rapid modification in DMSO was obtained also for a considerably higher-MW cellulose Sigmacell type 101. We thus envisage that this protocol may find important applications in the field of cellulose characterization where a similar but considerably more laborious approach based on cellulose modification with phenyl isocyanate is used for cellulose MW determination by SEC.^88^

**Fig. 5:**
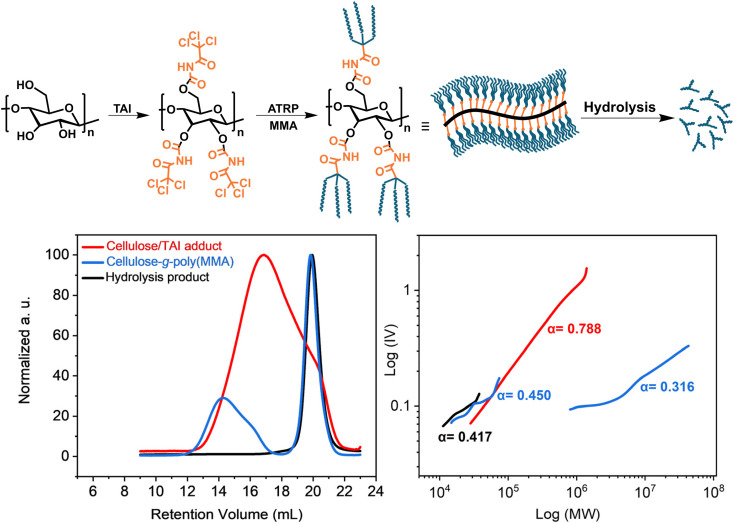
Synthesis of the ultra-dense bottle-brush cellulose-*g*-poly(MMA) graft copolymer *via* ATRP of MMA initiated by the cellulose/TAI adduct. (Top) General reaction scheme; (bottom) TD-SEC analysis (left – RI elugrams; right – M–H plots) of the cellulose/TAI macroinitiator, the copolymer obtained after 5 h, and poly(MMA) obtained after alkaline hydrolysis of the isolated product. Experimental details are provided in Table S7.†

Cellulose fully modified with TAI represents a unique macroinitiator that can potentially give rise to 9 polymeric chains per one backbone repeat unit, affording, upon graft copolymerization, extremely dense bottle-brush copolymers. To investigate this option, we synthesized a cellulose-*graft*-poly(MMA) copolymer *via* ATRP initiated by a cellulose/TAI adduct (Fig. 5). We first prepared a stock solution containing the cellulose/TAI adduct and MTAC as a low-MW sacrificial initiator by reacting cellulose (AVICEL) with 6 eq. of TAI in acetonitrile and subsequently quenching the excess of TAI by methanol. The TD-SEC analysis of the adduct revealed *M*_n_ of 106 700 and dispersity of 2.17 consistent with the characteristics of the cellulose precursor (Fig. 5).^86^ Subsequently, we used the obtained (macro)initiator solution to initiate ATRP of MMA in dioxane. As can be seen from the experimental data collected in Table S7,† 27% conversion was reached in 5 h, which corresponds to the *M*_n_(theor.) of 9 644 000, as calculated from the macroinitiator number-average degree of polymerization (DP_*n*_) of 147, assuming three TAI-modified hydroxyl groups per a repeat unit that initiate polymerization. After 24 h, 72% conversion was attained, corresponding to *M*_n_(theor.) of 25 539 000. In this context, it is rather remarkable how the application of a sacrificial initiator effectively suppresses intermolecular crosslinking reactions even for such an ultra-dense bottle-brush at very high monomer conversion.^33^

It is known that SEC of high-MW bottle-brushes is challenging due to the non-SEC elution behavior of high-MW fractions.^16,89^ Indeed, we observed delayed elution of high-MW polymer fraction(s), which obscured the MW analysis (for details see Fig. S27† and the accompanying discussion). Nevertheless, for the 5 h sample, we were able to obtain, using universal calibration, rather realistic *M*_n_ of 28 300 for the free-growing chains initiated by the sacrificial initiator (Table S7†). This value agreed well with that for the mixture of grafts and free-growing chains acquired through the alkaline hydrolysis of the isolated product (*M*_n_ = 24 400), confirming that the polymer grew at a similar rate from both the cellulose backbone-attached and free initiation sites. The close match between the experimental *M*_n_ values and the *M*_n_(theor.), calculated based on the monomer conversion and the MMA/TAG ratio (considering all forms of TAI adducts), indicates that the much lower than theoretical *M*_n_ of the graft copolymer determined by TD-SEC (3 174 000) is severely underestimated due to the effects discussed above. The high compactness of the prepared bottle-brush copolymer is well-illustrated by the low *α* constant obtained from the M–H plot (Fig. 5). Further, even though we were unable to obtain any MW values from the TD-SEC analysis of the 24 h sample, we note that a good match between the *M*_n_(theor.) and *M*_n_(SEC) values of the hydrolysis product was retained also in this case (Table S7†). Additionally, the unimodal character of the SEC signals (data not shown) together with the low obtained *Đ* of 1.11 suggested that the *M*_n_ of grafts was similar as that determined for the hydrolysate. Altogether, the obtained data point to the extreme MW of the final cellulose-*graft*-poly(MMA) copolymer despite the rather low-MW cellulose backbone employed. We predict that truly giant cellulose-based graft copolymers with MWs in the order of hundreds of millions should be readily accessible using this strategy when starting from regular cellulose substrates having MWs in hundreds of thousands.

**Fig. 6:**
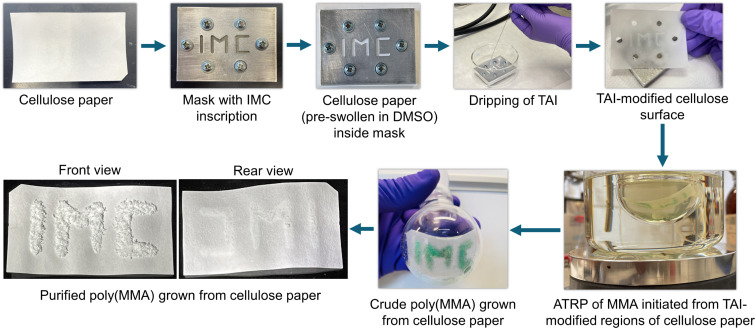
Spatial control in the modification of Whatman filter paper with TAI and subsequent ATRP SI grafting of MMA from the modified cellulose surface.

In the last part of this study, we highlight that the use of TAI-derived multifunctional initiation groups can have a much broader impact in the cellulose field as it can be easily adapted for the surface modification of diverse cellulose-based precursors. In the first example, we took advantage of the extremely high reactivity of DMSO-swollen cellulose toward TAI to demonstrate the possibility of spatial control in surface-initiated (SI) grafting from flat cellulose/TAI substrates. To this end, we placed a DMSO-wetted cellulose filter paper (Whatman) into a metallic mask and applied TAI into the mask opening. We then used the purified TAI-modified paper to initiate ATRP of MMA, obtaining within 30 min a thick, macroscopic layer of polymer bound to the regions of the paper surface originally exposed to TAI (Fig. 6). Notably, there was virtually no polymer growth from the rear side of the paper, confirming the instantaneous TAI reaction with the DMSO-wetted paper. We thus envisage that this strategy could be applicable to the fabrication of Janus-type fabrics.^90^

**Fig. 7:**
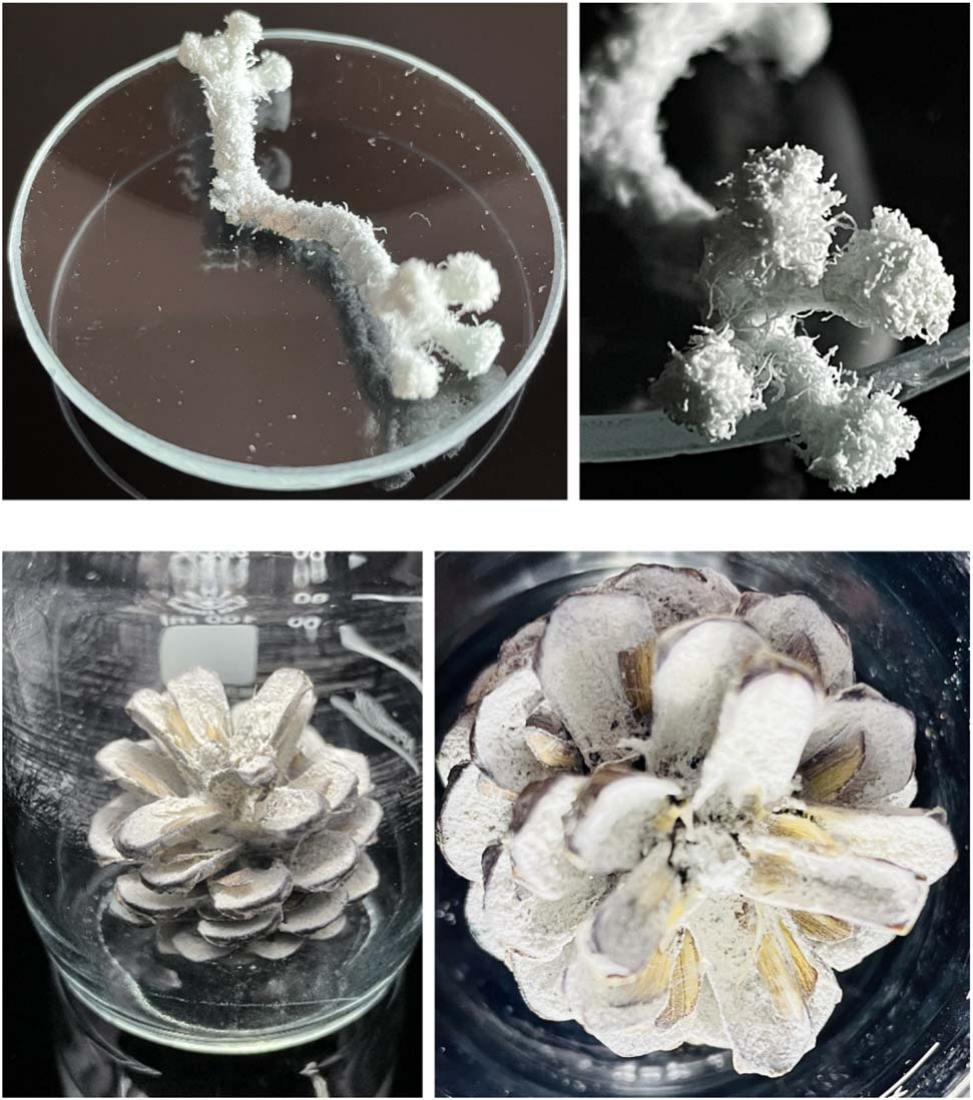
A cotton thread (top) and a pine tree cone (bottom) grafted with poly(MMA) *via* the two step TAI-modification/ATRP-grafting strategy.

In another experiment, 5 cm of a thick cotton thread was surface-modified with TAI in DMSO and subsequently used to trigger MMA polymerization, which led to the complete coverage of the thread with a thick polymer layer (Fig. 7 and S28†). In the close-up picture, the disentanglement of the individual strands at the thread ends and the efficient modification of the smallest thread features is well-visible. Finally, to illustrate the feasibility of this strategy also for more complex (cellulose-based) natural substrates, we successfully grafted a polymer layer from TAI-modified pine tree cone in the same way (Fig. 7 and S29†). The non-modified areas visible on the cone scales correspond to the places where seeds blocked the access of TAI during the modification step (the seeds got released during the polymerization step). This further demonstrates the spatial control in the TAI-based SI grafting strategy. Altogether, these preliminary results show the great potential of the TAI-based strategy in both homogeneous and heterogeneous SI grafting from natural polymeric substrates with efficiency and grafting density unparalleled by the traditional protocols.^30,91^

## Conclusions

In conclusion, we showed in this study that the application of universal multifunctional TAI-based Cu-RDRP initiation sites can significantly extend the “toolset” of synthetic polymer chemists aspiring at constructing CPAs of novel architectures and properties. To assist with this task, we provided here an extensive library of optimized conditions for conducting well-controlled TAG-initiated Cu-RDRP of different monomers. The unique synergistic combination of TAI trifunctionality and extreme reactivity allows for rapid amplification of the functionality of CPA precursor-derived (macro)initiators. As a result, an unprecedently high number of polymeric chains can be easily installed onto CPA precursors, in stark contrast to earlier approaches based on monofunctional initiation sites introduced into precursors *via* inefficient acylations. Resulting opportunities in CPA synthesis were illustrated on multiple relevant scenarios yielding CPAs of novel qualities in uncomplicated protocols.

We envisage that in future the scope of the presented strategy will be significantly extended. For example, the broad reactivity of TAI will extend the range of functional substrates that could serve as CPA precursors; moreover, the different reactivity (stability) of linkers through which precursors are connected to the initiating TAGs could be exploited in programmed CPA decomposition. Furthermore, synthesis of miktoarm star polymers based on telechelic precursors or preparation of ultra-dense polymeric brushes with controlled thickness^92^ represent some of the expected future applications. Last but not least, the study of the physico-chemical properties of the new multi-chain CPAs can be desirable from the viewpoint of future applications of these materials.

## Data availability

Data supporting this article have been included in ESI:† used materials, synthetic protocols, instrumentation and methods, additional experimental and characterization data, additional figures and images.

## Author contributions

SG: investigation, validation, visualization, writing – original draft; MJ: funding acquisition, investigation; EČ: investigation; VR: conceptualization, funding acquisition, investigation, methodology, project administration, supervision, visualization, writing – original draft, writing – review & editing.

## Conflicts of interest

The authors declare no conflict of interest.

## Supplementary Material

SC-015-D4SC01739K-s001

## References

[cit1] Ren J. M., McKenzie T. G., Fu Q., Wong E. H. H., Xu J., An Z., Shanmugam S., Davis T. P., Boyer C., Qiao G. G. (2016). Star Polymers. Chem. Rev..

[cit2] Bosman A. W., Janssen H. M., Meijer E. W. (1999). About Dendrimers: Structure, Physical Properties, and Applications. Chem. Rev..

[cit3] Feng C., Li Y., Yang D., Hu J., Zhang X., Huang X. (2011). Well-defined graft copolymers: from controlled synthesis to multipurpose applications. Chem. Soc. Rev..

[cit4] Sheiko S. S., Sumerlin B. S., Matyjaszewski K. (2008). Cylindrical molecular brushes: Synthesis, characterization, and properties. Prog. Polym. Sci..

[cit5] Kapil K., Szczepaniak G., Martinez M. R., Murata H., Jazani A. M., Jeong J., Das S. R., Matyjaszewski K. (2023). Visible-Light-Mediated Controlled Radical Branching Polymerization in Water. Angew. Chem., Int. Ed..

[cit6] Kakkar A., Traverso G., Farokhzad O. C., Weissleder R., Langer R. (2017). Evolution of macromolecular complexity in drug delivery systems. Nat. Rev. Chem..

[cit7] Detappe A., Nguyen H. V. T., Jiang Y., Agius M. P., Wang W., Mathieu C., Su N. K., Kristufek S. L., Lundberg D. J., Bhagchandani S., Ghobrial I. M., Ghoroghchian P. P., Johnson J. A. (2023). Molecular bottlebrush prodrugs as mono- and triplex combination therapies for multiple myeloma. Nat. Nanotechnol..

[cit8] Johnson J. A., Lu Y. Y., Burts A. O., Lim Y.-H., Finn M. G., Koberstein J. T., Turro N. J., Tirrell D. A., Grubbs R. H. (2011). Core-Clickable PEG-Branch-Azide Bivalent-Bottle-Brush Polymers by ROMP: Grafting-Through and Clicking-To. J. Am. Chem. Soc..

[cit9] Newland B., Zheng Y., Jin Y., Abu-Rub M., Cao H., Wang W., Pandit A. (2012). Single Cyclized Molecule Versus Single Branched Molecule: A Simple and Efficient 3D “Knot” Polymer Structure for Nonviral Gene Delivery. J. Am. Chem. Soc..

[cit10] Sowers M. A., McCombs J. R., Wang Y., Paletta J. T., Morton S. W., Dreaden E. C., Boska M. D., Ottaviani M. F., Hammond P. T., Rajca A., Johnson J. A. (2014). Redox-responsive branched-bottlebrush polymers for in vivo MRI and fluorescence imaging. Nat. Commun..

[cit11] Terashima T., Kamigaito M., Baek K.-Y., Ando T., Sawamoto M. (2003). Polymer Catalysts from Polymerization Catalysts: Direct Encapsulation of Metal Catalyst into Star Polymer Core during Metal-Catalyzed Living Radical Polymerization. J. Am. Chem. Soc..

[cit12] Huang K., Rzayev J. (2009). Well-Defined Organic Nanotubes from Multicomponent Bottlebrush Copolymers. J. Am. Chem. Soc..

[cit13] He Y., Yoon Y. J., Harn Y. W., Biesold-McGee G. V., Liang S., Lin C. H., Tsukruk V. V., Thadhani N., Kang Z., Lin Z. (2019). Unconventional route to dual-shelled organolead halide perovskite nanocrystals with controlled dimensions, surface chemistry, and stabilities. Sci. Adv..

[cit14] Pang X., He Y., Jung J., Lin Z. (2016). 1D nanocrystals with precisely controlled dimensions, compositions, and architectures. Science.

[cit15] Sveinbjörnsson B. R., Weitekamp R. A., Miyake G. M., Xia Y., Atwater H. A., Grubbs R. H. (2012). Rapid self-assembly of brush block copolymers to photonic crystals. Proc. Natl. Acad. Sci. U. S. A..

[cit16] Daniel W. F. M., Burdyńska J., Vatankhah-Varnoosfaderani M., Matyjaszewski K., Paturej J., Rubinstein M., Dobrynin A. V., Sheiko S. S. (2016). Solvent-free, supersoft and superelastic bottlebrush melts and networks. Nat. Mater..

[cit17] Vatankhah-Varnosfaderani M., Daniel W. F. M., Everhart M. H., Pandya A. A., Liang H., Matyjaszewski K., Dobrynin A. V., Sheiko S. S. (2017). Mimicking biological stress–strain behaviour with synthetic elastomers. Nature.

[cit18] Vatankhah-Varnosfaderani M., Keith A. N., Cong Y., Liang H., Rosenthal M., Sztucki M., Clair C., Magonov S., Ivanov D. A., Dobrynin A. V., Sheiko S. S. (2018). Chameleon-like elastomers with molecularly encoded strain-adaptive stiffening and coloration. Science.

[cit19] Braunecker W., Matyjaszewski K. (2007). Controlled/living radical polymerization: Features, developments, and perspectives. Prog. Polym. Sci..

[cit20] Corrigan N., Jung K., Moad G., Hawker C. J., Matyjaszewski K., Boyer C. (2020). Reversible-deactivation radical polymerization (Controlled/living radical polymerization): From discovery to materials design and applications. Prog. Polym. Sci..

[cit21] Sumerlin B. S., Neugebauer D., Matyjaszewski K. (2005). Initiation Efficiency in the Synthesis of Molecular Brushes by Grafting from via Atom Transfer Radical Polymerization. Macromolecules.

[cit22] Zoppe J. O., Ataman N. C., Mocny P., Wang J., Moraes J., Klok H.-A. (2017). Surface-Initiated Controlled Radical Polymerization: State-of-the-Art, Opportunities, and Challenges in Surface and Interface Engineering with Polymer Brushes. Chem. Rev..

[cit23] Destarac M., Bessiere J.-M., Boutevin B. (1998). Atom transfer radical polymerization of styrene initiated by polychloroalkanes and catalyzed by CuCl/2,2′-bipyridine: A kinetic and mechanistic study. J. Polym. Sci., Part A: Polym. Chem..

[cit24] Destarac M., Matyjaszewski K., Boutevin B. (2000). Polychloroalkane initiators in copper-catalyzed atom transfer radical polymerization of (meth)acrylates. Macromol. Chem. Phys..

[cit25] Destarac M., Boutevin B., Matyjaszewski K. (2000). Controlled/Living Radical Polymerization. Am. Chem. Soc..

[cit26] Matyjaszewski K., Tsarevsky N. V. (2009). Nanostructured functional materials prepared by atom transfer radical polymerization. Nat. Chem..

[cit27] Matyjaszewski K. (2018). Advanced Materials by Atom Transfer Radical Polymerization. Adv. Mater..

[cit28] Chen Y., Yang D., Yoon Y. J., Pang X., Wang Z., Jung J., He Y., Harn Y. W., He M., Zhang S., Zhang G., Lin Z. (2017). Hairy Uniform Permanently Ligated Hollow Nanoparticles with Precise Dimension Control and Tunable Optical Properties. J. Am. Chem. Soc..

[cit29] Burdyńska J., Li Y., Aggarwal A. V., Höger S., Sheiko S. S., Matyjaszewski K. (2014). Synthesis and Arm Dissociation in Molecular Stars with a Spoked Wheel Core and Bottlebrush Arms. J. Am. Chem. Soc..

[cit30] Carlmark A., Malmström E. (2002). Atom Transfer Radical Polymerization from Cellulose Fibers at Ambient Temperature. J. Am. Chem. Soc..

[cit31] Vlček P., Janata M., Látalová P., Kríž J., Čadová E., Toman L. (2006). Controlled grafting of cellulose diacetate. Polymer.

[cit32] Vlček P., Raus V., Janata M., Kříž J., Sikora A. (2011). Controlled grafting of cellulose esters using SET-LRP process. J. Polym. Sci., Part A: Polym. Chem..

[cit33] Raus V., Štěpánek M., Uchman M., Šlouf M., Látalová P., Čadová E., Netopilík M., Kříž J., Dybal J., Vlček P. (2011). Cellulose-based graft copolymers with controlled architecture prepared in a homogeneous phase. J. Polym. Sci., Part A: Polym. Chem..

[cit34] Li F., Cao M., Feng Y., Liang R., Fu X., Zhong M. (2019). Site-Specifically Initiated Controlled/Living Branching Radical Polymerization: A Synthetic Route toward Hierarchically Branched Architectures. J. Am. Chem. Soc..

[cit35] Boyer C., Atme A., Waldron C., Anastasaki A., Wilson P., Zetterlund P. B., Haddleton D., Whittaker M. R. (2013). Copper(0)-mediated radical polymerisation in a self-generating biphasic system. Polym. Chem..

[cit36] Aksakal R., Resmini M., Becer C. R. (2016). SET-LRP of acrylates catalyzed by a 1 penny copper coin. Polym. Chem..

[cit37] Pang X., Zhao L., Akinc M., Kim J. K., Lin Z. (2011). Novel Amphiphilic Multi-Arm, Star-Like Block Copolymers as Unimolecular Micelles. Macromolecules.

[cit38] Zaborniak I., Chmielarz P., Wolski K., Grześ G., Wang Z., Górska A., Pielichowska K., Matyjaszewski K. (2022). Maltotriose-based star polymers as self-healing materials. Eur. Polym. J..

[cit39] Samek Z., Buděšínský M. (1979). In situ reactions with trichloroacetyl isocyanate and their application to structural assignment of hydroxy compounds by 1H NMR spectroscopy. A general comment. Collect. Czech. Chem. Commun..

[cit40] Bose A. K., Srinivasan P. R. (1975). NMR spectral studies—XII. Tetrahedron.

[cit41] Goodlett V. W. (1965). Use of In Situ Reactions for Characterization of Alcohols and Glycols by Nuclear Magnetic Resonance. Anal. Chem..

[cit42] Buděšínský M., Samek Z., Tichý M. (1980). In situ reactions of amines and amino alcohols and their application to structural assignment by 1H NMR spectroscopy. Collect. Czech. Chem. Commun..

[cit43] Butler P. E., Mueller W. H. (1966). Simplification of Thiol Nuclear Magnetic Resonance Spectra by in Situ Derivatization. Anal. Chem..

[cit44] Loccufier J., Van Bos M., Schacht E. (1991). Convenient method for the analysis of primary and secondary hydroxyl end groups in polyethers. Polym. Bull..

[cit45] Fallais I., Devaux J., Jérôme R. (2000). End-capping of polystyrene by aliphatic primary amine by derivatization of precursor hydroxyl end group. J. Polym. Sci., Part A: Polym. Chem..

[cit46] Donovan A. R., Moad G. (2005). A novel method for determination of polyester end-groups by NMR spectroscopy. Polymer.

[cit47] Postma A., Davis T. P., Donovan A. R., Li G., Moad G., Mulder R., O'Shea M. S. (2006). A simple method for determining protic end-groups of synthetic polymers by 1H NMR spectroscopy. Polymer.

[cit48] Cao L., Cao B., Lu C., Wang G., Yu L., Ding J. (2015). An injectable hydrogel formed by in situ cross-linking of glycol chitosan and multi-benzaldehyde functionalized PEG analogues for cartilage tissue engineering. J. Mater. Chem. B.

[cit49] Martin J. C., Chitwood J. L., Gott P. G. (1971). Reactions of trichloroacetyl isocyanate with unsaturated ethers. J. Org. Chem..

[cit50] Ando T., Kamigaito M., Sawamoto M. (1997). Design of initiators for living radical polymerization of methyl methacrylate mediated by ruthenium(II) complex. Tetrahedron.

[cit51] Shen Y., Zhu S., Zeng F., Pelton R. (2000). Versatile Initiators for Macromonomer Syntheses of Acrylates, Methacrylates, and Styrene by Atom Transfer Radical Polymerization. Macromolecules.

[cit52] Soriano-Moro G., Percino J., Cerón M., Bañuelos A., Chapela V. M., Castro M. E. (2014). Using of Novel Halides in the ATRP Polymerization. Estimation of Polymer Molecular Mass. Macromol. Symp..

[cit53] Lorandi F., Fantin M., Wang Y., Isse A. A., Gennaro A., Matyjaszewski K. (2020). Atom Transfer Radical Polymerization of Acrylic and Methacrylic Acids: Preparation of Acidic Polymers with Various Architectures. ACS Macro Lett..

[cit54] Alkan S., Toppare L., Hepuzer Y., Yagci Y. (1999). Block copolymers of thiophene-capped poly(methyl methacrylate) with pyrrole. J. Polym. Sci., Part A: Polym. Chem..

[cit55] Bamford C. H., Middleton I. P., Ai-Lamee K. G., Paprotny J. (1987). Halo-isocyanates as ‘transformation’ reagents. Br. Polym. J..

[cit56] Shirai Y., Kawatsura K., Tsubokawa N. (1999). Graft polymerization of vinyl monomers from initiating groups introduced onto polymethylsiloxane-coated titanium dioxide modified with alcoholic hydroxyl groups. Prog. Org. Coat..

[cit57] Shirai Y., Shirai K., Tsubokawa N. (2001). Effective grafting of polymers onto ultrafine silica surface: Photopolymerization of vinyl monomers initiated by the system consisting of trichloroacetyl groups on the surface and Mn2(CO)10. J. Polym. Sci., Part A: Polym. Chem..

[cit58] Shirai Y., Tsubokawa N. (1997). Grafting of polymers onto ultrafine inorganic particle surface: graft polymerization of vinyl monomers initiated by the system consisting of trichloroacetyl groups on the surface and molybdenum hexacarbonyl. React. Funct. Polym..

[cit59] Wei G., Shirai K., Fujiki K., Saitoh H., Yamauchi T., Tsubokawa N. (2004). Grafting of vinyl polymers onto VGCF surface and the electric properties of the polymer-grafted VGCF. Carbon.

[cit60] Lorandi F., Fantin M., Matyjaszewski K. (2022). Atom Transfer Radical Polymerization: A Mechanistic Perspective. J. Am. Chem. Soc..

[cit61] Anastasaki A., Nikolaou V., Nurumbetov G., Wilson P., Kempe K., Quinn J. F., Davis T. P., Whittaker M. R., Haddleton D. M. (2016). Cu(0)-Mediated Living Radical Polymerization: A Versatile Tool for Materials Synthesis. Chem. Rev..

[cit62] Percec V., Guliashvili T., Ladislaw J. S., Wistrand A., Stjerndahl A., Sienkowska M. J., Monteiro M. J., Sahoo S. (2006). Ultrafast Synthesis of Ultrahigh Molar Mass Polymers by Metal-Catalyzed Living Radical Polymerization of Acrylates, Methacrylates, and Vinyl Chloride Mediated by SET at 25 °C. J. Am. Chem. Soc..

[cit63] Rosen B. M., Percec V. (2009). Single-Electron Transfer and Single-Electron Transfer Degenerative Chain Transfer Living Radical Polymerization. Chem. Rev..

[cit64] Zhang Y., Wang Y., Matyjaszewski K. (2011). ATRP of Methyl Acrylate with Metallic Zinc, Magnesium, and Iron as Reducing Agents and Supplemental Activators. Macromolecules.

[cit65] Zhang Y., Wang Y., Peng C.-h., Zhong M., Zhu W., Konkolewicz D., Matyjaszewski K. (2012). Copper-Mediated CRP of Methyl Acrylate in the Presence of Metallic Copper: Effect of Ligand Structure on Reaction Kinetics. Macromolecules.

[cit66] Raus V., Janata M., Čadová E. (2018). Copper Wire–Catalyzed RDRP in Nonpolar Media as a Route to Ultrahigh Molecular Weight Organic–Inorganic Hybrid Polymers. Macromol. Chem. Phys..

[cit67] Leng X., Nguyen N. H., van Beusekom B., Wilson D. A., Percec V. (2013). SET-LRP of 2-hydroxyethyl acrylate in protic and dipolar aprotic solvents. Polym. Chem..

[cit68] Zhang M., Cunningham M. F., Hutchinson R. A. (2015). Aqueous copper(0) mediated reversible deactivation radical polymerization of 2-hydroxyethyl acrylate. Polym. Chem..

[cit69] West A. G., Hornby B., Tom J., Ladmiral V., Harrisson S., Perrier S. (2011). Origin of Initial Uncontrolled Polymerization and Its Suppression in the Copper(0)-Mediated Living Radical Polymerization of Methyl Acrylate in a Nonpolar Solvent. Macromolecules.

[cit70] Hornby B. D., West A. G., Tom J. C., Waterson C., Harrisson S., Perrier S. (2010). Copper(0)-Mediated Living Radical Polymerization of Methyl Methacrylate in a Non-polar Solvent. Macromol. Rapid Commun..

[cit71] Poláková L., Raus V., Kostka L., Braunová A., Pilař J., Lobaz V., Pánek J., Sedláková Z. (2015). Antioxidant Properties of 2-Hydroxyethyl Methacrylate-Based Copolymers with Incorporated Sterically Hindered Amine. Biomacromolecules.

[cit72] Poláková L., Raus V., Cuchalová L., Poręba R., Hrubý M., Kučka J., Větvička D., Trhlíková O., Sedláková Z. (2022). SHARP hydrogel for the treatment of inflammatory bowel disease. Int. J. Pharm..

[cit73] Janata M., Čadová E., Johnson J. W., Raus V. (2023). Diminishing the catalyst concentration in the Cu(0)-RDRP and ATRP synthesis of well-defined low-molecular weight poly(glycidyl methacrylate). J. Polym. Sci..

[cit74] Nguyen N. H., Leng X., Percec V. (2013). Synthesis of ultrahigh molar mass poly(2-hydroxyethyl methacrylate) by single-electron transfer living radical polymerization. Polym. Chem..

[cit75] Gupta S., Raus V. (2023). Cu(0)-RDRP of 2-hydroxyethyl methacrylate in a non-polar solvent enables rapid synthesis of high-molecular weight homopolymers and direct access to amphiphilic copolymers. React. Funct. Polym..

[cit76] Liu T., Li X., Qian Y., Hu X., Liu S. (2012). Multifunctional pH-Disintegrable micellar nanoparticles of asymmetrically functionalized β-cyclodextrin-Based star copolymer covalently conjugated with doxorubicin and DOTA-Gd moieties. Biomaterials.

[cit77] Hu X., Liu S., Huang Y., Chen X., Jing X. (2010). Biodegradable Block Copolymer-Doxorubicin Conjugates via Different Linkages: Preparation, Characterization, and In Vitro Evaluation. Biomacromolecules.

[cit78] Kim B.-S., Lee H.-i., Min Y., Poon Z., Hammond P. T. (2009). Hydrogen-bonded multilayer of pH-responsive polymeric micelles with tannic acid for surface drug delivery. Chem. Commun..

[cit79] Dittert L. W., Higuchi T. (1963). Rates of Hydrolysis of Carbamate and Carbonate Esters in Alkaline Solution. J. Pharm. Sci..

[cit80] Vontor T., Vecera M. (1973). Carbamates 4. Kinetics and Mechanism of Hydrolysis of Substituted Phenyl N-Methylcarbamates in Strongly Alkaline and Acid-Media. Collect. Czech. Chem. Commun..

[cit81] Vandenabeele-Trambouze O., Garrelly L., Mion L., Boiteau L., Commeyras A. (2001). Key parameters for carbamate stability in dilute aqueous–organic solution. Adv. Environ. Res..

[cit82] Ghosh A. K., Brindisi M. (2015). Organic Carbamates in Drug Design and Medicinal Chemistry. J. Med. Chem..

[cit83] Lin C. Y., Coote M. L., Petit A., Richard P., Poli R., Matyjaszewski K. (2007). Ab Initio Study of the Penultimate Effect for the ATRP Activation Step Using Propylene, Methyl Acrylate, and Methyl Methacrylate Monomers. Macromolecules.

[cit84] Rolph M. S., Pitto-Barry A., O'Reilly R. K. (2017). The hydrolytic behavior of N,N′-(dimethylamino)ethyl acrylate-functionalized polymeric stars. Polym. Chem..

[cit85] Zheng Z., Ling J., Müller A. H. E. (2014). Revival of the R-Group Approach: A “CTA-shuttled” Grafting from Approach for Well-Defined Cylindrical Polymer Brushes via RAFT Polymerization. Macromol. Rapid Commun..

[cit86] Raus V., Sturcova A., Dybal J., Slouf M., Vackova T., Salek P., Kobera L., Vlcek P. (2012). Activation of cellulose by 1,4-dioxane for dissolution in N,N-dimethylacetamide/LiCl. Cellulose.

[cit87] Klemm D., Heublein B., Fink H.-P., Bohn A. (2005). Cellulose: Fascinating Biopolymer and Sustainable Raw Material. Angew. Chem., Int. Ed..

[cit88] Potthast A., Radosta S., Saake B., Lebioda S., Heinze T., Henniges U., Isogai A., Koschella A., Kosma P., Rosenau T., Schiehser S., Sixta H., Strlič M., Strobin G., Vorwerg W., Wetzel H. (2015). Comparison testing of methods for gel permeation chromatography of cellulose: coming closer to a standard protocol. Cellulose.

[cit89] Gerle M., Fischer K., Roos S., Müller A. H. E., Schmidt M., Sheiko S. S., Prokhorova S., Möller M. (1999). Main Chain Conformation and Anomalous Elution Behavior of Cylindrical Brushes As Revealed by GPC/MALLS, Light Scattering, and SFM. Macromolecules.

[cit90] Xu Q., Yang J., Zhang X., Wen X., Yamada M., Fu F., Diao H., Liu X. (2020). A “grafting through” strategy for constructing Janus cotton fabric by mist polymerization. J. Mater. Chem. A.

[cit91] Zaborniak I., Chmielarz P. (2023). Polymer-modified regenerated cellulose membranes: following the atom transfer radical polymerization concepts consistent with the principles of green chemistry. Cellulose.

[cit92] Sant S., Klok H.-A. (2024). Linear, Y- and Ψ-shaped poly(2-(dimethylamino)ethyl methacrylate) and poly(methyl methacrylate) brushes prepared by surface-initiated polymerization from a homologous series of ATRP initiators. Eur. Polym. J..

