# Supplementary material for: Straightforward synthesis of complex polymeric architectures with ultra-high chain density
Source: Chem Sci. 2024 Jul 12;15(32):12739–53. doi: 10.1039/d4sc01739k (PMC11323333; doi:10.1039/d4sc01739k)
Supplement: SC-015-D4SC01739K-s001 [file SC-015-D4SC01739K-s001.pdf]

Electronic Supplementary Material (ESI) for Chemical Science.
This journal is © The Royal Society of Chemistry 2024

**Electronic supplementary information for**

# Straightforward synthesis of complex polymeric architectures with ultra-high chain density

*Sachin Gupta, Miroslav Janata, Eva Čadová, and Vladimír Raus**

*Institute of Macromolecular Chemistry, Czech Academy of Sciences, Heyrovského nám. 2, 162 06, Prague 6, Czech Republic*

*E-mail: raus@imc.cas.cz*

## EXPERIMENTAL

### Materials

Trichloroacetyl isocyanate (TAI; Acros Organics, NMR grade, >97%), CuBr (Fluka, >98%), $CuBr_2$ (Sigma Aldrich, 98%) CuCl (Sigma-Aldrich, >99%), $CuCl_2$ (Sigma-Aldrich, 99%), and phenothiazine (Sigma-Aldrich, 98%) were used as received. Cu-wire (Sigma-Aldrich, diameter=0.64 mm) was activated before each polymerization by conc. HCl using the procedure provided below. Lithium chloride (Fluka) was vacuum dried at 190 °C for 8 h prior the use. *N,N,N′,N″,N″*-Pentamethyldiethylenetriamine (PMDETA; Sigma-Aldrich, 99%) was vacuum distilled and stored under argon at 4 °C. Tris[2-(dimethylamino)ethyl]amine ($Me_6$TREN) was synthesized using a literature protocol[1] and stored under argon at 4 °C. Solvents, i.e. dimethyl sulfoxide (DMSO; Acros Organics, 99.7+%), dimethylacetamide (DMAc; Acros Organics, 99.5+% ), 1,4-dioxane (Lach-Ner, p.a.), toluene (Lach-Ner, p.a.), isopropyl alcohol (IPA; Lach-Ner, p.a.), methanol (Lach-Ner, p.a), acetonitrile (Lach-Ner, p.a.), dichloromethane (Lach-Ner, p.a.), tetrahydrofuran (THF; Lach-Ner, p.a.), and acetone (Lach-Ner, p.a.), were either used as received or dried using 3Å molecular sieves and purged with argon for 1 h (when used for polymerization) or dried using 3Å molecular sieves and stored under argon (when used for TAI modifications). Hydrochloric acid (Lach-Ner, 35-38%) was used as received.

*N,N*-Dimethylacetamide (DMAc; VWR; HPLC Grade, 99.5 %) and lithium bromide (Sigma-Aldrich, 99%) were used for the preparation of the mobile phase for SEC with triple detection (TD-SEC); the prepared mobile phase was filtered through a 0.22 µm polyamide filter before use. THF used for SEC with relative calibration (Lach-Ner, p.a.) was distilled before use.

Methyl acrylate (MA; Sigma-Aldrich, 99%), methyl methacrylate (MMA; Acros Organics, 99%), styrene (Fluka, 99.5%), *n*-butyl acrylate (BA; Fluka, 99%), glycidyl methacrylate (GMA; Fluka, 99.5%), 2-hydroxyethyl methacrylate (HEMA; Sigma-Aldrich, 97%), and *n*-butyl methacrylate (BMA; Fluka, 99%) were distilled under high vacuum to remove the inhibitor and stored under argon atmosphere at -20°C. 2-Hydroxyethyl acrylate (HEA; Sigma-Aldrich, 96%) was used as received.

Pentaerythritol (Sigma-Aldrich, 98%), ethylene glycol (Fluka, >99.5%), *N,N*-diisopropylamine (DIPA; Sigma-Aldrich, >99.5% ), cellulose Avicel PH-101 (Fluka) and Sigmacell type 101 (Sigma), filter paper Whatman 1450-917, cotton thread Catania (Schachenmayr smc, 100% cotton), and ß-cyclodextrin (Sigma-Aldrich, ≥97%) were treated before the reaction with TAI as detailed in the experimental protocols.

### Characterization

The number-average molecular weights ($M_n$), weight-average molecular weights ($M_w$), and dispersity ($Đ$) of the (co)polymers were determined by SEC.

Most of the analyses during the optimization of polymerization conditions were performed using an SEC system consisting of the SDS 150 pump (Watrex, Czech Republic), an RI detector (RI-101; Shodex, Japan), and two PLgel MIXED-C columns (300 × 7.5 mm, SDV gel with particle size 5µm; Agilent, USA). Tetrahydrofuran was used as the mobile phase at 25 °C with a flow rate of 1 mL/min. The molecular weight (MW) values were calculated using the Clarity software (Dataapex, Czech Republic). Calibrations with polystyrene standards (PSS, Germany) in the molecular weight range of 580 and

1 820 000 and with poly(MMA) standards (PSS, Germany) in the MW range of 2 200 to 1 220 000 were used.

Advanced polymer characterization was done using the Malvern Panalytical OMNISEC triple detection SEC (TD-SEC) system consisting of OMNISEC Resolve and OMNISEC Reveal units. Two PSS GRAM analytical linear columns with the dimensions of 8 x 300 mm and the particle size of 10 μm were used. Triple detection with the following detectors was performed: differential refractive index (RI) detector, right-angle light scattering (RALS) + low-angle light scattering (LALS) measuring at an angle of 7° to the incident beam (laser wavelength of 640 nm), and a 4-capillary Wheatstone bridge viscometer. The columns and detectors were held at 55 °C. Dimethylacetamide with 5g/L LiBr was used as an eluent at a flow rate of 1 mL/min. OMNISEC software from Malvern Panalytical was used for online monitoring and processing of the data. In some cases, universal calibration was used for the MW determination; in this case, the calibration was performed using the polystyrene standards (PSS, Germany) in the MW range of 1,930 to 990,500. All sample solutions were filtered through 0.2 μm PTFE filters prior to injection.

$^{1}$H NMR and $^{13}$C NMR spectra were recorded on a Bruker Avance NEO 400 spectrometer operating at 400.13 MHz at 300 K or Bruker AVANCE-III operating at 600 MHz at 298 K.

**Procedures**

Note: All reactions with TAI as well as polymerizations were conducted using a Schlenk-type technique in inert atmosphere (under argon) unless stated otherwise.

***Activation of Cu wire***

The wire was placed into ca. 5 mL of conc. HCl for 5 min, removed and washed with water, and returned to conc. HCl for another 10 min. Afterwards, the activated wire was successively washed with water and acetone, dried in an argon stream, and kept under argon until use.

***Methyl(trichloroacetyl)carbamate (MTAC)***

Into a 50 mL reaction flask equipped with a magnetic stirring bar and a three-way stopcock connected to an argon/vacuum inlet, dry methanol (20 mL) was added, and the flask was cooled in an ice bath. Upon dropwise addition of TAI (6.325 mL, 53.08 mmol), the flask was removed from the ice bath, and the mixture was left to stir at r.t. for 1 h. Thereafter, unreacted methanol was evaporated to afford a white solid (11.3 g, 97% yield) that was stored at 4 °C and used as a Cu-RDRP initiator without any further purification.

***1,1-Diisopropyl-3-(2,2,2-trichloroacetyl)-urea (DTAU)***

Into a 25 mL two-necked round-bottomed flask equipped with a magnetic stirring bar and connected via a distillation bridge to another flask having an argon/vacuum inlet, dried $CH_2Cl_2$ (5 mL) and *N,N*-diisopropylamine (0.420 mL, 0.2965 mmol, pre-dried using 3Å molecular sieves) were added. The flask

was placed in an ice bath, and TAI (0.442 mL, 0.3706 mmol) was added dropwise. Once the addition of TAI was complete, the flask was removed from the ice bath, and the mixture was left to stir at r. t. for 20 minutes during which the solution turned slight yellow. Thereafter, $CH_2Cl_2$ and the excess of TAI were distilled off under high vacuum, affording a slightly yellow solid (0.834 g, 97% yield) that was further dried under vacuum and stored at 4 °C. The product was used as a Cu-RDRP initiator without any further purification.

***Pentaerythritol tetrakis((2,2,2-trichloroacetyl)carbamate) (PTAC)***

In a 50 mL two-necked round-bottomed flask equipped with a magnetic stirring bar and connected via a distillation bridge to another flask having an argon/vacuum inlet, pentaerythritol (0.25 g, 1.836 mmol, pre-dried under vacuum at 80 °C overnight) was mixed with dried dioxane (10 mL). Then, TAI (1.1 mL, 9.231 mmol) was added dropwise, which led to the dissolution of the solids. After 20 min, dioxane and the excess of TAI were distilled off under high vacuum, affording a white solid (1.513 g, 93% yield) that was further dried under vacuum and stored at 4 °C. The product was used as a Cu-RDRP initiator without any further purification.

***Ethane-1,2-diyl bis((2,2,2-trichloroacetyl)carbamate) (ETAC)***

Into a 25 mL two-necked round-bottomed flask equipped with a magnetic stirring bar and connected via a distillation bridge to another flask having an argon/vacuum inlet, dried $CH_2Cl_2$ (5 mL) and ethylene glycol (0.112 mL, 0.2014 mmol, pre-dried using 3Å molecular sieves) were added. The flask was placed in an ice bath, and TAI (0.600 mL, 0.5035 mmol) was added dropwise. Once the addition of TAI was complete, the flask was removed from the ice bath, and the mixture was left to stir at r. t. for 20 min. Thereafter, $CH_2Cl_2$ and the excess of TAI were distilled off under the high vacuum, affording a white solid (0.865 g, 98% yield) that was further dried under vacuum and stored at 4 °C. The product was used as a Cu-RDRP initiator without any further purification.

***Polymerization experiments***

During the optimization of polymerization conditions, Cu-RDRP experiments were conducted in a similar way regardless of the monomer used. Generally, the experimental scale was kept the same, i.e., 5 mL of a monomer and 5 mL of a solvent were used, with the amount of initiator adjusted based on the targeted M/I ratio. In the bulk polymerization of styrene, 10 mL of the monomer was used to keep a similar polymerization mixture volume. In Cu(0)-RDRP experiments, a fixed length of Cu wire (10 cm) was employed unless stated otherwise. Since the initiators used here are solids, in the polymerization protocols, we generally first deoxygenated the solid compounds (an initiator, Cu-salt(s), or Cu wire), added a solvent and a monomer, and finally started the polymerization by adding a ligand (and placing the flask into a heating bath if needed). Typically, the polymerization was stopped either when the viscosity of the polymerization mixture prevented efficient stirring or when 24 h elapsed. Monomer conversions were typically determined by gravimetry (for styrene and MMA) or using $^1$H NMR (for other monomers). Below, we provide sample procedures for Cu(0)-RDRP and ATRP experiments.

*Cu(0)-RDRP of MMA*

MTAC (51.52 mg, 0.2337 mmol) and activated Cu wire (10 cm) were placed into a reaction flask equipped with a magnetic stirring bar and a three-way stopcock connected to an argon/vacuum inlet. After thorough deoxygenation by several vacuum-argon cycles, toluene (5 mL) was added, followed by the addition of MMA (5 mL, 46.74 mmol). Subsequently, the polymerization was started by the addition of PMDETA (49 µL, 0.2337 mmol), and the flask was placed into a stirred oil bath pre-heated to 85 °C. After 24 h, the experiment was ended, the flask was cooled down, the Cu-wire was removed, and the polymerization was quenched by adding a small amount of phenothiazine. Then, the mixture was diluted with THF and the product was precipitated in MeOH/water (4:1 v/v). The precipitate was collected on a glass frit, washed, and dried overnight under vacuum at 40 °C.

*ATRP of styrene*

CuBr (62.4 mg, 0.4349 mmol) and MTAC (96 mg, 0.4349 mmol) were placed into a reaction flask equipped with a magnetic stirring bar and a three-way stopcock connected to an argon/vacuum inlet. After thorough deoxygenation by several vacuum-argon cycles, styrene (10 mL, 86.98 mmol) was added. Subsequently, the polymerization was started by the addition of $Me_6$TREN (116 µL, 0.4349 mmol), and the flask was placed into a stirred oil bath pre-heated to 110 °C. After 6 h, the mixture was highly viscous, and so the experiment was ended, the flask was cooled down, and the polymerization was quenched by adding a small amount of phenothiazine. Then, the mixture was diluted with THF and the product was precipitated in MeOH. The precipitate was collected on a glass frit, washed, and dried overnight under vacuum at 40 °C.

***Alkaline hydrolysis of star polymers and graft copolymers***

Reactions were carried out according to the literature.[2] In a 25 mL flask equipped with a magnetic stirring bar, a (co)polymer (100 mg), THF (8 mL), and 1 M solution of KOH in methanol (4 mL) were mixed, and the reaction mixture was stirred for 3 days at r. t. Then, the mixture was neutralized with 1 M HCl, solvents were evaporated, and the residuum was extracted with THF (3 mL). The product was then precipitated by the addition of the extract into MeOH/water (4:1 v/v) in case of poly(MMA) or neat MeOH in case of polystyrene. The precipitates were filtered, washed, and dried in vacuum at 40 °C.

***Hydrolytic stability of the TAI-based carbamate linker***

*Poly(2-hydroxyethyl) acrylate (poly(HEA)):* The preparation of the poly(HEA) starting polymer for the stability study was performed via Cu(0)-RDRP in DMSO at 60 °C using the standard procedure detailed above. Upon termination of the polymerization, the reaction mixture was dialyzed against deionized water, and the product was obtained by freeze-drying.

*Attempted hydrolysis of the carbamate linker in poly(HEA) at different pH:* In a 20 mL vial equipped with a magnetic stirring bar, 20 mg of the synthesized poly(HEA) was dissolved in the selected buffer (4 mL; pH = 1, 3, 5, 7, 9, or 11), and the solution was stirred at 37 °C for 24 h. Afterward, the solution was dialyzed against deionized water (MWCO = 1 000) and freeze-dried. The obtained polymer was

analyzed by TD-SEC in DMAc/LiBr (dn/dc was calculated by the OMNISEC software considering 100% sample recovery).

***De novo one-pot synthesis of poly(HEMA-co-MMA)-graft-poly(MMA) graft copolymer***

*Step 1:* Activated Cu wire (4 cm) and MTAC (55.11 mg, 0.25 mmol) were placed into a reaction flask equipped with a magnetic stirring bar and a three-way stopcock connected to an argon/vacuum inlet. After thorough deoxygenation by several vacuum-argon cycles, dioxane (2.75 mL), HEMA (0.606 mL, 5.0 mmol), and MMA (2.14 mL, 20.0 mmol) were added. Subsequently, the polymerization was started by the addition of PMDETA (52.2 µL, 0.25 mmol), and the flask was placed into a stirred oil bath pre-heated to 85 °C. After 3 h, the flask was removed from the oil bath and cooled to r. t. A sample of the reaction mixture was withdrawn for SEC and NMR analysis.

*Step 2*: The polymerization mixture was diluted with dioxane (5 mL), which was followed by a dropwise addition of TAI (298 µL, 2.5 mmol, 0.5 eq. toward HEMA) under intensive stirring. The resulting mixture was further stirred at r. t. for 15 min. Then, 9 mL of the reaction mixture was removed from the flask: a sample was used for $^{1}$H NMR analysis, and the rest was isolated by precipitation in MeOH/water (4:1 v/v). The 1.5 mL of reaction mixture that remained in the reaction flask was used as a macroinitiator in the following step.

*Step 3:* The macroinitiator solution was diluted with dioxane (5 mL), and MMA (15 mL, 140 mmol, 400 eq. toward present TAGs) and PMDETA (75 µL, 0.357 mmol, 1.0 eq. toward the present TAGs) were added, and the flask was placed into a stirred oil bath pre-heated to 85 °C. After 2 h, the experiment was ended, the flask was cooled down, the Cu-wire was removed, and the polymerization was quenched by adding a small amount of phenothiazine. Then, the mixture was diluted with THF and the product was precipitated in MeOH. The precipitate was collected on a glass frit, washed, and dried overnight under vacuum at 40 °C. A sample of the obtained product was further subjected to alkaline hydrolysis.

***Synthesis of multi-arm poly(MMA) stars based on a ß-cyclodextrin core***

*Modification of ß-cyclodextrin with TAI:* In a 5 mL round-bottomed flask equipped with a magnetic stirring bar and an argon/vacuum inlet, ß-CD (0.030 g, 0.0264 mmol, pre-dried under vacuum at 80 °C) was dispersed in dried acetonitrile (800 µL). Then, TAI (95 µL, 0.793 mmol) was added, and the mixture was stirred at r.t. for 16 h. Into the obtained clear solution of the ß-CD/TAI adduct, DMSO (~17 µL) was added to quench the excess of TAI. Samples were withdrawn for $^{1}$H NMR and SEC analyses, and the remaining mixture was used as an initiator in the subsequent step.

*ATRP of MMA initiated by the ß-CD/TAI adduct:* CuBr (57 mg, 0.397 mmol) was placed into a reaction flask equipped with a magnetic stirring bar and a three-way stopcock connected to an argon/vacuum inlet. After thorough deoxygenation by several vacuum-argon cycles, dioxane (5.9 mL), MMA (5.9 mL, 55.15 mmol), and 400 µL of the solution of ß-CD/TAI adduct prepared in the previous step (0.397 mmol of TAGs) were added. Afterward, the polymerization was started by the addition of PMDETA (83 µL, 0.397 mmol), and the flask was placed into a stirred oil bath pre-heated to 85°C. Samples of the polymerization mixture, withdrawn at 4 h and 7 h timepoints, were quenched with phenothiazine.

diluted with THF, precipitated in MeOH, and used for SEC and NMR analyses. After 23 h, the experiment was ended, the flask was cooled down and opened to air, and the polymerization was quenched by adding a small amount of phenothiazine. The stabilized removed samples as well as the final mixture were analyzed by $^{1}$H HMR (conversion determination). Products were isolated as follows: the mixture was diluted with THF, the polymer was precipitated in MeOH, the precipitate was collected on a glass frit, washed, and dried overnight under vacuum at 40 °C. Isolated samples were analyzed by TD-SEC.

***Modification of powder cellulose with TAI***

*Modification in DMAc/LiCl (homogeneous modification):* Cellulose AVICEL PH-101 was activated by dioxane according to the literature protocol (the full activation protocol finished with freeze drying).[3] In a 25 mL reaction flask, activated cellulose (0.1 g, 0.617 mmol of monomeric units) was dissolved in 7.7% DMAc/LiCl (10 mL; prepared under anhydrous conditions). To the stirred solution, TAI (294 µL, 2.467 mmol) was added dropwise. After 20 min, the excess of TAI was quenched with several drops of water, and the product was precipitated in IPA/water (1:1, v/v), collected on a glass frit, washed thoroughly with IPA, and dried in vacuum at 40 °C overnight. Product weight = 0.416 g (93% yield).

*Modification in other solvents (heterogeneous modification):* Modification of powder cellulose in other solvents was done in a similar way as described above, starting with the cellulose suspension in the respective solvent.

***Synthesis of an ultra-dense bottle-brush graft copolymer by ATRP grafting of MMA from cellulose/TAI adduct***

*Preparation of the cellulose/TAI macroinitiator:* Into a 10 mL round-bottomed flask, equipped with a magnetic stirring bar and an argon/vacuum inlet, containing the Avicel PH-101 (0.050 g, 0.3084 mmol, pre-dried in vacuum at 80 °C) suspension in dried acetonitrile (5 mL), TAI (0.221 mL, 1.8546 mmol) was added, and the mixture was left to stir at r. t. for 4 days. Then, MeOH (0.040 mL) was added into the homogeneous solution to quench any unreacted TAI. The prepared mixture was analyzed by SEC and used as a stock solution of the (macro)initiator in the subsequent polymerization step.

*ATRP grafting of MMA from the* cellulose/TAI adduct: CuBr (13.3 mg, 0.0927 mmol) was placed into a 50 mL reaction flask equipped with a magnetic stirring bar and a three-way stopcock connected to an argon/vacuum inlet. After thorough deoxygenation by several vacuum-argon cycles, dioxane (7.9 mL), MMA (7.9 mL, 74.2 mmol), and the (macro)initiator solution from the previous step (250 µL, 0.0927 mmol of TAGs) were added. Afterward, the polymerization was started by the addition of PMDETA (19.4 µL, 0.0927 mmol), and the flask was placed into a stirred oil bath pre-heated to 85°C. At 5 h, a sample of the polymerization mixture was withdrawn for conversion determination by $^{1}$H NMR and for TD-SEC analysis (performed using the polymer isolated by precipitation into MeOH). At 24 h, the experiment was ended, the flask was cooled down and opened to air, and the polymerization was quenched by adding a small amount of phenothiazine. A sample was withdrawn for conversion determination via $^{1}$H NMR. Then, the mixture was diluted with THF and the product was precipitated

in MeOH. The precipitate was collected on a glass frit, washed, and dried overnight in vacuum at 40 °C.

**Surface-initiated grafting from TAI-modified filter paper**

*Modification of Whatman paper with TAI:* Whatman paper (7 x 5.5 cm) was cut and soaked in dry DMSO for 3 days. Then, the paper was removed from DMSO, briefly dried with a paper towel, and placed on a customized mask (see picture below) with an "IMC" inscription. After closely tightening the paper inside the mask, TAI (100 µL, 0.8392 mmol) was dripped evenly onto the exposed paper surface in argon flow. Subsequently, the mask was immersed into a beaker containing IPA in order to quench the excess of TAI. The paper was then removed from the mask and washed excessively with methanol in order to remove any unbound TAI adducts and dried in vacuum at r.t.

*ATRP grafting of MMA from the TAI-modified paper:* CuBr (120.4 mg, 0.8392 mmol) and the TAI-modified Whatman paper were placed into a reaction flask equipped with a magnetic stirring bar and a three-way stopcock connected to an argon/vacuum inlet. After thorough deoxygenation by several vacuum-argon cycles, dioxane (17.5 mL) and MMA (17.5 mL, 163.6 mmol) were added. Afterward, the polymerization was started by the addition of PMDETA (175 µL, 0.8392 mmol), and the flask was placed into a stirred oil bath pre-heated to 85°C. After 30 min, the experiment was ended, the flask was cooled down, and the polymerization was quenched by adding a small amount of phenothiazine. The paper was removed, washed carefully first with THF to remove any free polymer and then with methanol to remove the catalytic complex residua, and finally dried in vacuum at r.t.

**Surface-initiated grafting from TAI-modified cotton thread**

*Modification of cotton thread with TAI:* 5 cm long cotton thread was placed into a reaction flask equipped with a magnetic stirring bar and a three-way stopcock connected to an argon/vacuum inlet. After inertization, dry DMSO (10 mL) was added, and the thread was left to soak for 5 minutes after which TAI (400 µL, 3.356 mmol) was added. After 15 minutes, the thread was removed from the flask, washed with an excess of methanol, and dried in vacuum at r.t.

*ATRP grafting of MMA from the TAI-modified cotton thread:* CuBr (34.4 mg, 0.2398 mmol) and TAI-modified cotton thread were placed into a reaction flask equipped with a magnetic stirring bar and a three-way stopcock connected to an argon/vacuum inlet. After thorough deoxygenation by several vacuum-argon cycles, dioxane (5 mL) and MMA (5 mL, 46.74 mmol) were added. Afterward, the polymerization was started by the addition of PMDETA (50 µL, 0.2398 mmol), and the flask was placed into a stirred oil bath pre-heated to 85°C. After 1 h, the experiment was ended, the flask was cooled down, and the polymerization was quenched by adding a small amount of phenothiazine. The thread was removed, washed thoroughly with THF and methanol, and dried in vacuum at 40 °C.

**Surface-initiated grafting from TAI-modified pine cone**

*Modification of a pine cone with TAI:* A pine cone was left to soak in dry DMSO (80 mL) overnight. The original (discolored) DMSO was then replaced with a fresh one, and 3Å molecular sieves were added. After 7 days, the cone was quickly transferred into a 100 mL wide-neck reagent bottle equipped with a magnetic stirring bar and fitted with a rubber septum pierced with a needle connected to an

argon/vacuum inlet. After inertization, dry DMSO (70 mL) was added, and the bottle was placed in an ice bath. TAI (2 mL, 16.78 mmol) was then added dropwise, and the mixture was then stirred for 15 minutes. Thereafter, the cone was removed from the flask, washed thoroughly with methanol, and dried in vacuum at r.t.

*ATRP grafting of MMA from the TAI-modified pine cone:* CuBr (137.4 mg, 0.958 mmol) and the TAI-modified cone were placed into a into a 100 mL wide-neck reagent bottle equipped with a magnetic stirring bar and fitted with a rubber septum pierced with a needle connected to an argon/vacuum inlet. After thorough deoxygenation, dioxane (40 mL) and MMA (40 mL, 374 mmol) were added. Afterward, the polymerization was started by the addition of PMDETA (200 µL, 0.958 mmol), and the flask was placed into a stirred oil bath pre-heated to 85°C. After 4 h, the experiment was ended, the flask was cooled down, and the polymerization was quenched by adding a small amount of phenothiazine. The cone was removed, washed thoroughly with THF and methanol, and dried in vacuum at 40 °C.

## ADDITIONAL RESULTS

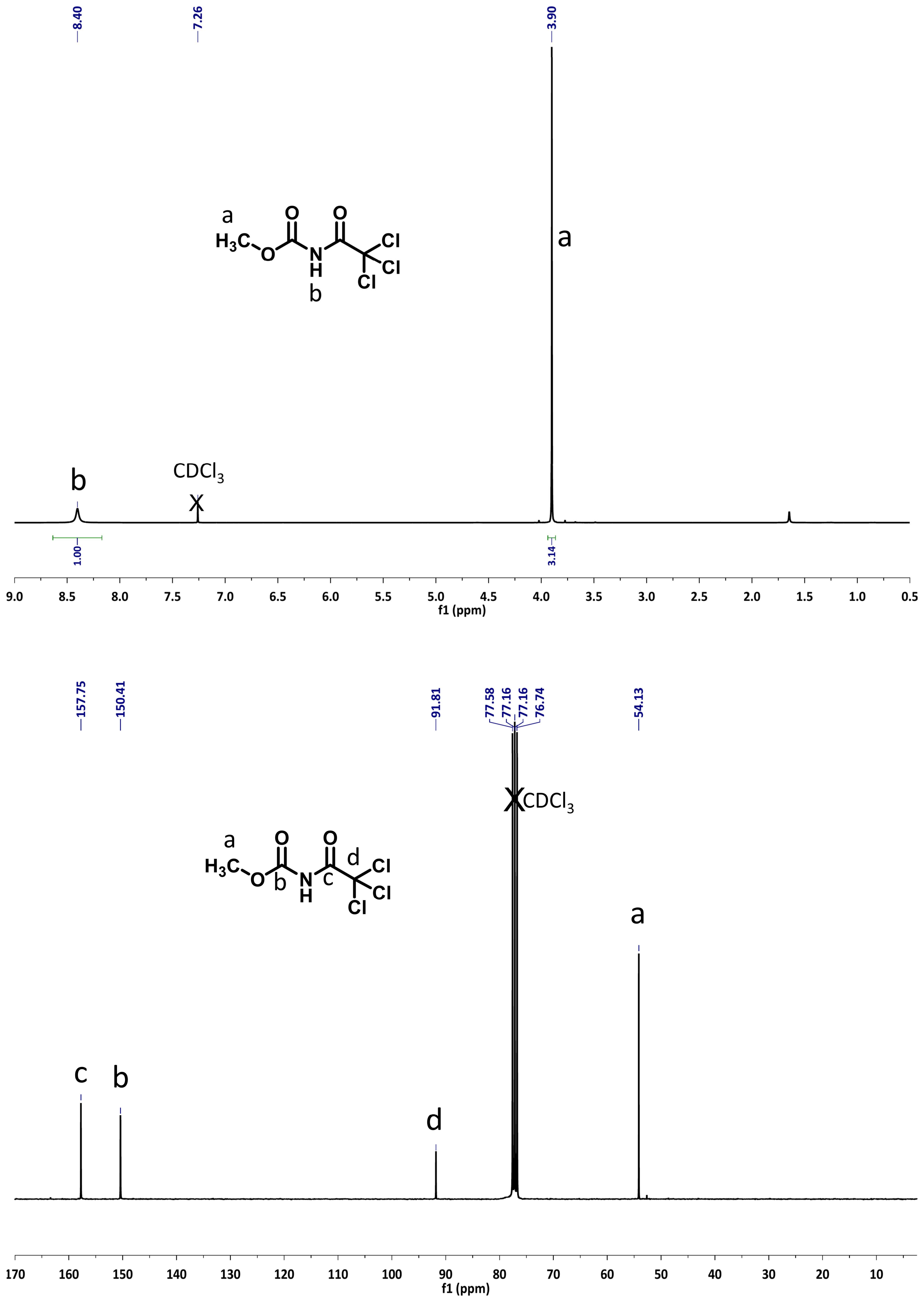


**Figure S1.** $^{1}H$ NMR (top) and $^{13}C$ NMR (bottom) spectra of methyl(trichloroacetyl)carbamate (MTAC) measured in $CDCl_3$.

**Table S1**. Additional results obtained during the optimization of polymerization conditions for MTAC-initiated Cu-RDRP of MA[a]

| Entry | Cat. | Solvent | Ligand (eq.) | T (°C) | Time (h) | Conv. (%)[b] | $M_n$ (theor.)[c] | $M_n$ (SEC)[d] | $Đ$[d] |
|---|---|---|---|---|---|---|---|---|---|
| 1 | CuBr | DMSO | PMDETA (1.0) | r.t. | 24 | | *no polymerization* | | |
| 2 | CuBr | DMSO | $Me_6$TREN (1.0) | r.t. | 24 | | *no polymerization* | | |
| 3 | CuBr | DMSO | PMDETA (1.0) | 60 | 24 | | *no polymerization* | | |
| 4 | CuBr | DMSO | $Me_6$TREN (1.0) | 60 | 24 | | *no polymerization* | | |
| 5 | CuBr | DMAC | PMDETA (1.0) | r.t. | 24 | | *no polymerization* | | |
| 6 | CuBr | DMAC | $Me_6$TREN (1.0) | r.t. | 24 | | *no polymerization* | | |
| 7 | CuBr | DMAC | PMDETA (1.0) | 60 | 24 | | *no polymerization* | | |
| 8 | CuBr | DMAC | $Me_6$TREN (1.0) | 60 | 24 | | *no polymerization* | | |
| 9 | CuBr | toluene | PMDETA (1.0) | r.t. | 24 | | *no polymerization* | | |
| 10 | CuBr | toluene | $Me_6$TREN (1.0) | r.t. | 24 | | *no polymerization* | | |
| 11 | CuBr | toluene | PMDETA (1.0) | 60 | 24 | | *no polymerization* | | |
| 12 | CuBr | toluene | $Me_6$TREN (1.0) | 60 | 24 | 36 | 6 300 | 6 100 | 2.51 |
| 13 | CuBr | dioxane | PMDETA (1.0) | 60 | 24 | | *no polymerization* | | |
| 14 | CuBr | dioxane | $Me_6$TREN (1.0) | 60 | 24 | | *no polymerization* | | |
| 15 | CuCl[e] | dioxane | $Me_6$TREN (1.5) | 60 | 24 | | *no polymerization* | | |
| 16 | Cu(0) | DMAc | PMDETA (0.2) | r.t. | 24 | 32 | 5 700 | 8 200 | 1.74 |
| 17 | Cu(0) | DMAc | PMDETA (0.5) | r.t. | 24 | 18 | 3 300 | 6 000 | 1.55 |
| 18 | Cu(0) | DMAc | PMDETA (1.0) | r.t. | 24 | 22 | 4 000 | 12 400 | 1.34 |
| 19 | Cu(0) | DMAc | $Me_6$TREN (0.2) | r.t. | 24 | | *no polymerization* | | |
| 20 | Cu(0) | DMAc | $Me_6$TREN (0.5) | r.t. | 24 | 29 | 5 100 | 5 300 | 1.23 |
| 21 | Cu(0) | DMAc | $Me_6$TREN (1.0) | r.t. | 24 | 71 | 12 300 | 18 700 | 1.24 |
| 22 | Cu(0) | DMAc | $Me_6$TREN (0.2) | 60 | 24 | 77 | 13 300 | 15 700 | 1.19 |
| 23 | Cu(0) | DMSO | PMDETA (0.2) | r.t. | 24 | 82 | 14 100 | 18 700 | 1.26 |
| 24 | Cu(0) | DMSO | PMDETA (0.5) | r.t. | 24 | 97 | 16 700 | 26 100 | 1.22 |
| 25 | Cu(0) | DMSO | PMDETA (0.5) | 60 | 4 | 99 | 17 100 | 24 800 | 1.25 |
| 26 | Cu(0) | DMSO | PMDETA (1.0) | r.t. | 1 | 36 | 6 300 | 6 900 | 6.49 |
| 27 | Cu(0) | DMSO | $Me_6$TREN (0.2) | r.t. | 24 | | *no polymerization* | | |
| 28 | Cu(0)[f] | DMSO | $Me_6$TREN (0.2) | r.t. | 24 | 22 | 4 000 | 4 600 | 1.29 |
| 29 | Cu(0) | DMSO | $Me_6$TREN (0.2) | 60 | 5 | 88 | 15 200 | 18 400 | 1.21 |
| 30 | Cu(0) | DMSO | $Me_6$TREN (1.0) | r.t. | 24 | 94 | 16 200 | 24 300 | 1.35 |
| 31 | Cu(0)[f] | DMSO | $Me_6$TREN (1.0) | r.t. | 24 | 94 | 16 200 | 25 500 | 1.25 |
| 32 | Cu(0) | toluene | $Me_6$TREN (0.5) | r.t. | 24 | 3 | 730 | 3 000 | 1.44 |
| 33 | Cu(0) | toluene | PMDETA (0.5) | 60 | 24 | 28 | 5 000 | 12 500 | 1.81 |
| 34 | Cu(0) | dioxane | PMDETA (0.5) | 60 | 24 | 18 | 3 300 | 10 000 | 1.82 |

[a] Standard polymerization conditions: M/I = 200:1; monomer/solvent = 1:1 (v/v), catalyst (Cat.): 10 cm of activated copper wire in Cu(0)-RDRP, 1 eq. of Cu(I) salt in ATRP.

[b] Monomer conversion determined by $^1$H NMR.

[c] Theoretical $M_n$ calculated from the M/I ratio and conversion, assuming 100% initiation efficiency.

[d] Determined by SEC with poly(MMA) calibration.

[e] $CuCl_2$ (0.5 eq.) was added as a deactivator.

[f] $CuCl_2$ (0.05 eq.) was added as a deactivator.

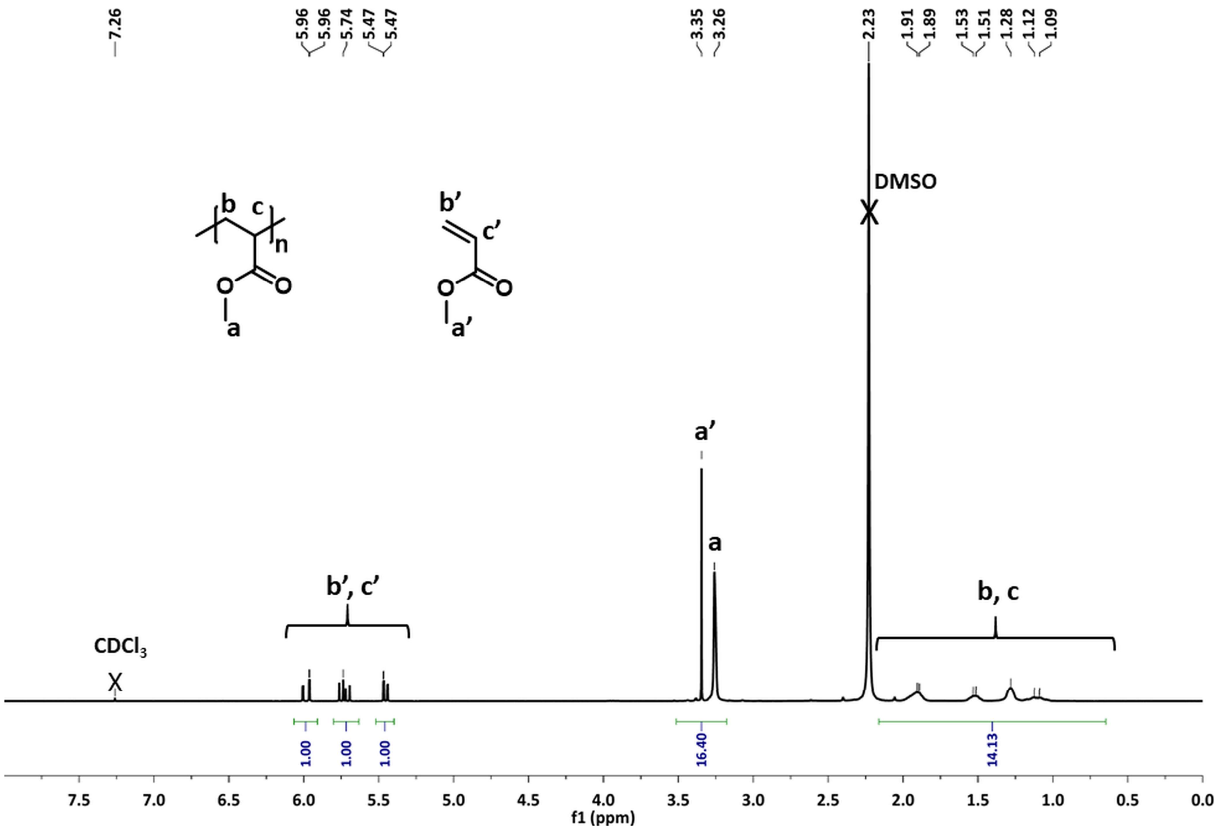


**Figure S2.** A representative $^1$H NMR spectrum (measured in $CDCl_3$) of a reaction mixture from Cu(0)-RDRP of MA (entry 23, Table S1). Monomer conversion was calculated by comparing the intensity of the signals of unreacted monomer (b’,c’) to that of the combined signals of the monomer and polymer (a,a’): conversion (%) = $[1 - (I_{b',c'}/I_{a',a})] \times 100$

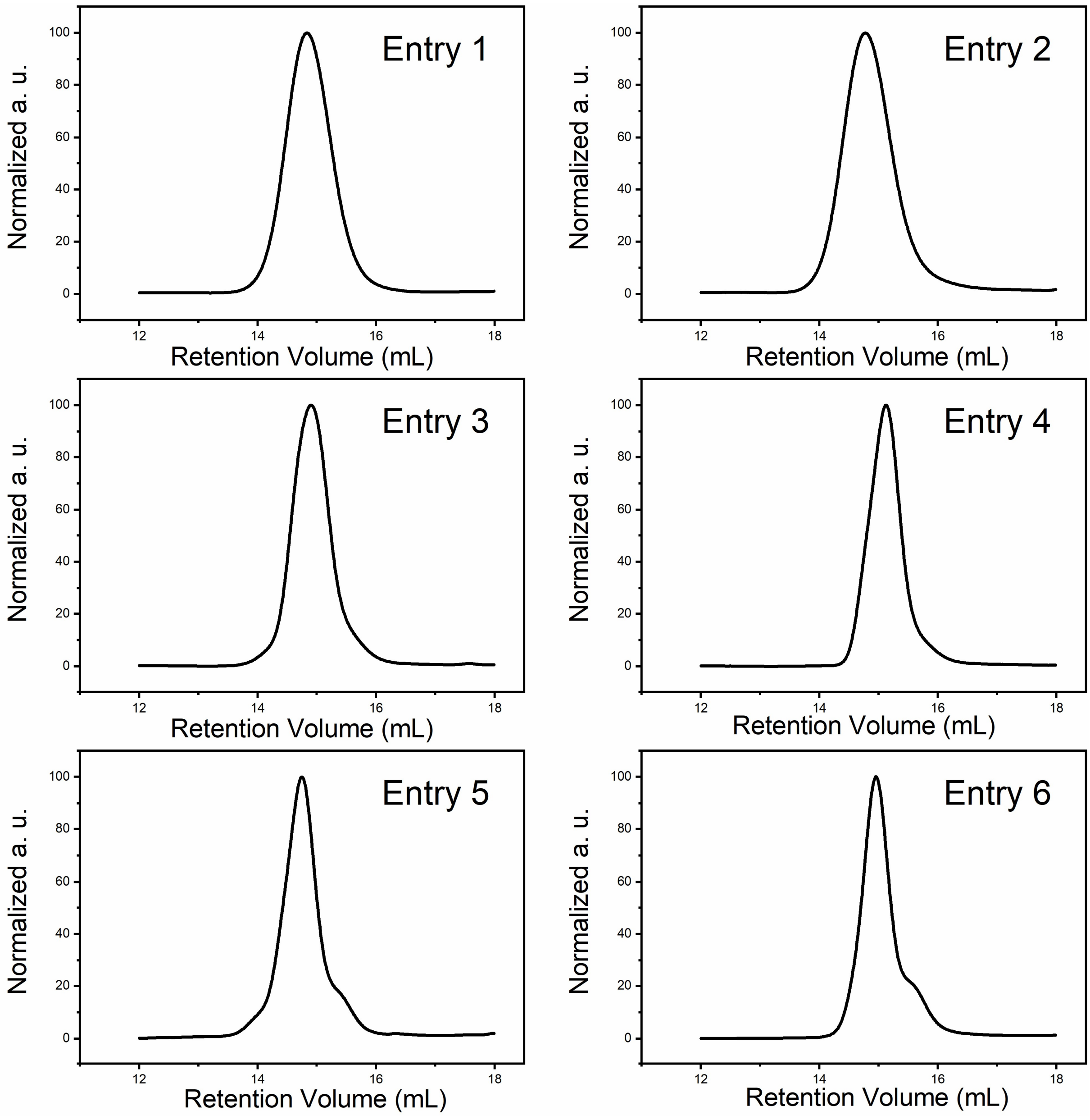


**Figure S3.** SEC elugrams (RI traces) for poly(MA) prepared by MTAC-initiated Cu(0)-RDRP at M/I = 200:1 under optimized conditions (description corresponds to Table 1).

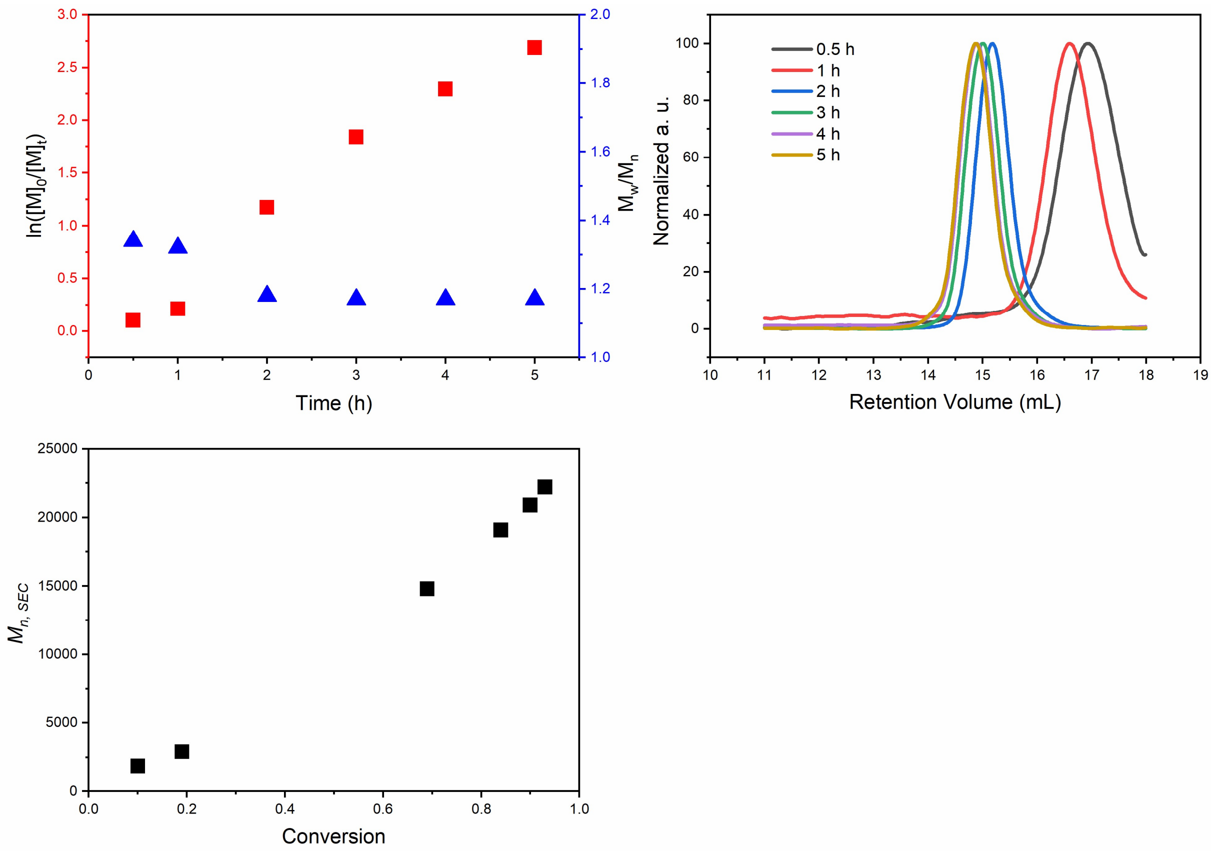


| Time (h) | Conv. (%)[a] | $M_n$ (theor.)[b] | $M_n$ (SEC)[c] | $Đ$[c] |
|---|---|---|---|---|
| 0.5 | 10 | 1 850 | 1 850 | 1.34 |
| 1 | 19 | 3 400 | 2 900 | 1.32 |
| 2 | 69 | 12 000 | 14 800 | 1.18 |
| 3 | 84 | 14 500 | 19 100 | 1.17 |
| 4 | 90 | 15 500 | 20 900 | 1.17 |
| 5 | 93 | 16 000 | 22 200 | 1.17 |

[a] Monomer conversion determined via $^1$H NMR.

[b] Theoretical $M_n$ calculated from the M/I ratio and conversion, assuming 100% initiation efficiency.

[c] Determined by SEC with poly(MMA) calibration.

**Figure S4.** Kinetics of MTAC-initiated Cu(0)-RDRP of MA. Experimental conditions: MA/MTAC/$Me_6$TREN = 200:1:0.5; MA/DMSO = 1:1 (v/v), 60 °C, 10 cm of activated Cu wire.

**Table S2**. Additional results obtained during the optimization of polymerization conditions for MTAC-initiated Cu-RDRP of MMA[a]

| Entry | Cat. | Solvent | Ligand (eq.) | T (°C) | Time (h) | Conv. (%)[b] | $M_n$ (theor.)[c] | $M_n$ (SEC)[d] | $Đ$[d] |
|---|---|---|---|---|---|---|---|---|---|
| 1 | Cu(0) | DMAc | PMDETA (0.2) | r.t. | 24 | 69 | 14 000 | 20 500 | 1.27 |
| 2 | Cu(0) | DMAc | PMDETA (0.5) | r.t. | 24 | 70 | 14 200 | 24 500 | 1.25 |
| 3 | Cu(0) | DMAc | PMDETA (1.0) | r.t. | 5 | 77 | 15 700 | 28 300 | 1.32 |
| 4 | Cu(0) | DMAc | PMDETA (0.2) | 85 | 6 | 87 | 17 600 | 23 600 | 1.21 |
| 5 | Cu(0) | DMAc | $Me_6TREN$ (0.2) | r.t. | 24 | 39 | 8 100 | 12 900 | 1.18 |
| 6 | Cu(0) | DMAc | $Me_6TREN$ (0.5) | r.t. | 9.5 | 73 | 14 800 | 20 900 | 1.28 |
| 7 | Cu(0) | DMAc | $Me_6TREN$ (1.0) | r.t. | 7.5 | 71 | 14 500 | 21 400 | 1.31 |
| 8 | Cu(0) | DMAc | $Me_6TREN$ (0.2) | 85 | 24 | 92 | 18 600 | 22 100 | 1.24 |
| 9 | Cu(0) | DMSO | PMDETA (0.2) | r.t. | 24 | 94 | 19 000 | 27 100 | 1.26 |
| 10 | Cu(0) | DMSO | PMDETA (0.5) | r.t. | 8.5 | 67 | 13 700 | 22 300 | 1.27 |
| 11 | Cu(0) | DMSO | PMDETA (1.0) | r.t. | 3.5 | 76 | 15 400 | 25 800 | 1.27 |
| 12 | Cu(0) | DMSO | $Me_6TREN$ (0.5) | r.t. | 7.5 | 69 | 14 000 | 23 100 | 1.23 |
| 13 | Cu(0) | DMSO | $Me_6TREN$ (1.0) | r.t. | 3.5 | 78 | 15 900 | 29 200 | 1.31 |
| 14 | Cu(0) | dioxane | PMDETA (0.2) | 85 | 24 | | *no polymerization* | | |
| 15 | Cu(0) | dioxane | PMDETA (1.0) | r.t. | 23 | 79 | 16 100 | 20 800 | 1.21 |
| 16 | Cu(0) | dioxane | $Me_6TREN$ (0.2) | 85 | 24 | 87 | 17 600 | 23 000 | 1.14 |
| 17 | Cu(0) | dioxane | $Me_6TREN$ (1.0) | r.t. | 24 | 69 | 13 900 | 28 100 | 1.62 |
| 18 | Cu(0) | dioxane | $Me_6TREN$ (1.0) | 85 | 5 | 94 | 19 000 | 29 600 | 1.28 |
| 19 | Cu(0) | toluene | $Me_6TREN$ (1.0) | 85 | 5 | 82 | 16 600 | 28 800 | 1.36 |
| 20 | Cu(0) | IPA | PMDETA (0.2) | 85 | 24 | 25 | 5 200 | 12 000 | 1.39 |
| 21 | Cu(0) | IPA | PMDETA (1.0) | 85 | 2 | 89 | 18 000 | 34 000 | 1.28 |
| 22 | Cu(0) | IPA | $Me_6TREN$ (0.2) | 85 | 24 | 15 | 3 200 | 9 700 | 1.14 |
| 23 | Cu(0) | IPA | $Me_6TREN$ (1.0) | 85 | 2 | 72 | 14 600 | 27 000 | 1.46 |
| 24 | CuBr | DMSO | PMDETA (1.0) | 85 | 24 | | *no polymerization* | | |
| 25 | CuBr | DMSO | $Me_6TREN$ (1.0) | 85 | 24 | | *no polymerization* | | |
| 26 | CuBr | DMAc | PMDETA (1.0) | 85 | 24 | | *no polymerization* | | |
| 27 | CuBr | DMAc | $Me_6TREN$ (1.0) | 85 | 24 | 2 | 520 | 2 100 | 1.30 |
| 28 | CuBr | IPA | PMDETA (1.0) | 85 | 24 | 9 | 1 900 | 4 400 | 1.12 |
| 29 | CuBr | IPA | $Me_6TREN$ (1.0) | 85 | 24 | 31 | 6 400 | 7 400 | 1.18 |
| 30 | CuBr | toluene | $Me_6TREN$ (1.0) | 85 | 22 | 92 | 18 600 | 23 500 | 1.58 |
| 31 | CuBr | dioxane | $Me_6TREN$ (1.0) | 85 | 22 | 86 | 17 400 | 20 700 | 1.69 |
| 32 | CuCl | dioxane | PMDETA (1.0) | 85 | 23 | 83 | 16 800 | 17 700 | 1.16 |

[a] Standard polymerization conditions: M/I = 200:1; monomer/solvent = 1:1 (v/v), catalyst (Cat.): 10 cm of activated copper wire in Cu(0)-RDRP, 1 eq. of Cu(I) salt in ATRP.

[b] Monomer conversion determined gravimetrically.

[c] Theoretical $M_n$ calculated from the M/I ratio and conversion, assuming 100% initiation efficiency.

[d] Determined by SEC with poly(MMA) calibration.

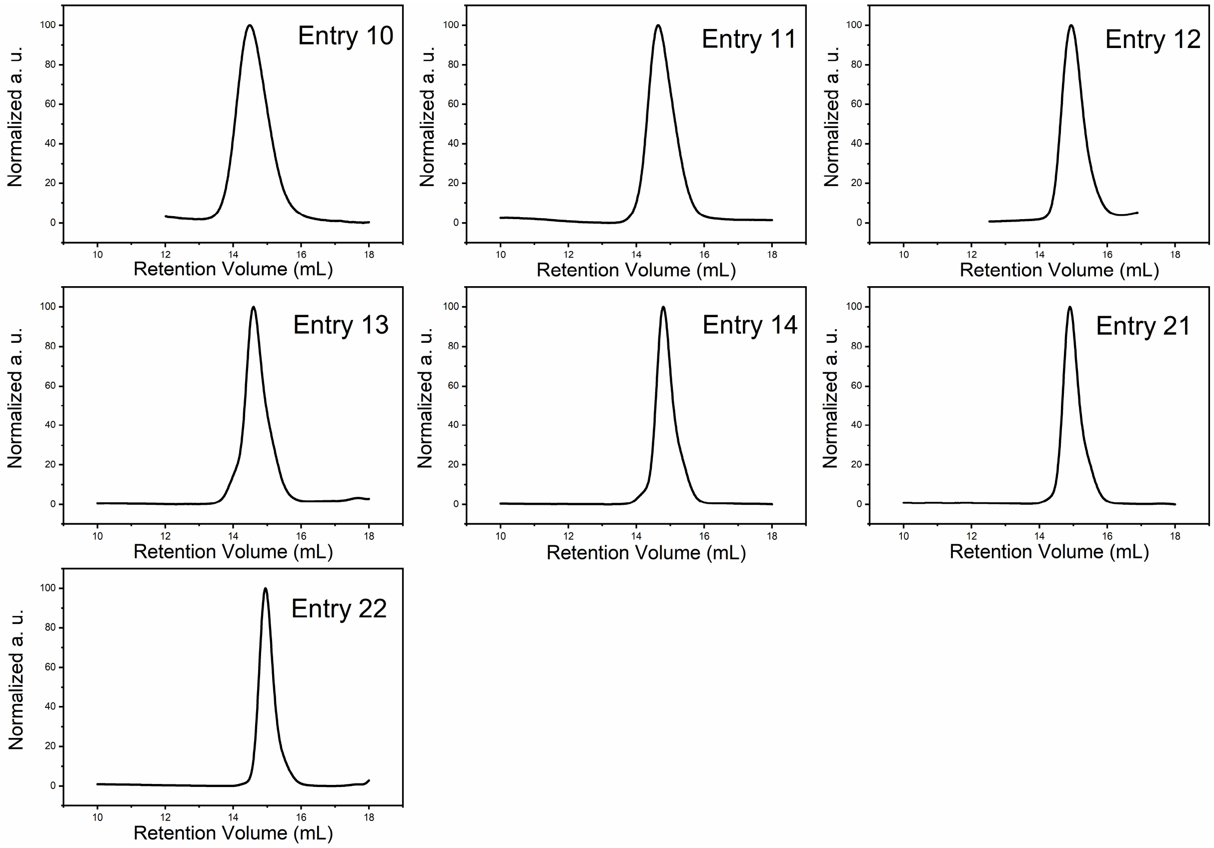


**Figure S5**. SEC elugrams of poly(MMA) prepared by MTAC-initiated Cu-RDRP at M/I = 200:1 under optimized conditions (description corresponds to Table 1).

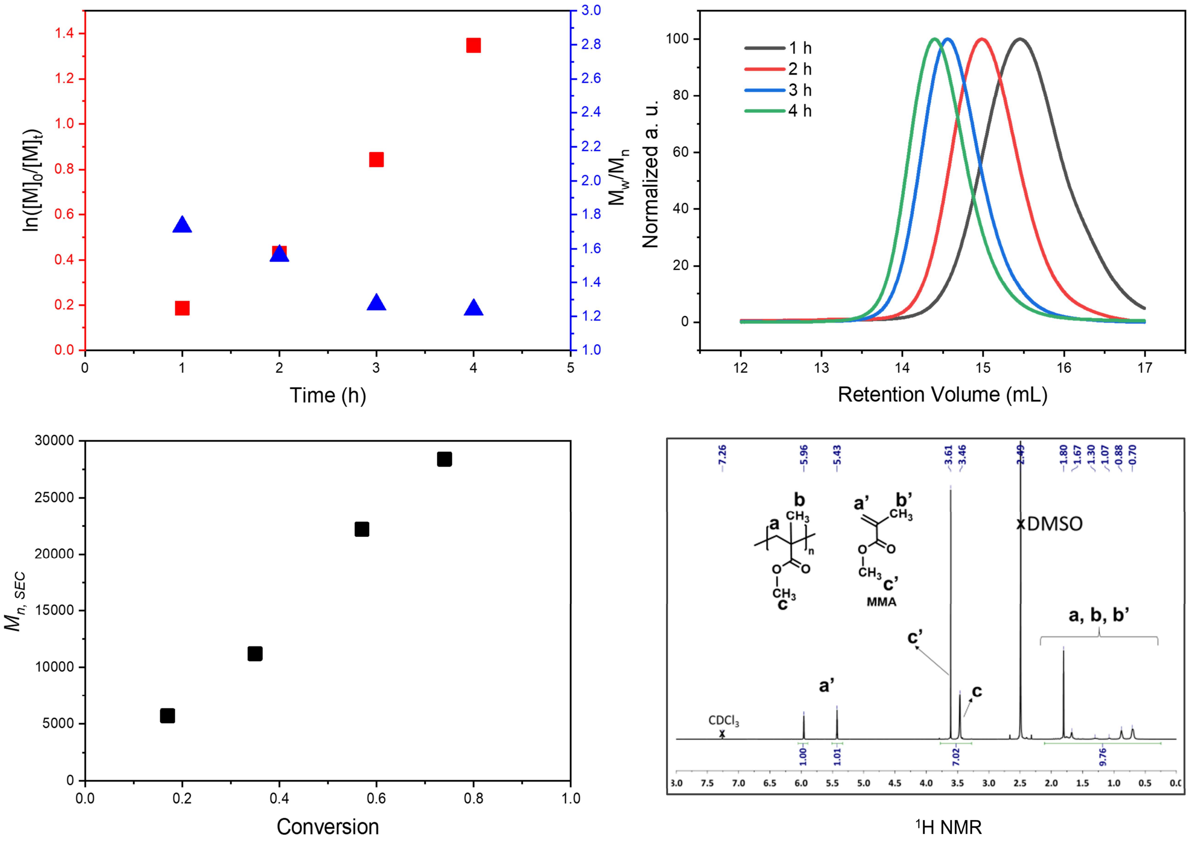


| Time (h) | Conv. (%)[a] | $M_n$ (theor.)[b] | $M_n$ (SEC)[c] | $Đ$[c] |
|---|---|---|---|---|
| 1 | 17 | 3 600 | 5 700 | 1.73 |
| 2 | 35 | 7 200 | 11 200 | 1.56 |
| 3 | 57 | 11 600 | 22 200 | 1.27 |
| 4 | 74 | 15 000 | 28 400 | 1.24 |

[a] Monomer conversion determined via $^1$H NMR.

[b] Theoretical $M_n$ calculated from the M/I ratio and conversion, assuming 100% initiation efficiency.

[c] Determined by SEC with poly(MMA) calibration.

**Figure S6.** Kinetics of MTAC-initiated Cu(0)-RDRP of MMA in DMSO. Experimental conditions: MMA/MTAC/$Me_6$TREN = 200:1:0.2; MMA/DMSO = 1:1 (v/v), 85 °C, 10 cm of activated Cu wire. Monomer conversion was determined by $^1$H NMR analysis (in $CDCl_3$) of the reaction mixture (a sample spectrum is shown together with the signal assignment; the conversion calculation was done in a similar way as for MA).

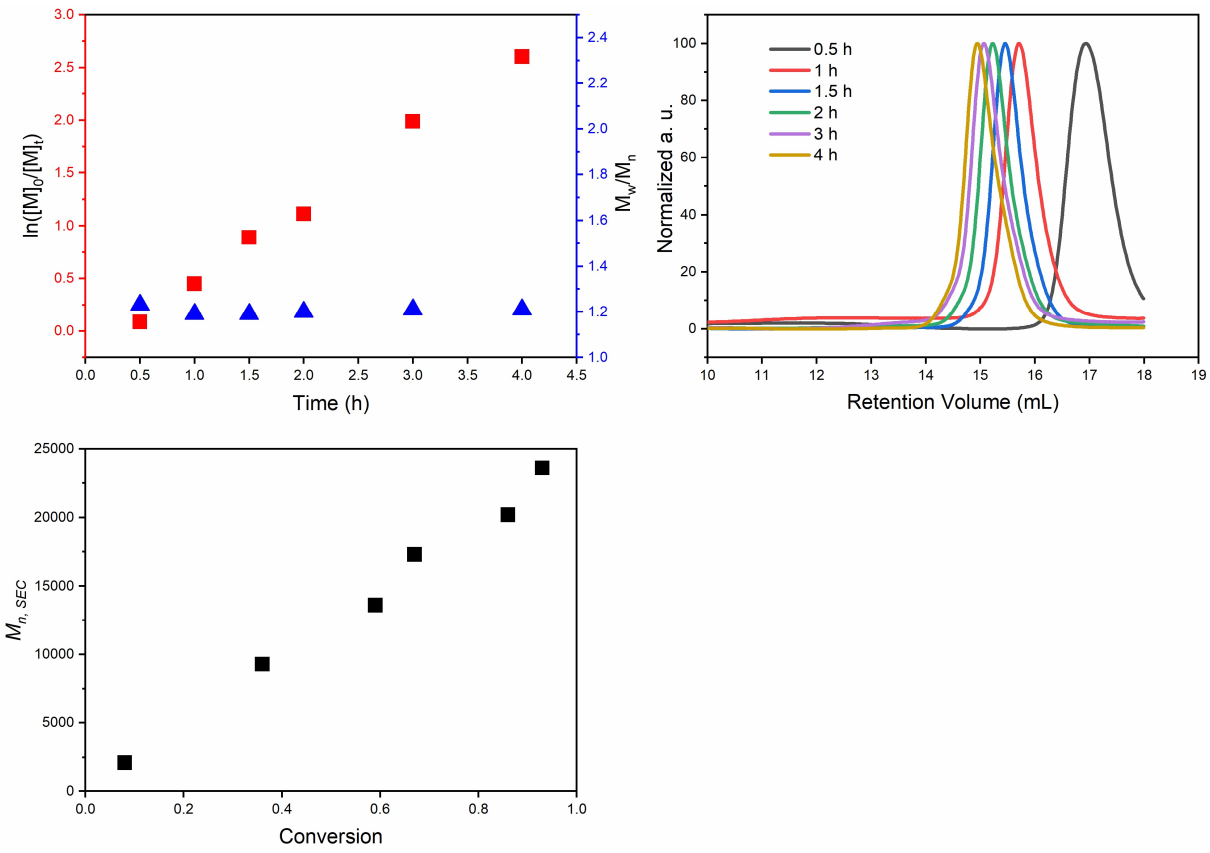


| Time (h) | Conv. (%)[a] | $M_n$ (theor.)[b] | $M_n$ (SEC)[c] | *Đ*[c] |
|---|---|---|---|---|
| 0.5 | 8 | 1 900 | 2 100 | 1.23 |
| 1 | 36 | 7 500 | 9 300 | 1.19 |
| 1.5 | 59 | 12 000 | 13 600 | 1.19 |
| 2 | 67 | 13 600 | 17 300 | 1.20 |
| 3 | 86 | 17 500 | 20 200 | 1.21 |
| 4 | 93 | 18 700 | 23 600 | 1.21 |

[a] Monomer conversion determined via $^1$H NMR.

[b] Theoretical $M_n$ calculated from the M/I ratio and conversion, assuming 100% initiation efficiency.

[c] Determined by SEC with poly(MMA) calibration.

**Figure S7.** Kinetics of MTAC-initiated Cu(0)-RDRP of MMA in dioxane. Experimental conditions: MMA/MTAC/ PMDETA = 200:1:1; MMA/dioxane = 1:1 (v/v), 85 °C, 10 cm of activated Cu wire.

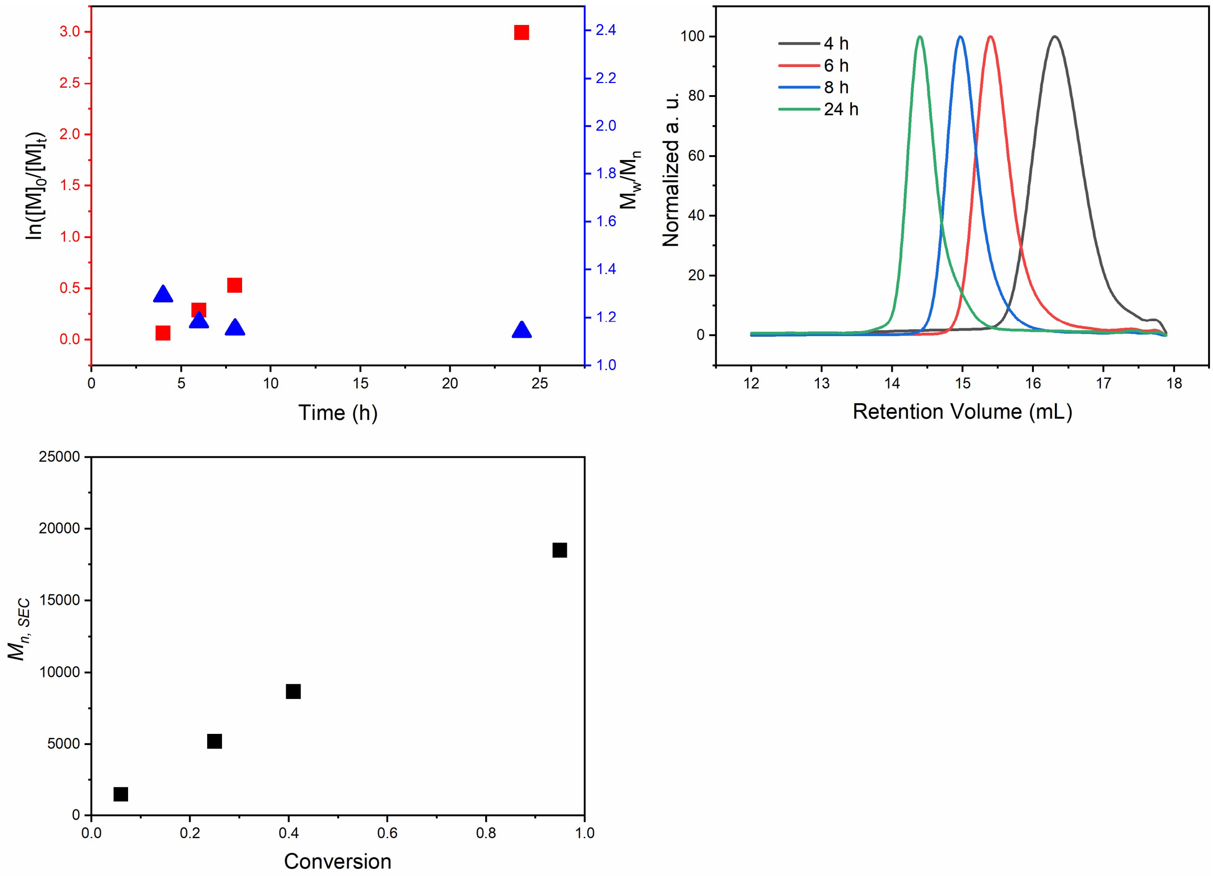


| Time (h) | Conv. (%)[a] | $M_n$ (theor.)[b] | $M_n$ (SEC)[c] | *Đ*[c] |
|---|---|---|---|---|
| 4 | 6 | 1 400 | 1 500 | 1.29 |
| 6 | 25 | 5 200 | 5 200 | 1.18 |
| 8 | 41 | 8 400 | 8 700 | 1.15 |
| 24 | 95 | 19 200 | 18 500 | 1.14 |

[a] Monomer conversion determined via $^1$H NMR.

[b] Theoretical $M_n$ calculated from the M/I ratio and conversion, assuming 100% initiation efficiency.

[c] Determined by SEC with poly(MMA) calibration.

**Figure S8**. Kinetics of MTAC-initiated ATRP of MMA in dioxane. Experimental conditions: MMA/MTAC/CuBr/ PMDETA = 200:1:1:1; MMA/dioxane = 1:1 (v/v), 85 °C.

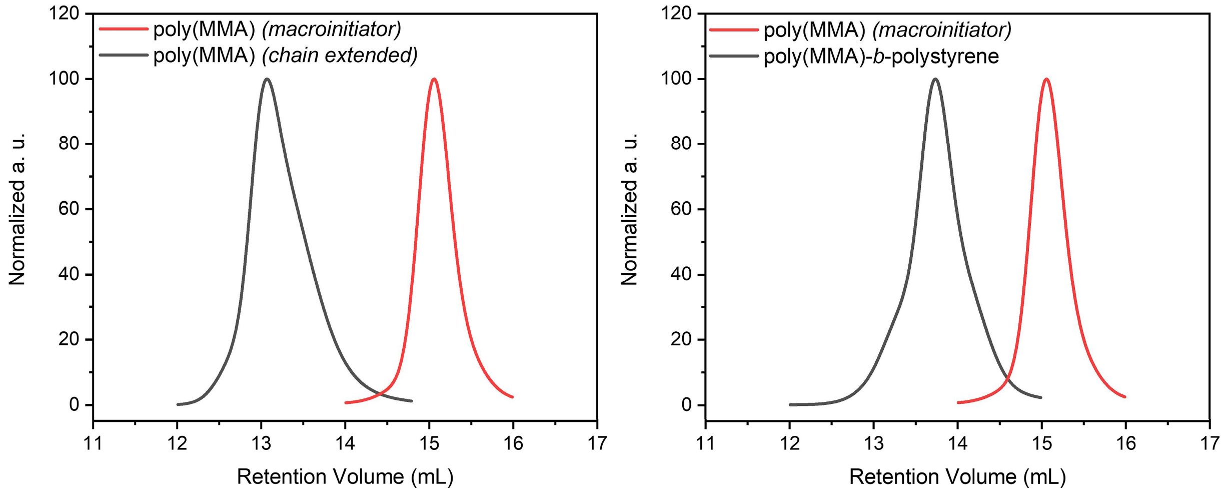


| Entry[a] | Monomer | Cat. | Solvent | Ligand (eq.) | M/I | T (°C) | Time (h) | Conv. (%)[b] | $M_n$ (theor.)[c] | $M_n$ (SEC) | *Đ* |
|---|---|---|---|---|---|---|---|---|---|---|---|
| 1 | MMA | CuBr | dioxane | PMDETA (1.0 eq.) | 400 | 85 | 2 | 73 | 38 700 | 34 400[d] | 1.25[d] |
| 2 | styrene | CuBr | - | $Me_6$TREN (1.0 eq.) | 800 | 110 | 1.5 | 26 | 31 000 | 30 000[e] | 1.26[e] |

[a] Polymerization conditions: poly(MMA) macroinitiator ($M_n$ = 9 500, Đ = 1.13; entry 24, Table 1), CuBr (1 eq.), solvent/monomer = 1:1 (v/v).

[b] Monomer conversion determined by $^1$H NMR.

[c] Theoretical $M_n$ calculated from the M/I ratio and conversion, assuming 100% initiation efficiency; the macroinitiator $M_n$ is included in the calculation.

[d] Determined by SEC with poly(MMA) calibration.

[e] Determined by SEC with polystyrene calibration.

**Figure S9.** Chain-extension study. Top: SEC elugrams of the poly(MMA) macroinitiator ($M_n$ = 9 500, Đ = 1.13) and the chain-extended poly(MMA) (left) or the poly(MMA)-*b*-polystyrene block copolymer (right). Bottom: a table with experimental data.

**Table S3**. Additional results obtained during the optimization of polymerization conditions for MTAC-initiated Cu-RDRP of styrene[a]

| Entry | Cat. | Solvent | Ligand (eq.) | T (°C) | Time (h) | Conv. (%)[b] | $M_n$ (theor.)[c] | $M_n$ (SEC)[d] | $Đ$[d] |
|---|---|---|---|---|---|---|---|---|---|
| 1 | Cu(0) | DMSO | PMDETA (0.5) | 90 | 24 | 51 | 10 800 | 27 300 | 2.30 |
| 2[e] | Cu(0) | DMSO | $Me_6$TREN (0.5) | 90 | 24 | 60 | 12 800 | 18 100 | 1.27 |
| 3[e] | Cu(0) | DMSO | $Me_6$TREN (1.0) | 90 | 24 | 81 | 17 100 | 25 800 | 1.31 |
| 4 | Cu(0) | DMAc | PMDETA (0.5) | 90 | 24 | 36 | 7 800 | 12 500 | 1.65 |
| 5[e] | Cu(0) | DMAc | $Me_6$TREN (0.5) | 90 | 24 | 56 | 12 000 | 17 300 | 1.28 |
| 6[e] | Cu(0) | DMAc | $Me_6$TREN (1.0) | 90 | 24 | 54 | 11 400 | 20 500 | 1.45 |
| 7 | Cu(0) | dioxane | PMDETA (1.0) | 90 | 24 | 75 | 15 800 | 28 000 | 1.62 |
| 8 | Cu(0) | dioxane | $Me_6$TREN (1.0) | 90 | 24 | 71 | 15 000 | 61 600 | 5.29 |
| 9 | Cu(0) | toluene | PMDETA (0.5) | 90 | 24 | | *no polymerization* | | |
| 10[e] | Cu(0) | toluene | $Me_6$TREN (0.5) | 90 | 24 | 49 | 10 400 | 13 000 | 1.38 |
| 11[e] | Cu(0) | toluene | $Me_6$TREN (1.0) | 90 | 24 | 65 | 13 700 | 19 000 | 1.44 |
| 12 | Cu(0) | toluene | $Me_6$TREN (0.2) | 90 | 48 | 57 | 12 000 | 14 700 | 1.21 |
| 13[e] | Cu(0) | - | PMDETA (0.5) | 90 | 9 | 15 | 6 300 | 9 200 | 2.01 |
| 14[e] | Cu(0) | - | $Me_6$TREN (0.5) | 90 | 24 | 67 | 28 300 | 31 300 | 1.25 |
| 15[e] | Cu(0) | - | $Me_6$TREN (1.0) | 90 | 24 | 89 | 37 200 | 42 500 | 1.36 |
| 16[e] | Cu(0) | - | $Me_6$TREN (0.2) | 90 | 24 | 37 | 15 600 | 16 800 | 1.24 |
| 17 | CuBr | DMSO | PMDETA (1.0) | 90 | 24 | | *no polymerization* | | |
| 18 | CuBr | DMSO | $Me_6$TREN (1.0) | 90 | 24 | | *no polymerization* | | |
| 19 | CuBr | DMAc | PMDETA (1.0) | 90 | 24 | | *no polymerization* | | |
| 20 | CuBr | DMAc | $Me_6$TREN (1.0) | 90 | 24 | | *no polymerization* | | |
| 21 | CuBr | IPA | PMDETA (1.0) | 90 | 24 | | *no polymerization* | | |
| 22 | CuBr | IPA | $Me_6$TREN (1.0) | 90 | 24 | | *no polymerization* | | |
| 23 | CuBr | dioxane | PMDETA (1.0) | 90 | 24 | | *no polymerization* | | |
| 24 | CuBr | dioxane | $Me_6$TREN (1.0) | 90 | 24 | 15 | 3 300 | 4 200 | 1.29 |
| 25 | CuBr | toluene | PMDETA (1.0) | 90 | 24 | | *no polymerization* | | |
| 26 | CuBr | toluene | $Me_6$TREN (1.0) | 90 | 24 | 42 | 8 900 | 10 000 | 1.23 |
| 27 | CuBr | - | PMDETA (1.0) | 90 | 24 | 12 | 5 200 | 5 200 | 1.38 |
| 28 | CuBr | - | $Me_6$TREN (1.0) | 90 | 22 | 87 | 36 300 | 45 000 | 1.20 |
| 29 | CuCl | - | $Me_6$TREN (1.0) | 90 | 24 | 10 | 4 200 | 4 600 | 1.16 |
| 30[f] | CuBr | - | $Me_6$TREN (1.0) | 110 | 3 | 91 | 5 000 | 5 900 | 1.61 |

[a] Standard polymerization conditions: M/I = 200:1 in solvents and 400:1 in bulk; monomer/solvent = 1:1 (v/v), catalyst (Cat.): 10 cm of activated copper wire in Cu(0)-RDRP, 1 eq. of Cu(I) salt in ATRP.

[b] Monomer conversion determined gravimetrically.

[c] Theoretical $M_n$ calculated from the M/I ratio and conversion, assuming 100% initiation efficiency.

[d] Determined by SEC with polystyrene calibration.

[e] Gel formation on Cu wire was observed.

[f] Bulk polymerization at M/I = 50:1

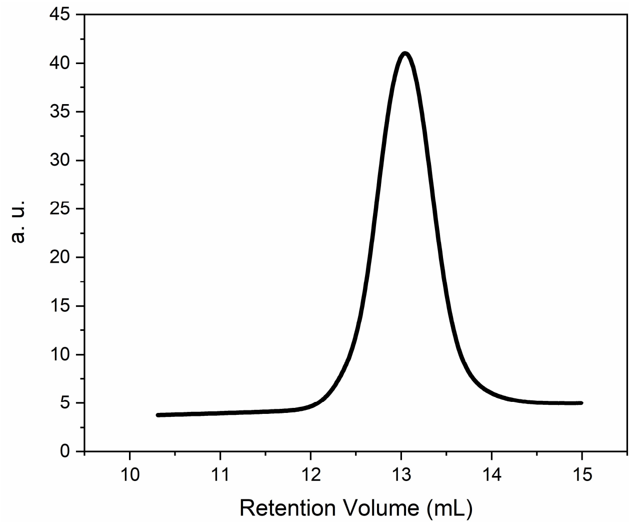

**Figure S10**. SEC elugram for polystyrene prepared by MTAC-initiated Cu(0)-RDRP in toluene (Entry 26, Table 1).

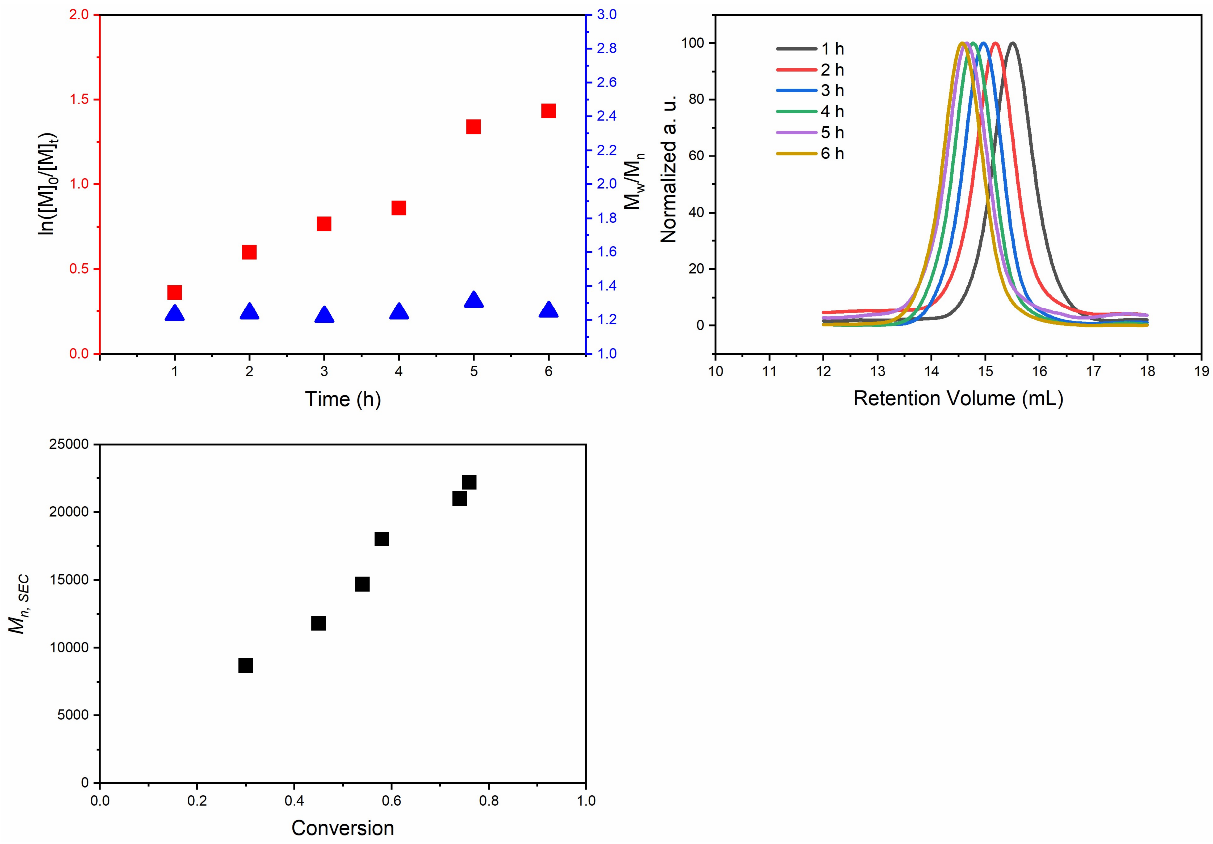


| Time (h) | Conv. (%)[a] | $M_n$ (theor.)[b] | $M_n$ (SEC)[c] | Đ[c] |
|---|---|---|---|---|
| 1 | 30 | 6 500 | 8 700 | 1.23 |
| 2 | 45 | 9 600 | 11 800 | 1.24 |
| 3 | 54 | 11 400 | 14 700 | 1.22 |
| 4 | 58 | 12 200 | 18 000 | 1.24 |
| 5 | 74 | 15 600 | 21 000 | 1.31 |
| 6 | 76 | 16 100 | 22 200 | 1.25 |

[a] Monomer conversion determined gravimetrically.

[b] Theoretical $M_n$ calculated from the M/I ratio and conversion, assuming 100% initiation efficiency.

[c] Determined by SEC with polystyrene calibration.

**Figure S11**. Kinetics of MTAC-initiated ATRP of styrene performed in bulk. Experimental conditions: styrene/MTAC/CuBr/ $Me_6TREN$ = 200:1:1:1; 110 °C.

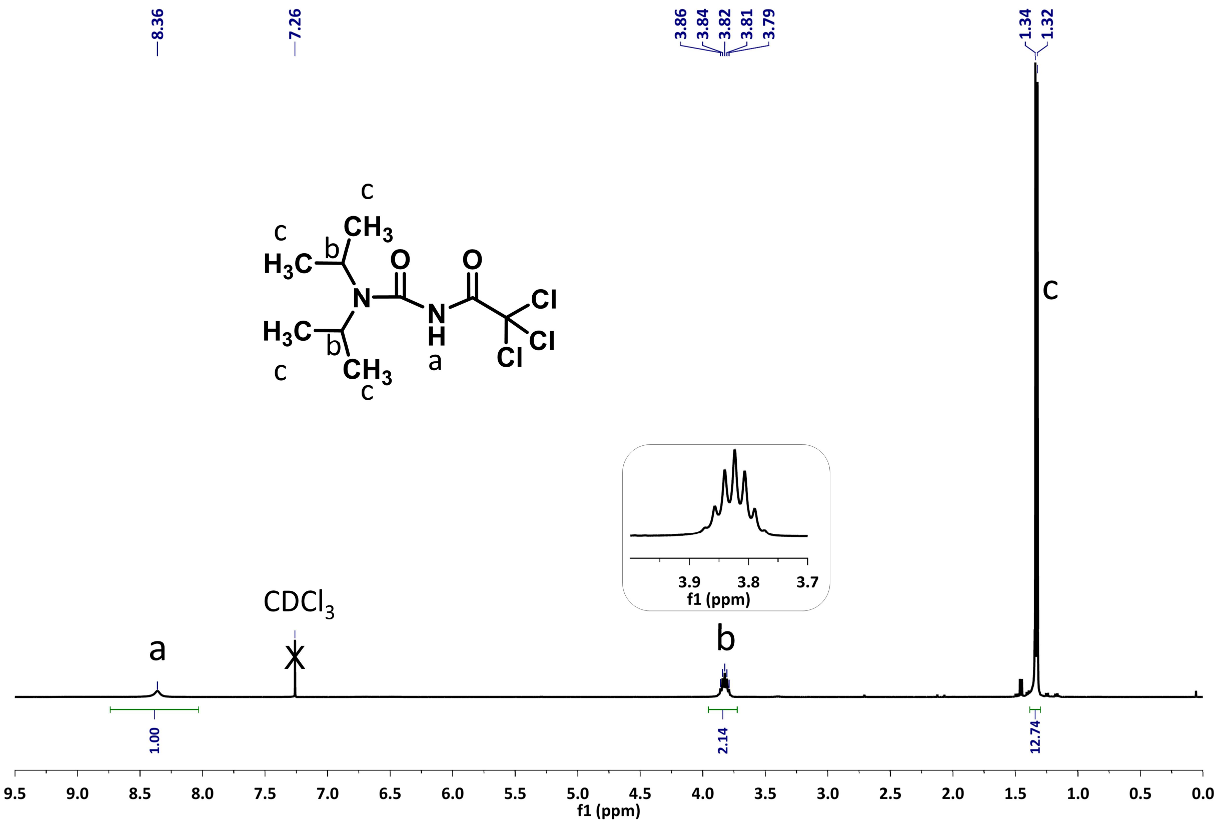


**Figure S12**. $^{1}H$ NMR ($CDCl_3$) spectrum of the synthesized 1,1-diisopropyl-3-(2,2,2-trichloroacetyl)-urea (DTAU).

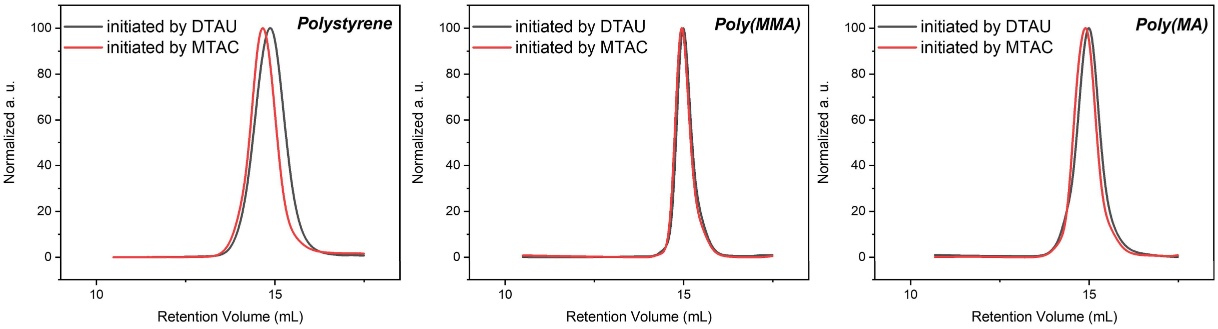


| Entry[a] | Monomer | Cat. | Solvent | Ligand (eq.) | T (°C) | Time (h) | Conv. (%)[b] | $M_n$ (theor.)[c] | $M_n$ (SEC)[d] | Đ[d] |
|---|---|---|---|---|---|---|---|---|---|---|
| 1 | styrene | CuBr | - | $Me_6TREN$ (1.0 eq.) | 110 | 6 | 74 | 15 700 | 18 200 | 1.30 |
| 2 | MMA | CuBr | dioxane | PMDETA (1.0 eq.) | 85 | 24 | 75 | 15 300 | 18 900 | 1.10 |
| 3 | MA | Cu(0) | DMSO | $Me_6TREN$ (0.5 eq.) | 60 | 5 | 96 | 16 600 | 21 800 | 1.30 |

[a] Standard polymerization conditions: M/I = 200:1; catalyst (Cat.): 10 cm of activated copper wire in Cu(0)-RDRP, CuBr (1 eq.) in ATRP; monomer/solvent = 1:1 (v/v).

[b] Monomer conversion determined gravimetrically (styrene, MMA) or by $^1H$ NMR (MA).

[c] Theoretical $M_n$ calculated from the M/I ratio and conversion, assuming 100% initiation efficiency.

[d] Determined by SEC with polystyrene (for polystyrene) or poly(MMA) calibration (for poly(MA) and poly(MMA)).

**Figure S13.** Comparison of MTAC- and DTAU-initiated Cu-RDRP of styrene, MMA, and MA. Top: SEC elugrams of the polymers synthesized under identical conditions using MTAC and DTAU initiators. Bottom: experimental data for the DTAU-initiated Cu-RDRP of styrene, MMA, and MA.

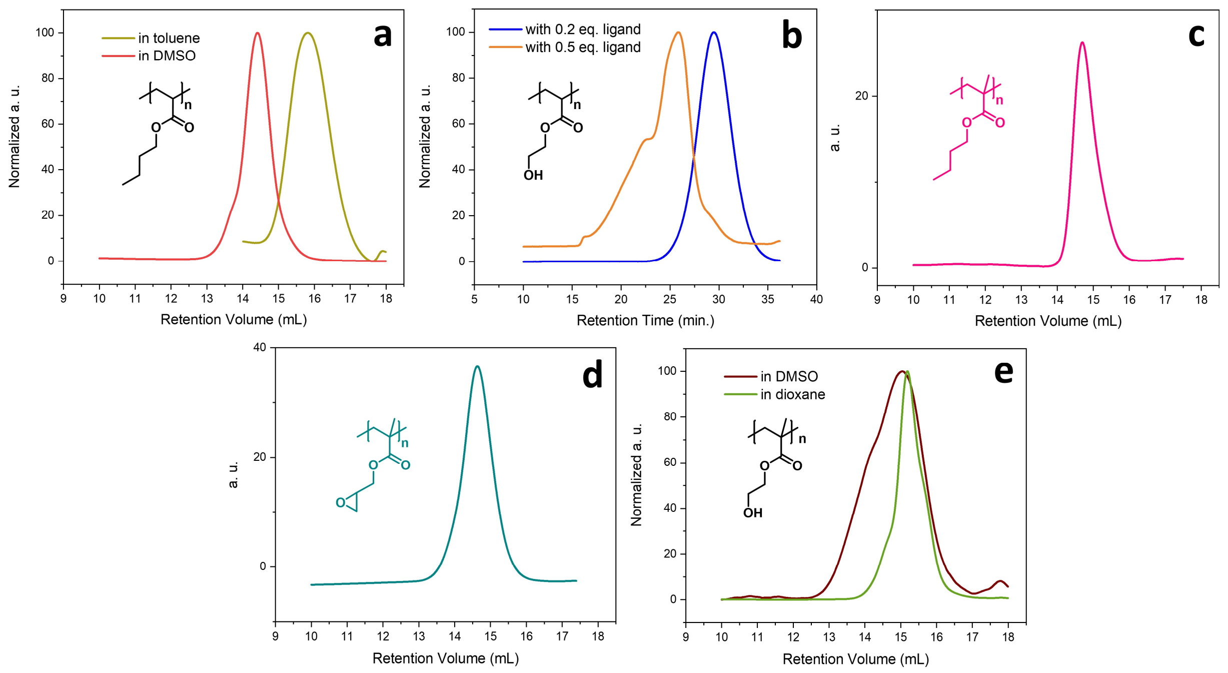


**Figure S14.** SEC elugrams of a) poly(BA), b) poly(HEA), c) poly(BMA), d) poly(GMA), and e) poly(HEMA) prepared using the previously optimized polymerization conditions (with or without modification). Experimental conditions and product characteristics are provided in Table 2.

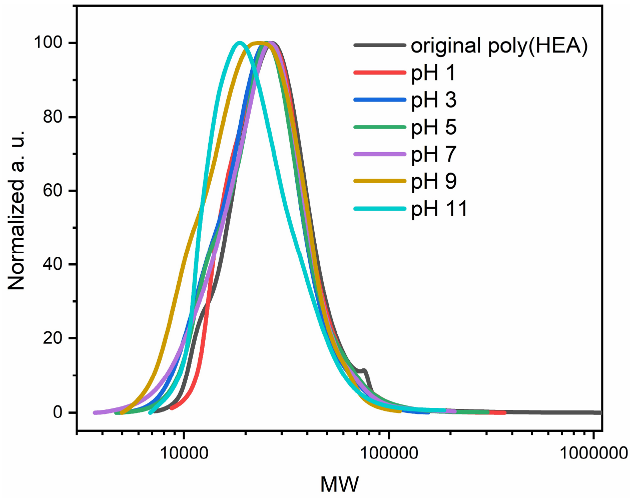


| Buffer pH | $M_n$ | $M_p$ | Đ | dn/dc (mL/g) |
|---|---|---|---|---|
| - | 24 300 | 27 100 | 1.28 | 0.072 |
| 1 | 24 700 | 27 000 | 1.21 | 0.073 |
| 3 | 21 300 | 25 000 | 1.26 | 0.075 |
| 5 | 22 200 | 25 400 | 1.28 | 0.075 |
| 7 | 21 000 | 25 600 | 1.29 | 0.074 |
| 9 | 19 000 | 22 800 | 1.28 | 0.070 |
| 11 | 20 500 | 20 700 | 1.21 | 0.060 |

**Figure S15.** Study of the hydrolytic stability of the in-chain carbamate linkers in ETAC-initiated poly(HEA). *Left:* MWDs of the ETAC-initiated poly(HEA) before and after exposure to buffers of different pH for 24 h at 37 °C. The data were obtained for isolated samples by TD-SEC.

*Right:* Table with the experimental results (data for the starting poly(HEA) are provided in the first line). Experimental conditions: polymer concentration 5 mg/mL, 24 h at 37°C; MWs of the isolated polymers were determined by TD-SEC in DMAc/LiBr. The dn/dc values were determined by the OMNISEC software assuming 100% sample recovery.

Additional discussion: In order to gain a preliminary insight into the hydrolytic stability of the TAI-derived carbamate linker employed prevalently in this work, we prepared a model poly(HEA) polymer through Cu(0)-RDRP of HEA initiated with ethane-1,2-diyl bis((2,2,2-trichloroacetyl)carbamate) (ETAC) using the HEA/ETAC/$Me_6$TREN ratio of 400:1:0.2 in DMSO at 60 °C. ETAC was synthesized by the reaction of ethylene glycol with an excess of TAI and used without further purification; its $^1$H NMR spectrum is provided in **Figure S16**. Samples of the prepared poly(HEA) of $M_n$ = 24 300, Đ = 1.28, featuring the 6-arm star architecture (based on the assumed TAG trifunctionality), was then dissolved in six different buffers, covering the pH range of 1 to 11, and maintained at 37 °C for 24 h. The polymer MW was evaluated by TD-SEC, expecting that the cleavage of the carbamate linker would result into halving of the polymer MW. As can be seen from the MW distributions (MWDs) displayed in **Figure S15**, the carbamate linker has shown to be considerably resistant to hydrolysis in the whole studied pH range. Only at pH 9 and 11, a noticeable change to MWDs could be observed; however, as is apparent from the $M_n$ and, particularly, $M_p$ (main fraction MW) values reported in **Figure S15**, the actual shift in MWs is rather small, inconsistent with extensive hydrolysis of the carbamate linkers. Instead, we ascribe this small MW shift to partial hydrolysis of poly(HEA) ester side groups at alkaline pH. This assumption is supported by the observed decrease in the determined polymer dn/dc from 0.072 mL/g (original poly(HEA)) to 0.060 mL/g (the pH 11 sample), which indicates a change in polymer composition (**Figure S15**). On the whole, this preliminary investigation points to the considerable hydrolytic stability of the in-chain TAI-derived carbamate linker in a wide pH range as compared to low-MW carbamates,[4] which could be ascribed to the steric crowding resulting from the polymeric chains growing from the nearby multifunctional initiation sites.

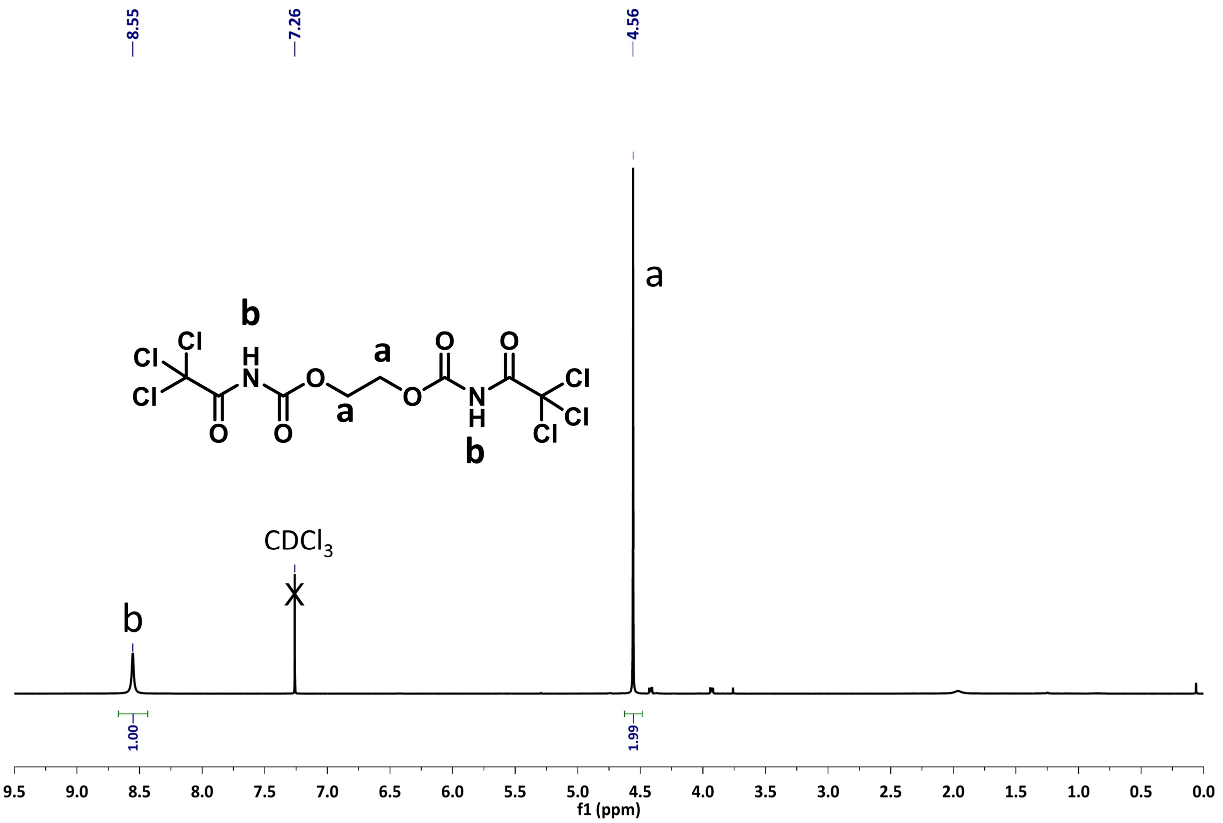


**Figure S16.** $^1$H NMR ($CDCl_3$) of ethane-1,2-diyl bis((2,2,2-trichloroacetyl)carbamate) (ETAC).

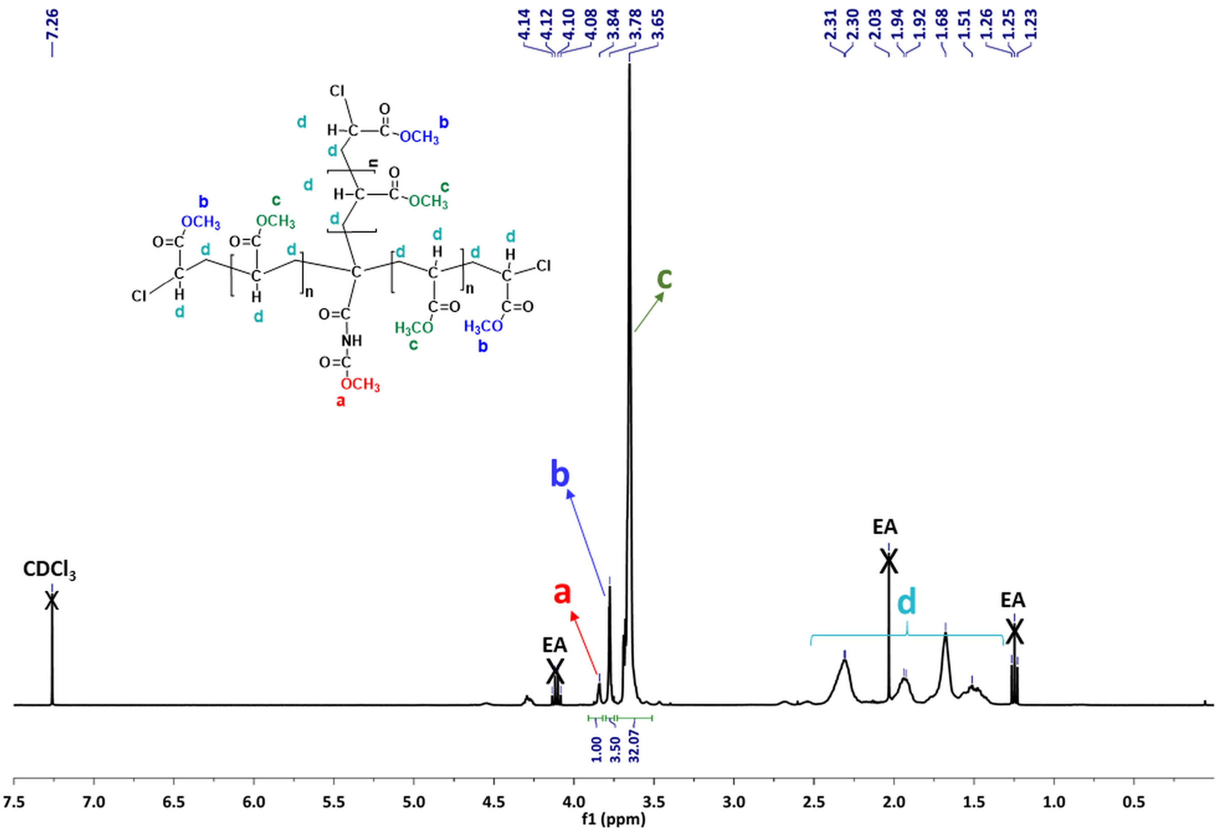



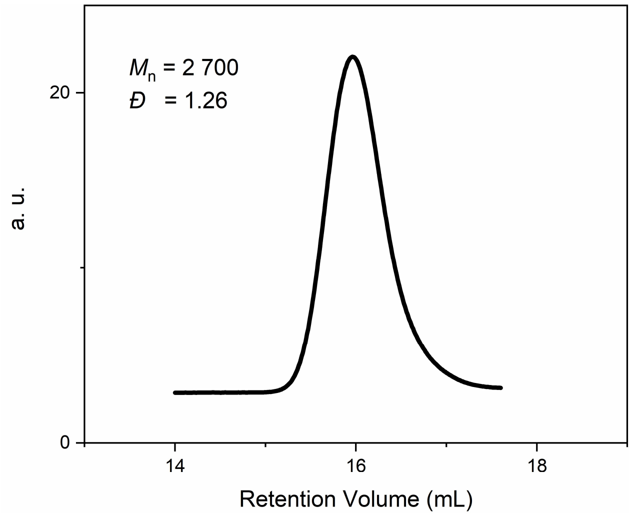



**Figure S17.** $^1$H NMR study of the MTAC initiator functionality in Cu-RDRP of MA. Top: $^1$H NMR ($CDCl_3$) of the isolated model poly(MA). Bottom: SEC elugram of the model poly(MA) (MW determined using poly(MMA) calibration). Polymerization conditions: Cu(0)-RDRP, DMSO, $Me_6$TREN 0.5 eq., 2.5 h, 60°C, 42 % conversion; polymer isolation: ethyl acetate-diluted polymerization mixture was extracted with water 3x, salted out with brine, dried with $MgSO_4$, and evaporated; the obtained solids were dried in vacuum.

<u>Calculation of initiator functionality (IF) from NMR signal intensity (I):</u>

*1st approach:* IF = $I_b/I_a$ = 3.5/1 = **3.5**

*2nd approach:* $M_{n(single\ chain)} = [(I_c + I_b)/ I_b] \times M_{MA}$ = [(32.07+3.50)/3.50] × 86.09 = 875

IF = $M_{n(SEC)}/M_{n(single\ chain)}$ = 2700/875 = **3.1**

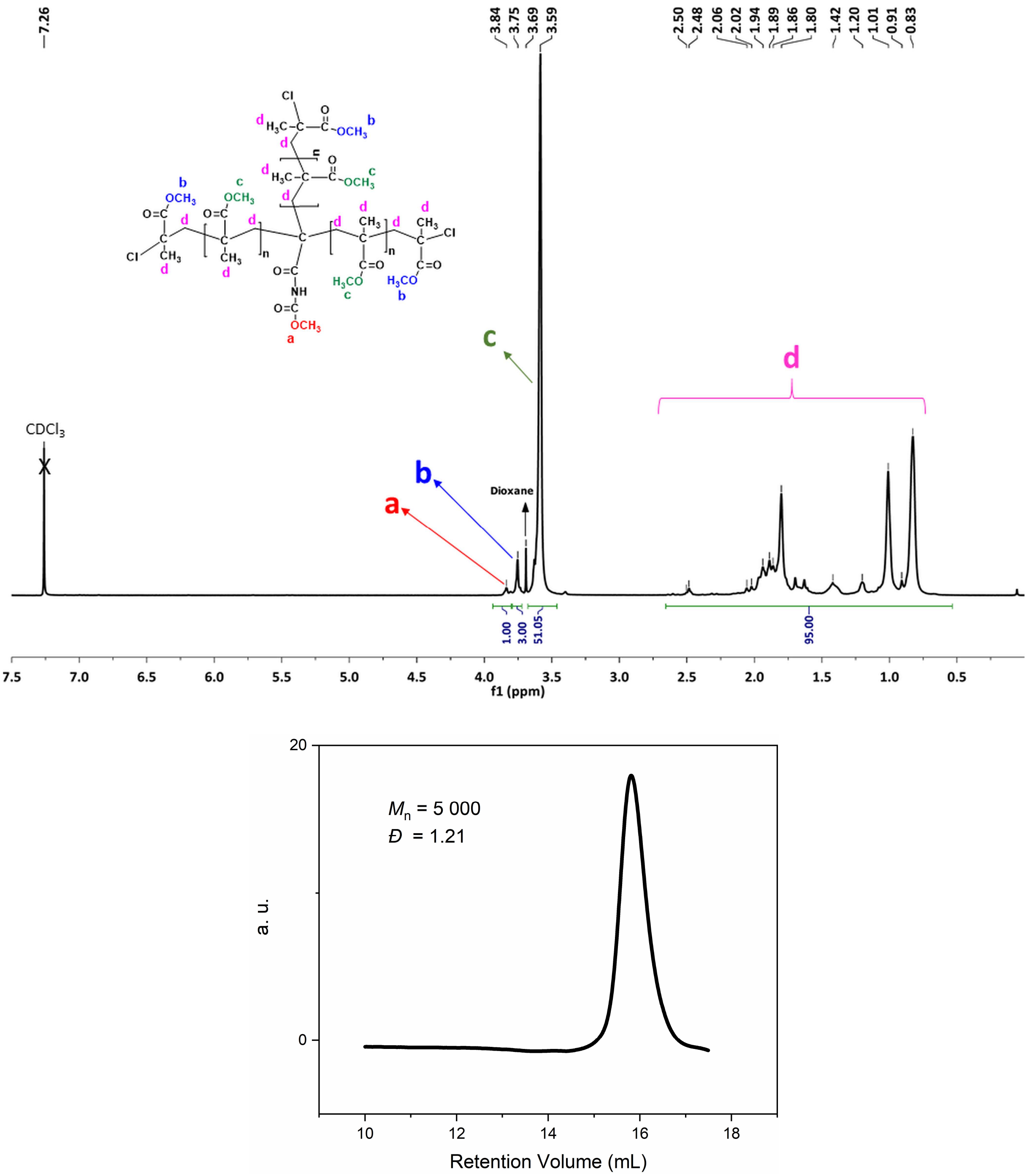


**Figure S18.** $^{1}$H NMR study of the MTAC initiator functionality in Cu-RDRP of MMA. Top: $^{1}$H NMR ($CDCl_3$) of the isolated model poly(MMA). Bottom: SEC elugram of the model poly(MMA) (MW determined using poly(MMA) calibration). Polymerization conditions are provided in entry 23, Table 1; the polymer was isolated via precipitation in MeOH/water (1:1 v/v) and dried in vacuum.

Calculation of IF from NMR signal intensity (I):

*1st approach:* IF = $I_b/I_a$ = 3/1 = **3**

*2nd approach:* $M_{n(\text{single chain})} = [(I_c+I_b)/I_b] \times M_{MMA}$ = [(51.05+3.00)/3.00] × 100.12 = 1804

IF = $M_{n(\text{SEC})}/M_{n(\text{single chain})}$ = 5000/1804 = **2.8**

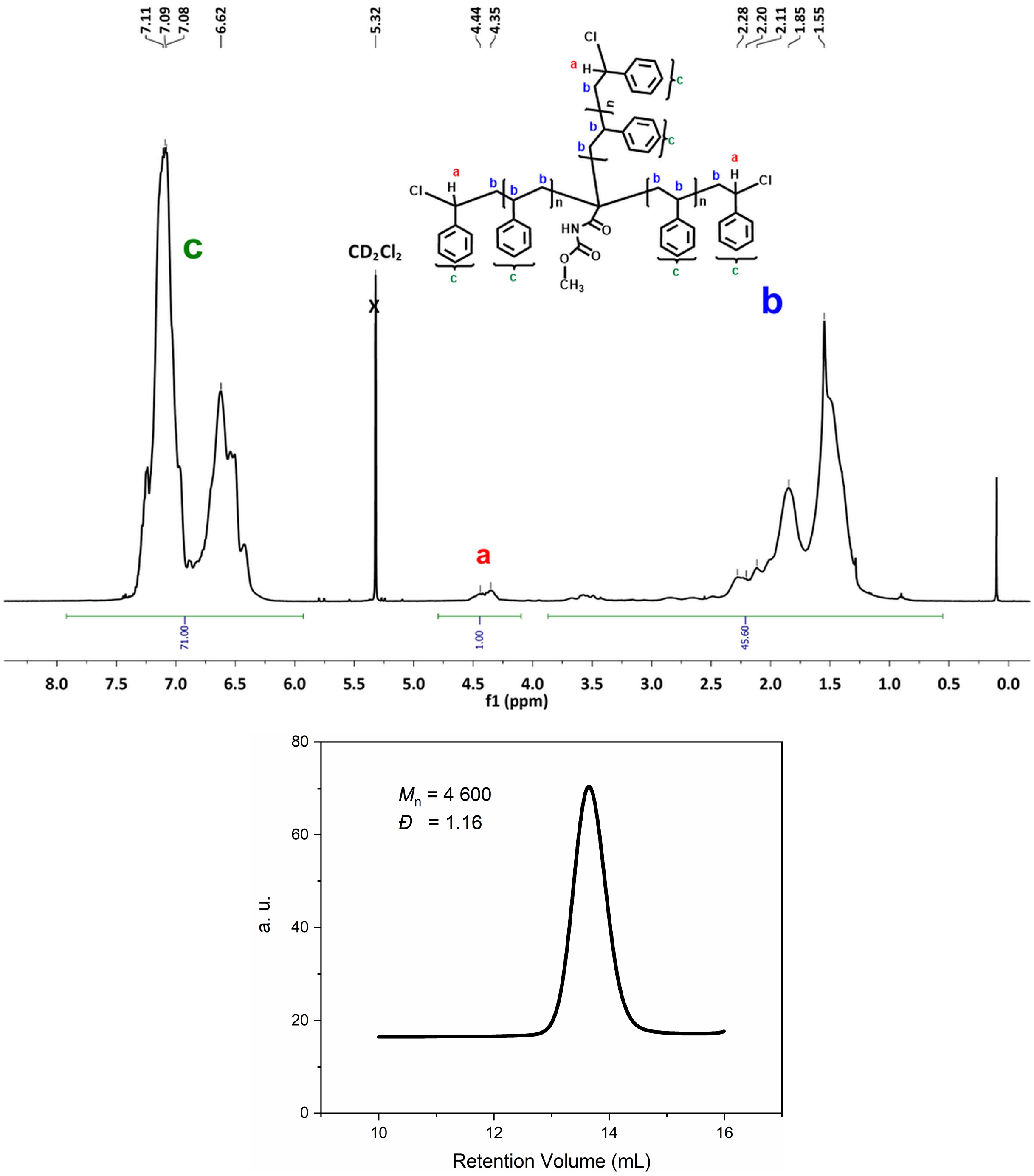


**Figure S19.** $^{1}$H NMR study of the MTAC initiator functionality in Cu-RDRP of styrene. Top: $^{1}$H NMR ($CD_2Cl_2$) of the isolated model polystyrene. Bottom: SEC elugram of the model polystyrene (MW determined using polystyrene calibration). Polymerization conditions are provided in entry 29, Table S3; the polymer was isolated via precipitation in MeOH and dried in vacuum.

Calculation of IF from NMR signal intensity (I):

$M_{n(\text{single chain})} = [(I_c/5)/I_a] \times M_{St} = [(71.00/5)/1.00] \times 104.15 = 1479$

IF = $M_{n(\text{SEC})}/M_{n(\text{single chain})}$ = 4600/1479 = **3.1**

*(Note: This is the calculation by the 2nd approach; for all the studied polymers, the IF value calculated in this way is inherently slightly underestimated due to the underestimation of the $M_n$ value determined by SEC with relative calibration that does not reflect the branching-related decrease in polymer hydrodynamic volume).*

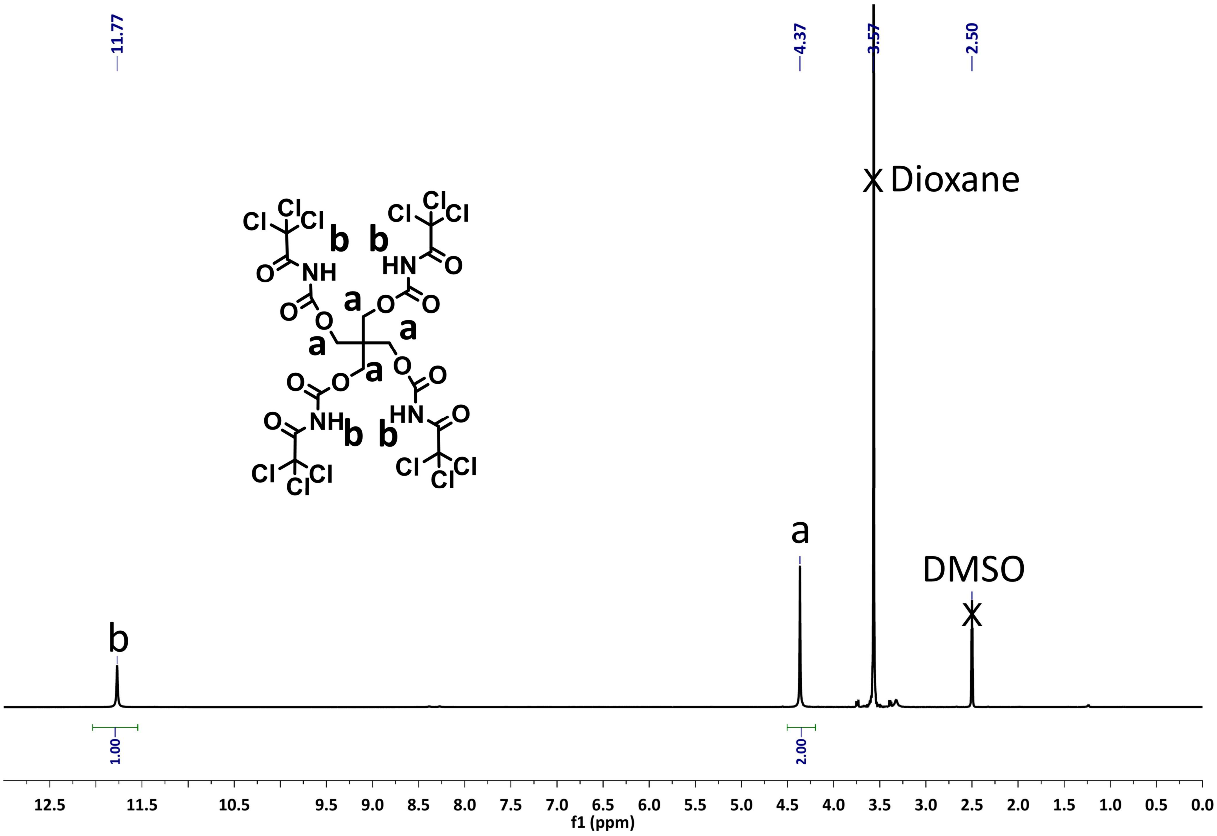


**Figure S20.** $^1$H NMR (DMSO-*d6*) of pentaerythritol tetrakis((2,2,2-trichloroacetyl)carbamate) (PTAC)

**Table S4.** Synthesis of star polymers via PTAC-initiated Cu-RDRP of MMA, styrene, and MA[a]

| Monomer | Stage | Time (h) | Conv. (%)[b] | $M_n$ (theor.)[c] | $M_n$ (SEC)[d] | $Đ$[d] |
|---|---|---|---|---|---|---|
| MMA | original star polymer | 6 | 92 | 37 700 | 48 600 | 1.21 |
| | hydrolyzed segments | - | - | 9 400 | 14 900 | 1.08 |
| styrene | original star polymer | 6 | 50 | 84 200 | 74 700 | 1.69 |
| | hydrolyzed segments | - | - | 21 100 | 29 000 | 1.14 |
| MA | original star polymer | 4 | 92 | 32 000 | 32 600 | 1.48 |

[a] Polymerization conditions: MMA polymerization - MMA/PTAC/CuBr/PMDETA = 400:1:1:1, MMA/dioxane = 1:1 (v/v), 85 °C; styrene polymerization - styrene/PTAC/CuBr/$Me_6$Tren = 1600:1:1:1, in bulk, 110 °C; MA polymerization - MA/PTAC/$Me_6$TREN = 400:1:0.5, MA/DMSO = 1:1 (v/v), copper wire 10 cm, 60 °C. Alkaline hydrolysis was conducted according to a literature procedure.[2]

[b] Monomer conversion was determined gravimetrically (MMA and styrene) or through a $^1$H NMR analysis (MA).

[c] Theoretical $M_n$ calculated from the M/I ratio and conversion, assuming 100% initiation efficiency.

[d] Determined by TD-SEC.

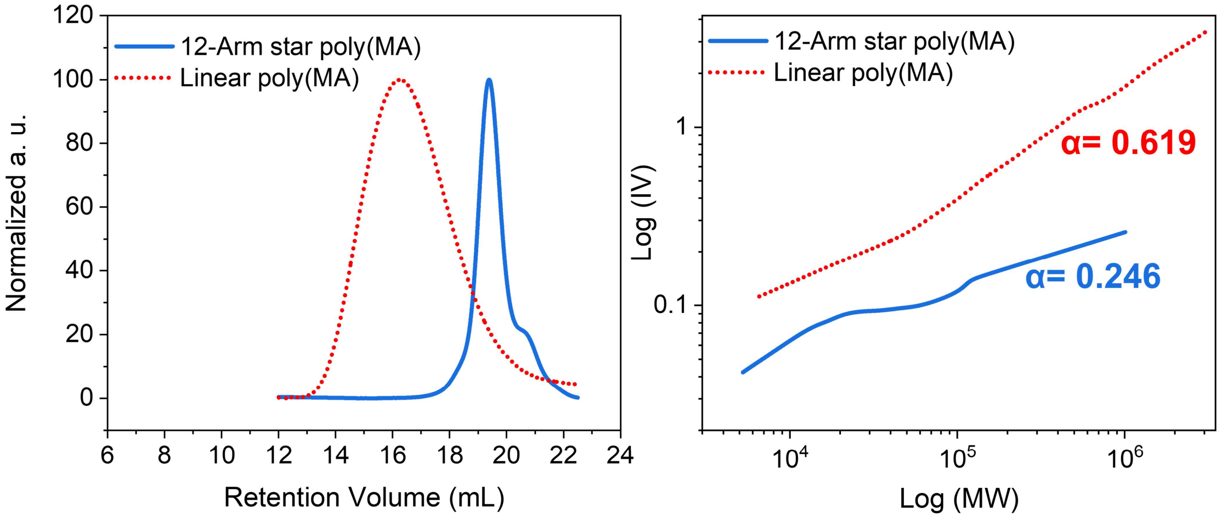


**Figure S21.** TD-SEC analysis of multi-arm star poly(MA) based on a pentaerythritol core and synthesized via the TAI strategy. Left: SEC elugrams (RI traces), right: corresponding M-H plots. Data for a broad linear poly(MA) synthesized through free-radical polymerization are shown for comparison.

**Table S5**. Experimental results of the d*e novo* one-pot synthesis of the poly(HEMA-*co*-MMA)-*graft*-poly(MMA) “star-on-star” graft copolymer[a]

| Stage | Conv. (%)[b] | $M_n$ (theor.) | $M_n$ (SEC)[e] | $Đ$[e] |
|---|---|---|---|---|
| poly(HEMA-*co*-MMA) | 99 | 10 800[c] | 23 400 | 1.23 |
| poly(HEMA-*co*-MMA)-*graft*-poly(MMA) | 30 | 288 300[d] | 328 000 | 1.95 |
| removed poly(MMA) grafts | - | 12 000[c] | 16 200 | 1.05 |

[a] Polymerization conditions: MMA/HEMA copolymerization: MMA/HEMA/MTAC/PMDETA = 80/20/1/1, 4 cm of activated copper wire, monomers/solvent = 1:1 (v/v), 85 °C, 3 h; grafting: MMA/TAI/PMDETA = 400:1:1, 4 cm of activated copper wire from the previous step, monomer/solvent = 3:1 (v/v), 85 °C, 2 h.

[b] Monomer conversion as determined by $^{1}$H NMR.

[c] Theoretical $M_n$ calculated from the M/I ratio and conversion, assuming 100% initiation efficiency.

[d] Theoretical $M_n$ calculated from the $M_n$(SEC) and the known composition of the macroinitiator, the average number of initiating sites per one macroinitiator chain, and the determined MW of the macroinitiator and grafts.

[e] Determined by TD-SEC.

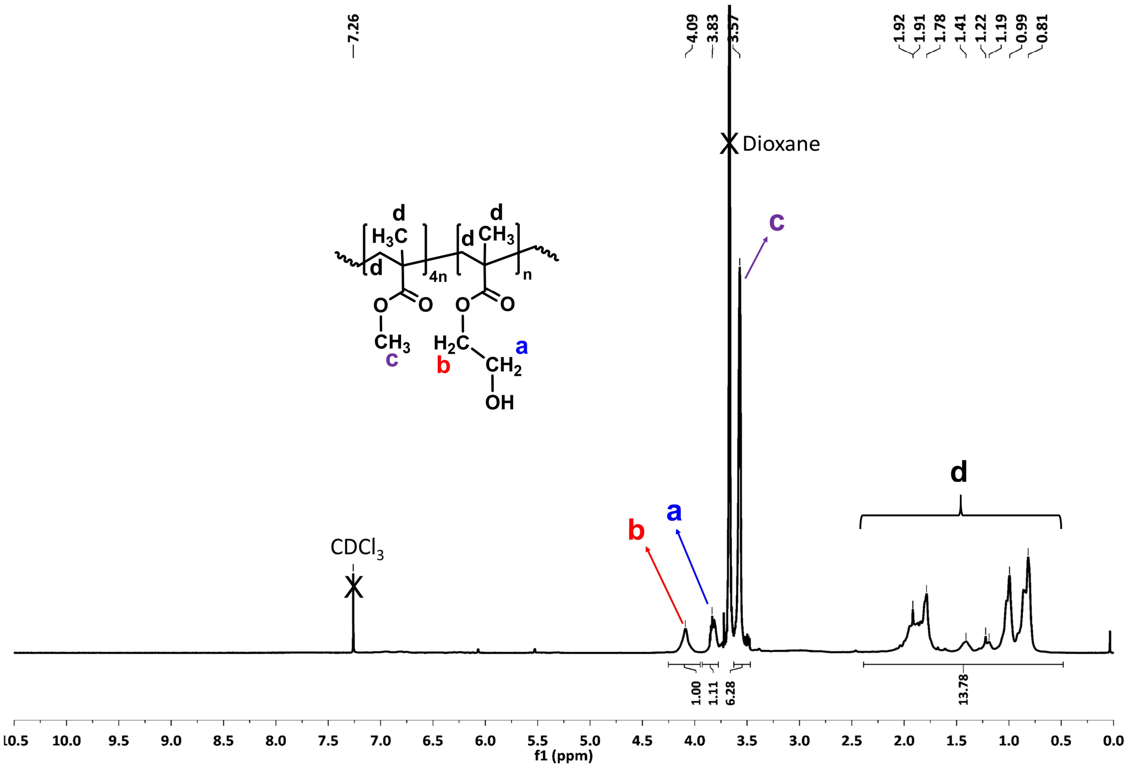


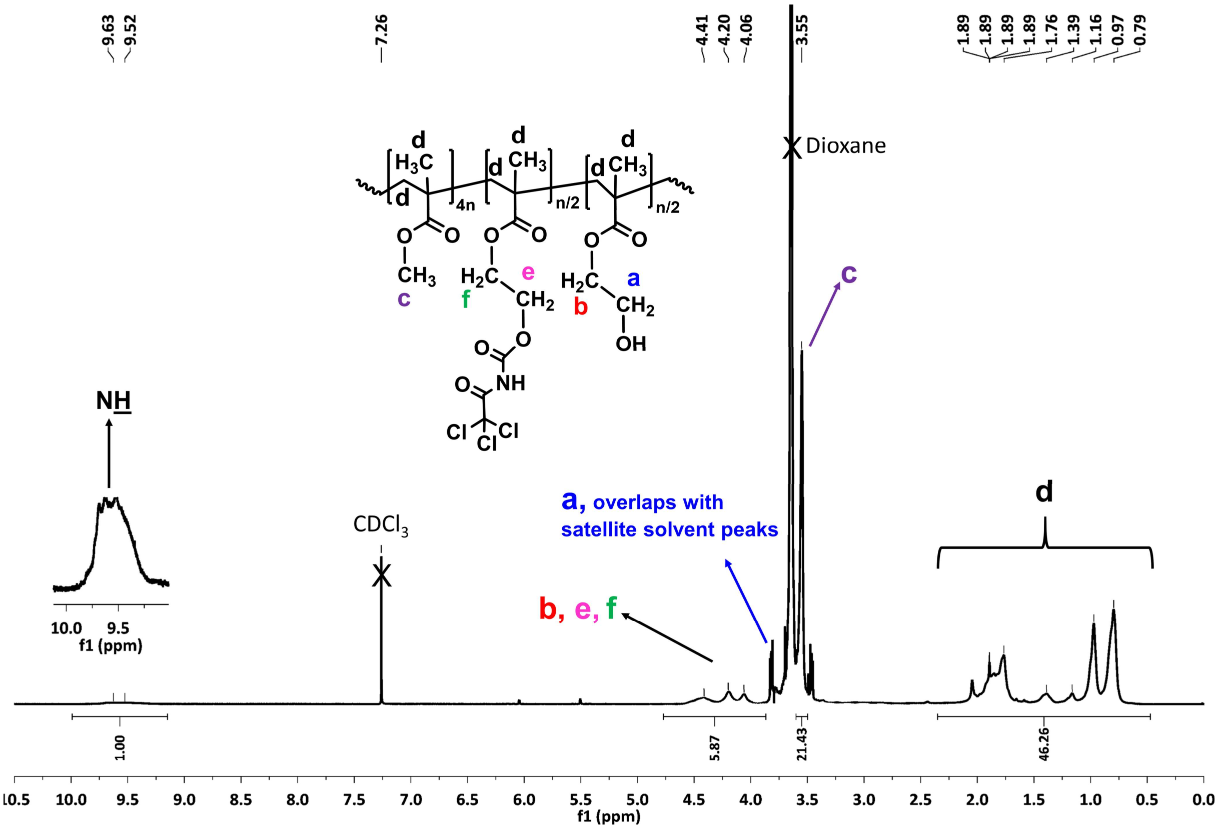


**Figure S22.** $^1$H NMR (reaction mixture; $CDCl_3$) of poly(MMA-*co*-HEMA) synthesized by MTAC-initiated Cu(0)-RDRP in dioxane before (top) and after (bottom) modification of a part of the hydroxyl groups by TAI (steps 1 and 2 in the *de novo* one-pot synthesis of a graft copolymer as per Figure 3).

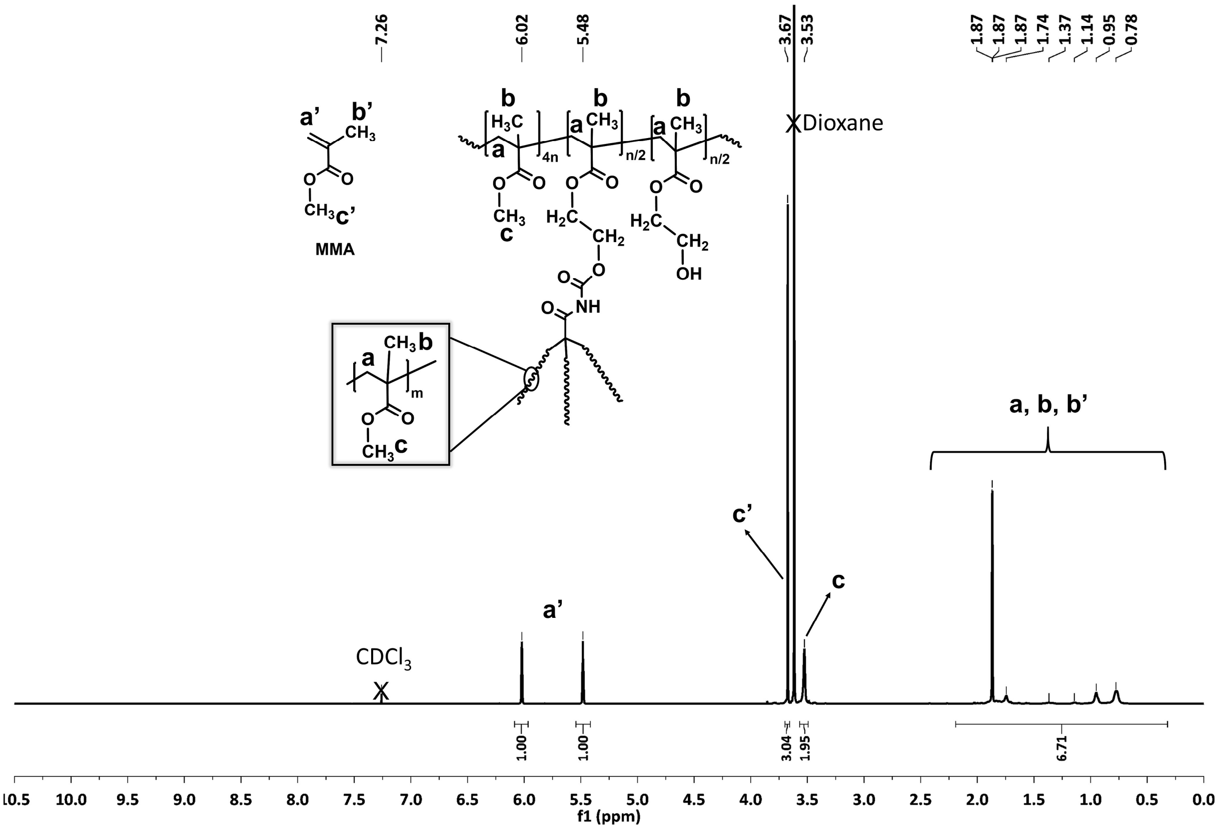


**Figure S23.** $^{1}H$ NMR (reaction mixture; $CDCl_3$) of poly(MMA-*co*-HEMA)-*graft*-poly(MMA) synthesized via Cu(0)-RDRP of MMA initiated by the TAI-modified poly(MMA-*co*-HEMA) copolymer (step 3 in the *de novo* one-pot synthesis of a graft copolymer as per Figure 7).

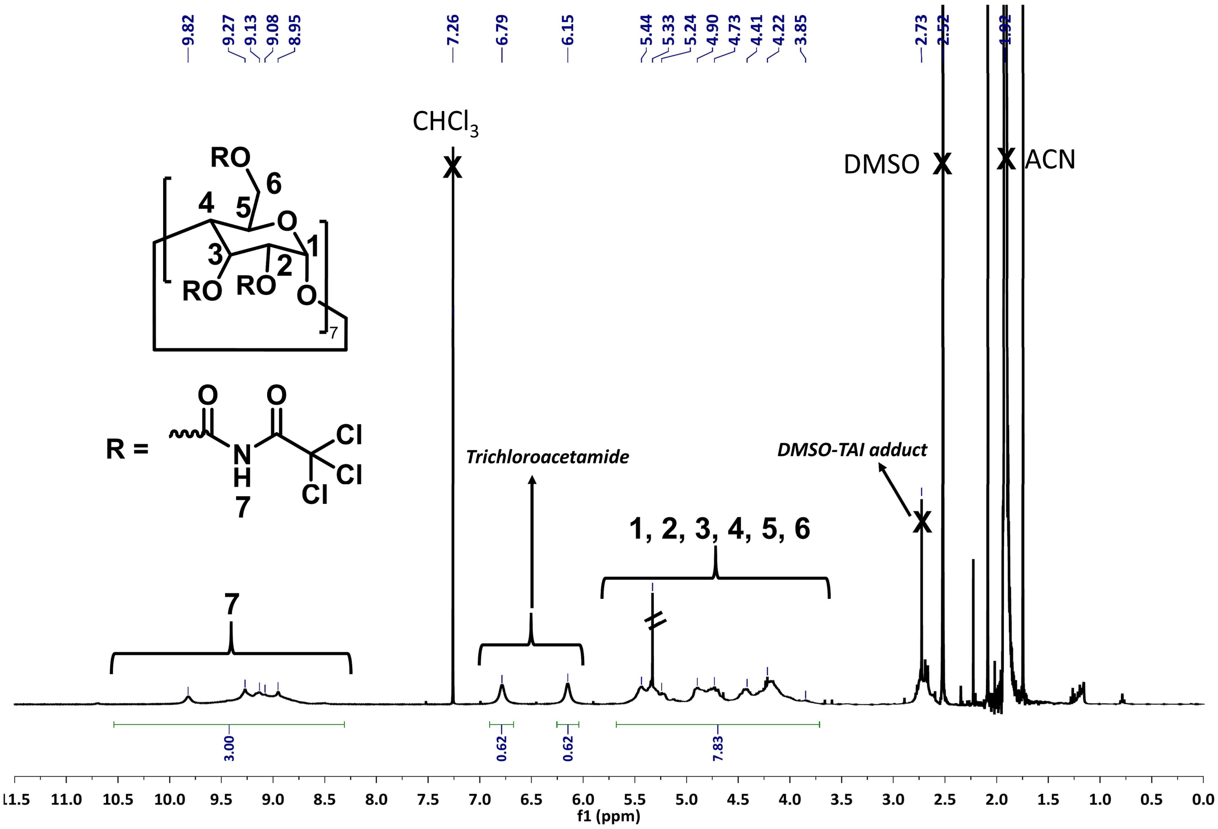


**Figure S24.** $^1$H NMR (reaction mixture; $CDCl_3$) of ß-CD modified with TAI in acetonitrile.

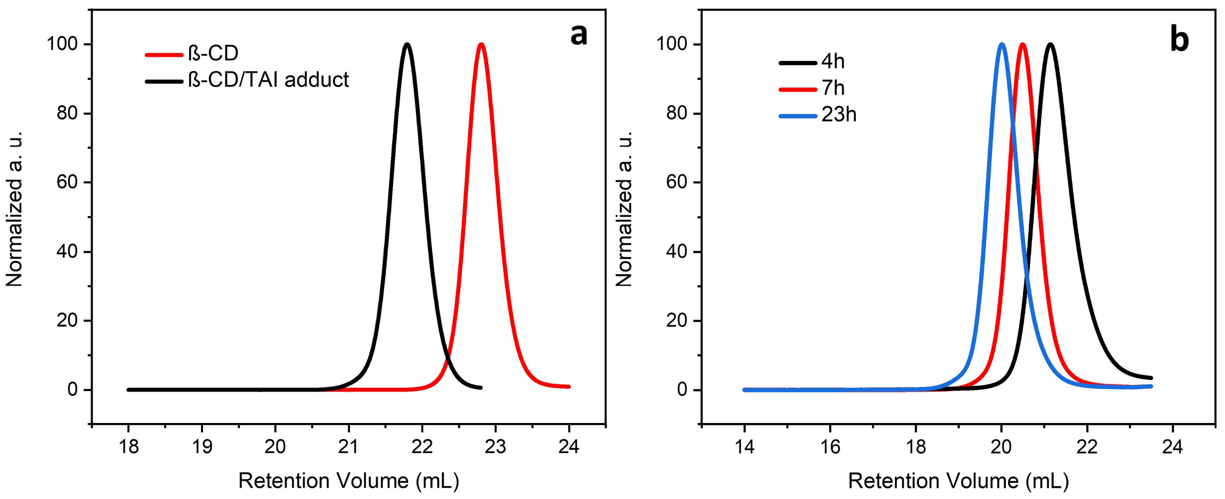


**Figure S25**. TD-SEC analysis (RI traces) of **(a)** ß-CD and ß-CD/TAI adduct ($M_n$(theor.) = 5 100, $M_n$(SEC) = 5 800, $Đ$ = 1.02) and **(b)** the polymers obtained after alkaline hydrolysis of isolated samples of poly(MMA) star polymer synthesized via ATRP initiated by the ß-CD/TAI adduct.

**Table S6**. Synthesis of multi-arm poly(MMA) stars through ATRP initiated by the ß-CD/TAI adduct[a]

| Time (h) | Conv. (%)[b] | Star polymer | | Free-growing chains | | Hydrolysis product | | |
|---|---|---|---|---|---|---|---|---|
| | | $M_n$ (SEC)[c] | $Đ$[c] | $M_n$ (SEC)[c] | $Đ$[c] | $M_n$ (theor.)[d] | $M_n$ (SEC)[c] | $Đ$[c] |
| 4 | 46 | 157 500 | 1.05 | 14 400 | 1.06 | 6 400 | 13 000 | 1.06 |
| 7 | 69 | 255 100 | 1.08 | 18 900 | 1.06 | 9 700 | 18 400 | 1.05 |
| 23 | 96 | 399 900 | 1.15 | 26 200 | 1.07 | 13 500 | 25 500 | 1.05 |

[a] Polymerization conditions: MMA/TAI/CuBr/PMDETA = 140:1:1:1; MMA/dioxane = 1:1 (v/v), 85 °C.

[b] Monomer conversion determined by $^1$H NMR.

[c] Determined by TD-SEC.

[d] Theoretical $M_n$ calculated from the monomer/TAI ratio and conversion, assuming 100% initiation efficiency.

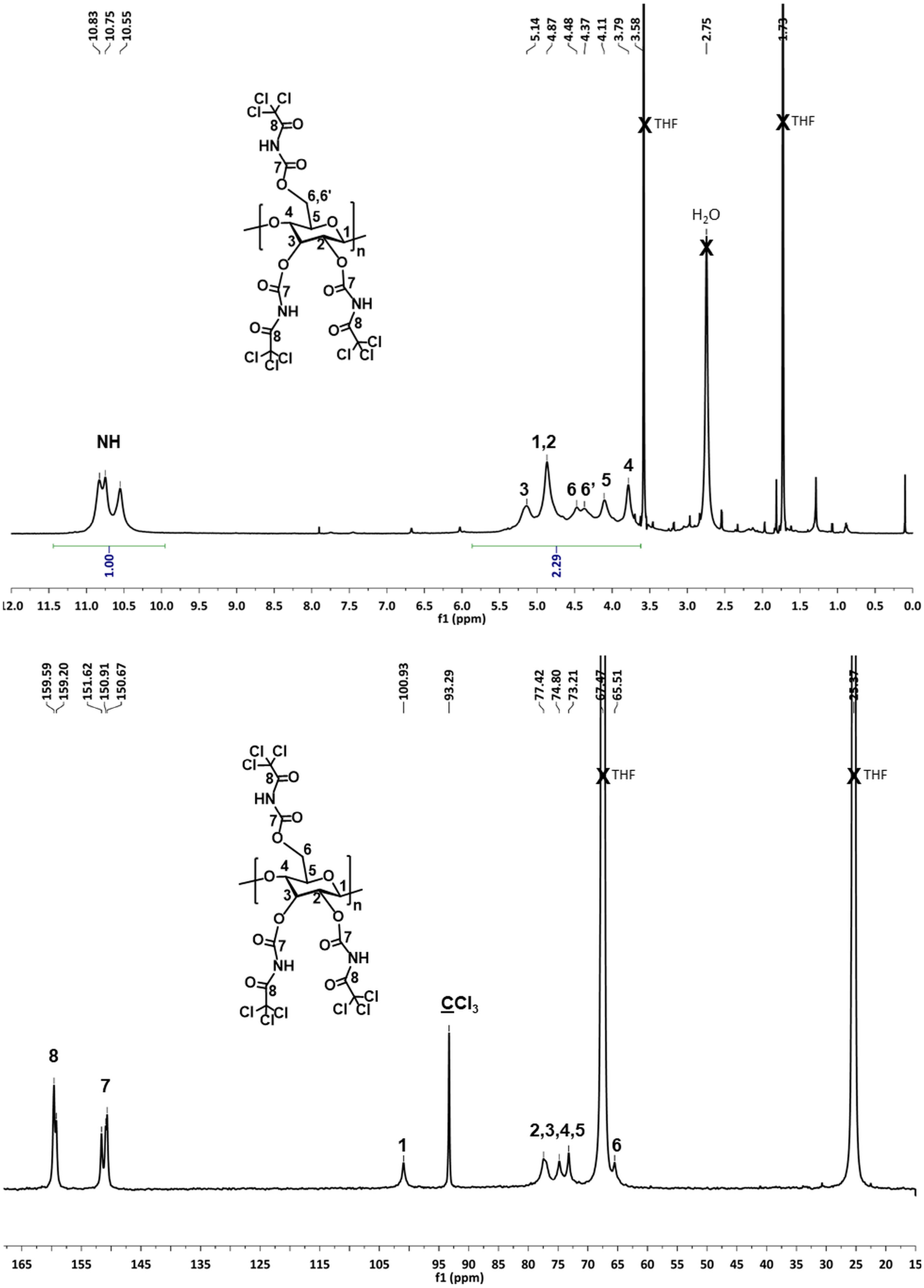


**Figure S26**. $^{1}H$ (top) and $^{13}C$ NMR (bottom) spectra (THF-$d_8$) of cellulose AVICEL PH-101 fully modified with TAI (modification performed in 7.7 wt.% DMAc/LiCl).

**Table S7.** Synthesis of ultra-dense bottle-brush cellulose-*graft*-poly(MMA) copolymers via ATRP initiated by the cellulose/TAI adduct.[a]

| Time (h) | Conv. (%)[b] | Graft copolymer | | | Free-growing chains | | Hydrolysis product | | |
|---|---|---|---|---|---|---|---|---|---|
| | | $M_n$ (theor)[c] | $M_n$ (SEC)[d] | $Đ$[d] | $M_n$ (SEC)[d] | $Đ$[d] | $M_n$ (theor)[e] | $M_n$ (SEC)[f] | $Đ$[f] |
| 5 | 27 | 9 644 000 | 3 174 000 | 1.93 | 28 300 | 1.07 | 21 600 | 24 400 | 1.06 |
| 24 | 72 | 25 539 000 | nd | nd | nd | nd | 57 700 | 62 000 | 1.11 |

[a] Polymerization conditions: MMA/TAI/CuBr/PMDETA = 800:1:1:1; MMA/dioxane = 1:1 (v/v), 85 °C; cellulose/TAI adduct macroinitiator: $M_n$ = 106 700, $Đ$ = 2.17

[b] Monomer conversion determined by $^1$H NMR.

[c] Theoretical $M_n$ calculated from the M/I ratio, monomer conversion, and the number-average degree of polymerization ($DP_n$) of 147 determined by the TD-SEC of the cellulose/TAI adduct (monomeric unit weight of 727.34), assuming that three TAI-modified hydroxyl groups per one monomeric unit initiate the polymerization.

[d] Determined by TD-SEC using universal calibration.

[e] Theoretical $M_n$ calculated from the monomer/TAI ratio and monomer conversion.

[f] Determined by TD-SEC using a light scattering detector.

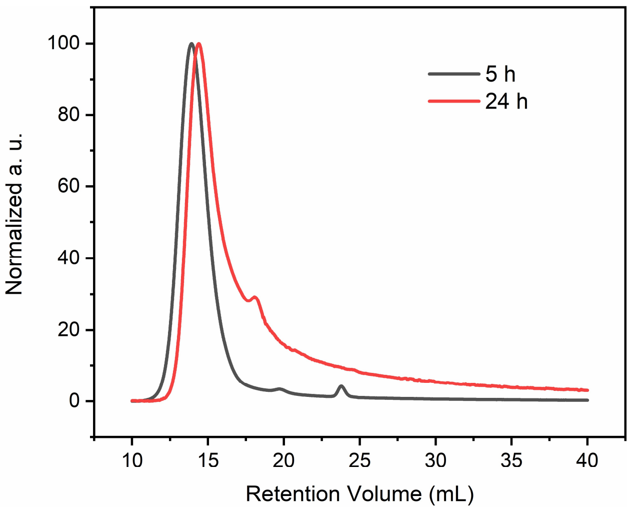


**Figure S27**. Light scattering signals from the TD-SEC analysis of samples of cellulose-*graft*-poly(MMA) bottle-brush graft copolymers taken at different polymerization time.

Due to an unknown non-SEC elution mechanism,[5] a high-MW fraction of the sample shows delayed elution, co-eluting with lower-MW fractions. This impacts significantly on the light scattering signal traces, resulting in the blending of the peaks of the graft copolymer and free-growing chains (this is particularly pronounced for the 24 h sample). Consequently, the light scattering signal intensity of the graft copolymer is diminished to an unknown extent while the signal intensity of the free-growing chains is markedly inflated. As a result, the calculation of MWs based on light scattering data is highly inaccurate or even impossible (a software limitation). Due to the comparatively lower sensitivity of the viscometric detector to the high-MW species, we were able to obtain relatively reliable MW values for the low-MW peak of the 5 h sample using the universal calibration approach (**Table S7**).

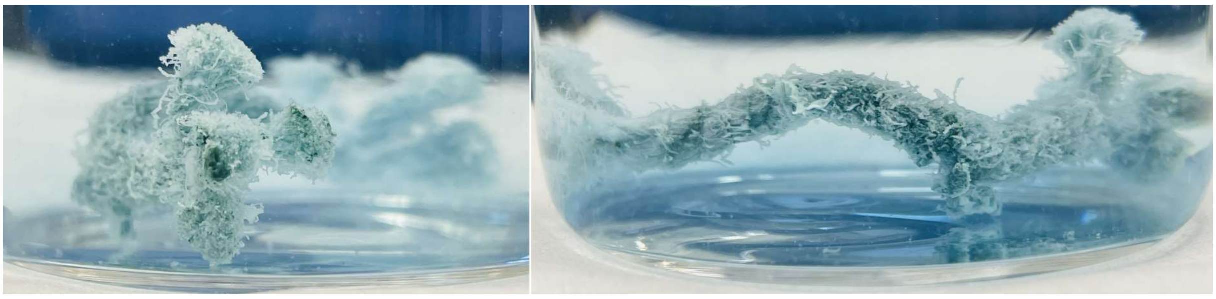

**Figure S28.** Surface-initiated ATRP grafting of poly(MMA) from a cotton thread modified with TAI. The photographs were taken during the purification of the grafted thread (washing in methanol).

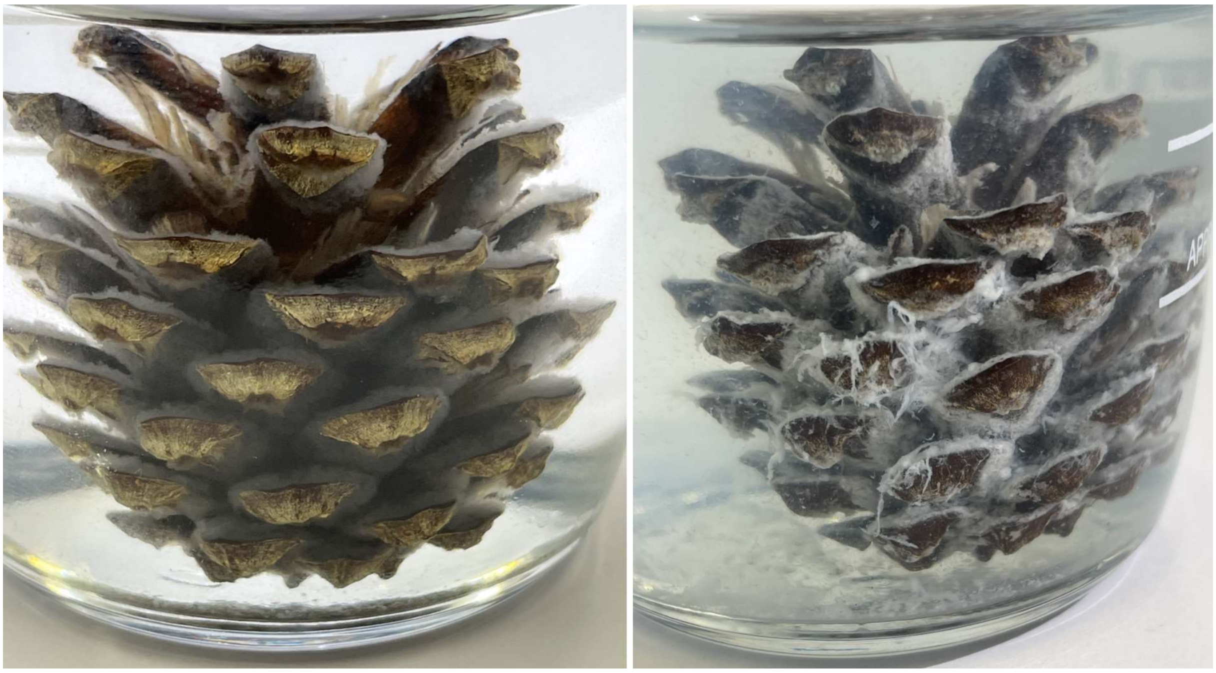

**Figure S29.** Surface-initiated ATRP grafting of poly(MMA) from the pine tree cone modified with TAI. The photographs were taken during the purification of the grafted cone (left – washing in THF, right - washing in methanol).

## REFERENCES


[1] G. J. P. Britovsek, J. England, A. J. P. White, *Inorg. Chem.* **2005**, *44*, 8125-8134.

[2] V. Raus, M. Štěpánek, M. Uchman, M. Šlouf, P. Látalová, E. Čadová, M. Netopilík, J. Kříž, J. Dybal, P. Vlček, *J. Polym. Sci., Part A: Polym. Chem.* **2011**, *49*, 4353-4367.

[3] V. Raus, A. Sturcova, J. Dybal, M. Slouf, T. Vackova, P. Salek, L. Kobera, P. Vlcek, *Cellulose* **2012**, *19*, 1893-1906.

[4] L. W. Dittert, T. Higuchi, *J. Pharm. Sci.* **1963**, *52*, 852-857.

[5] M. Gerle, K. Fischer, S. Roos, A. H. E. Müller, M. Schmidt, S. S. Sheiko, S. Prokhorova, M. Möller, *Macromolecules* **1999**, *32*, 2629-2637.
